# Synthesis, Computational Study, and In Vitro α-Glucosidase Inhibitory Action of 1,3,4-Thiadiazole Derivatives of 3-Aminopyridin-2(1*H*)-ones

**DOI:** 10.3390/ph17030377

**Published:** 2024-03-15

**Authors:** Zarina Shulgau, Irina V. Palamarchuk, Shynggys Sergazy, Assel Urazbayeva, Yerlan Ramankulov, Ivan V. Kulakov

**Affiliations:** 1 National Laboratory Astana, Nazarbayev University, Kabanbai Batyr Ave. 53, Astana Z05H0P9, Kazakhstan; shynggys.sergazy@gmail.com (S.S.);; 2 National Center for Biotechnology, 13/5 Kurgalzhynskoe Road, Astana Z05K8D5, Kazakhstan; 3 Higher School of Natural Science, University of Tyumen, 15a Perekopskaya St., Tyumen 625003, Russia; i.v.palamarchuk@utmn.ru; 4 School of Science and Technology, Nazarbayev University, Kabanbai Batyr Ave. 53, Astana Z05H0P9, Kazakhstan

**Keywords:** monothiooxamides, oxamic acid thiohydrazides, 3-aminopyridin-2(1*H*)-ones, 1,3,4-thiadiazole derivatives, *α*-glucosidase inhibition, antidiabetic activity, IC_50_, molecular docking

## Abstract

This article reports on the synthesis of nine promising new 1,3,4-thiadiazole derivatives based on 3-aminopyridones, containing various acidic linkers. The synthesis was carried out by cyclizing the corresponding thiohydrazides **4a**–**c** and anhydrides of glutaric, maleic, and phthalic acids upon heating in acetic acid solution. The conducted bio-screening of the synthesized new 1,3,4-thiadiazole derivatives containing different acidic linkers (butanoic, acrylic, and benzoic acids) showed that they have significant inhibitory activity against *α*-glucosidase (up to 95.0%), which is 1.9 times higher than the value for the reference drug acarbose (49.5%). Moreover, one of the 1,3,4-thiadiazole derivatives with a benzoic acid linker—2-(5-((6-Methyl-2-oxo-4-(thiophen-2-yl)-1,2-dihydropyridin-3-yl)carbamoyl)-1,3,4-thiadiazol-2-yl)benzoic acid (**9′b**)—showed an IC_50_ value of 3.66 mM, nearly 3.7 times lower than that of acarbose (IC_50_ = 13.88 mM). High inhibitory activity was also shown by 1,3,4-thiadiazole derivatives with a butanoic acid linker (compounds **7b**, **7c**)—with IC_50_ values of 6.70 and 8.42 mM, respectively. A correlation between the structure of the compounds and their activity was also established. The results of molecular docking correlated well with the bioanalytical data. In particular, the presence of a butanoic acid linker and a benzoic fragment in compounds **7b**, **7c**, and **9b** increased their binding affinity with selected target proteins compared to other derivatives **3**–**6** (**a**–**c**). Calculations according to Lipinski’s rule of five also showed that the synthesized compounds **7b**, **7c**, and **9b** fully comply with Ro5 and meet all criteria for good permeability and acceptable oral bioavailability of potential drugs. These positive bioanalytical results will stimulate further in-depth studies, including in vivo models.

## 1. Introduction

It is well known that Diabetes Mellitus (DM) is associated with metabolic disorders and is characterized by the development of hyperglycemia due to insulin secretion deficiency [1]. Approximately 90% of all patients with diabetes suffer from type 2 diabetes (DM2). Currently, the World Health Organization (WHO) characterizes DM2 as a progressive chronic endocrine disease. Unfortunately, DM2 is accompanied by significant mortality if not properly controlled [2,3]. The prognosis for diabetes’ prevalence in the near future is quite discouraging, especially in developing countries [4]. Moreover, it is upsetting that children have become increasingly affected by DM2 in recent years. According to the website “Statistica. Health & Pharmaceuticals State of Health”, currently about 425 million people worldwide live with diabetes. By 2045, it is projected that this number will increase to 629 million people with diabetes! [5]. Consequently, the treatment of diabetes can be considered a global problem in science and healthcare.

One of the main therapeutic approaches to combatting diabetes is the slowing of postprandial hyperglycemia by reducing glucose absorption through the inhibition of enzymes that hydrolyze carbohydrates (alpha-(*α*)-amylases and *α*-glucosidases) in the gastrointestinal tract.

The two main enzymes involved in carbohydrate digestion are *α*-glucosidase and *α*-amylase [6]. The mechanisms of action of these enzymes involve the breakdown of carbohydrates by *α*-amylase, while *α*-glucosidase breaks down starch and disaccharides into glucose [7].

*α*-Amylase is an enzyme that hydrolyzes starch molecules into small glucose units (in a small polymer), leading to hyperglycemia and the development of DM2 [8].

The second enzyme, *α*-glucosidase, is a key carbohydrate hydrolase that regulates blood glucose levels by specific hydrolysis of the 1,4-*α*-glycosidic bond, forming *α*-glucose [9]. Inhibition of *α*-glucosidase activity can slow down glucose absorption and reduce postprandial blood glucose levels. Therefore, *α*-glucosidase is considered a key target for diabetes treatment, and *α*-glucosidase inhibitors can be developed into effective therapeutic drugs for this disease [10].

**Figure 1:**
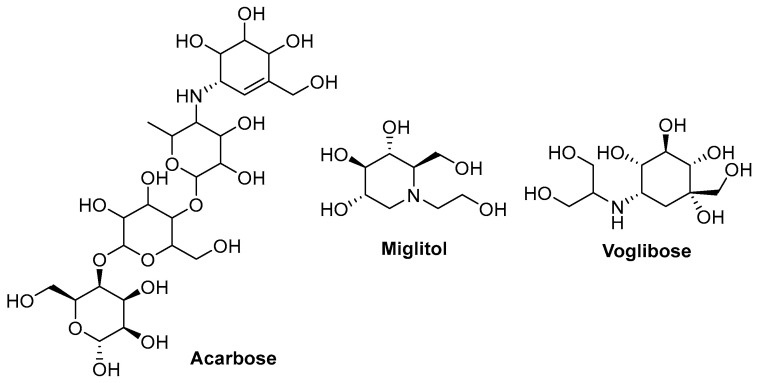
Acarbose, miglitol, and voglibose structures.

Currently, only three *α*-glucosidase inhibitors are used in medical practice: acarbose, miglitol, and voglibose (Figure 1).

In recent years, the research efforts of many medical scientists, pharmacologists, and organic chemists have been focused on the search for a new class of safe and highly effective inhibitors of *α*-glucosidase and *α*-amylase. The search is directed towards modifying natural compounds as well as developing new synthetic organic compounds of various classes. Quite often, targeted methods of virtual programming and molecular docking are used when searching for new advanced antidiabetic drugs [11,12].

However, no matter how successful molecular docking is and how well the target is chosen, a new starting molecule is always necessary, the modification of which can lead to a lead compound. In this regard, routine classical organic synthesis, including the synthesis of new compound classes, is certainly necessary. Derivatives containing the necessary pharmacophoric groups (mainly sulfur- and nitrogen-containing compounds such as thioureas, sulfonoureas, thiadiazoles, guanidines, and their various derivatives) deserve attention [13,14].

Many researchers have high hopes for the unique properties of 1,3,4-thiadiazoles, which possess high-potential biological activities (antidiabetic, antibacterial, antiparasitic, antioxidant, antidepressant, anticonvulsant, and antitumor activity) [15,16,17,18,19]. 1,3,4-Thiadiazole is a bioisostere of pyrimidine and oxadiazole, and its derivatives likely have significant therapeutic potential, including relative biocompatibility and low toxicity.

Thus, the search for new inhibitors of α-glucosidase with potential antidiabetic activity, including those based on derivatives of 1,3,4-thiadiazole, is a relevant task within medicinal chemistry.

## 2. Results and Discussion

### 2.1. Chemistry

The choice of the 3-aminopyridin-2(1*H*)-ones **1a**–**c** as starting materials, the synthesis of which is described by us in [20], is associated with their limited biological knowledge and impressive data on the bioactivity of their derivatives. In our previous work [21], it was demonstrated that some synthesized derivatives of 3-(aryl-methylamino)pyridinone exhibit very high anti-radical activity and high cytoprotective potential, improving cell survival in vitro, including in the MTT test on human dermal fibroblasts, and enhancing the hemorheological properties of blood. The same derivatives of 3-(aryl-methylamino)pyridinone [22] were found to possess anxiolytic activity in the “light–dark box” test and antidepressant activity in the “forced swim test” according to the Porsolt test, surpassing the activity of reference drugs (mexidol and amitriptyline). The established high pharmacological activity of the new derivatives of 3-aminopyridin-2(1*H*)-ones [21,22,23] opens up vast possibilities and prospects for the use of these structures in medical practice as potential drugs with high geroprotective activity.

In our recently published work [24], three laboratory-accessible derivatives of 3-aminopyridin-2(1*H*)-one **1a**–**c** were used to obtain mono-thioxamide derivatives **3**, **4a**–**c**.

**Scheme 1:**
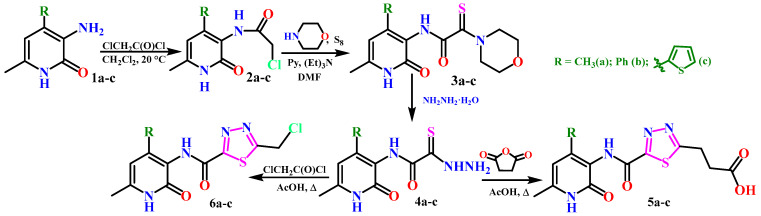
Synthesis of thiohydrazides **4a**–**c** and 1,3,4-thiadiazole derivatives.

Subsequently, through the cyclization of thiohydrazides **4a**–**c** using 2-chloroacetyl chloride and succinic anhydride, the first derivatives of 1,3,4-thiadiazole **5** and **6a**–**c** with a pyridone framework were obtained (Scheme 1) [24].

The synthesized derivatives of 1,3,4-thiadiazoles **5** and **6a**–**c** showed sufficiently high antidiabetic activity in vitro against *α*-amylase and *α*-glucosidase enzymes, as well as low IC_50_ values. Furthermore, it was found that all 12 synthesized compounds, **3**(**a**–**c**), **4**(**a**–**c**), **5**(**a**–**c**), **6**(**a**–**c**), exhibited not only low cytotoxicity but also pronounced cytoprotective properties in the cytotoxicity assay (MTT test) [24].

These results prompted us to further chemically modify thiohydrazides **4a**–**c** to obtain new derivatives of 1,3,4-thiadiazole with different acidic linkers and subsequently investigate the structure-antidiabetic activity relationship.

**Scheme 2:**
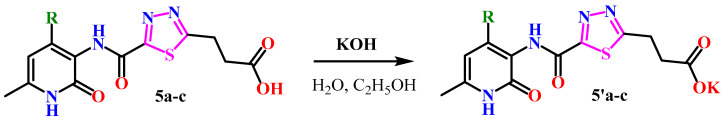
Synthesis of salts of 1,3,4-thiadiazole derivatives.

Additionally, with the aim of further studying their bioactivity, increasing bioavailability and water solubility, and potential application as pharmaceuticals based on 1,3,4-thiadiazole derivatives, the corresponding salts of **5a**–**c** were obtained (Scheme 2).

**Scheme 3:**
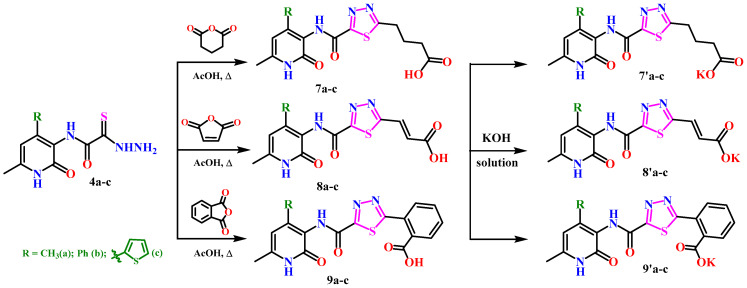
Synthesis of 1,3,4-thiadiazole derivatives **7**–**9a**–**c**.

In order to obtain new research objects based on the previously synthesized thiogidrazides **4a**–**c**, condensation was performed using glutaric, maleic, and benzoic anhydrides to yield the corresponding 1,3,4-thiadiazoles **7**–**9a**–**c**. The cyclization reaction was carried out by heating in acetic acid for a duration of 5 h (Scheme 3).

The obtained derivatives of 1,3,4-thiadiazole **7**–**9a**–**c** are white (or beige) powdered substances with high melting points. Moreover, all compounds, due to the presence of the carboxylic group, readily dissolve in alkaline aqueous solutions, forming water-soluble salts. The corresponding salts **7′**, **8′**, **9′a**–**c** were obtained for biological testing.

The structures of all obtained derivatives were confirmed by ^1^H, ^13^C NMR spectroscopy and High-Resolution Mass Spectrometry (Supplementary Materials).

**Scheme 4:**
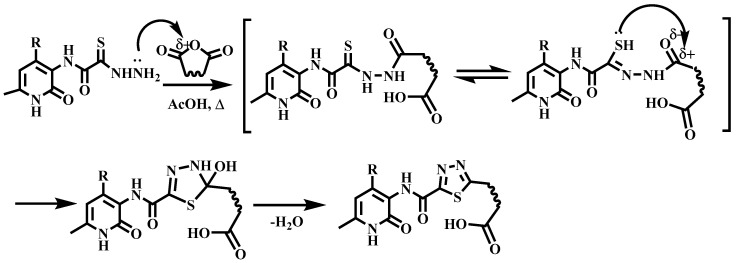
Proposed mechanism of cyclization.

The proposed mechanism of thiohydrazide cyclization, based on the nucleophilic addition of the β-hydrazide nitrogen atoms and the sulfur atom in thiol form to the carbonyl atom of the anhydride, is presented in Scheme 4.

The formation of 1,3,4-thiadiazoles **7**–**9a**–**c** is consistent with the general principles of Pearson’s HSAB theory. Thus, the nucleophilic fragment -RNH_2_ acts as a hard base and attacks the carbonyl carbon of the -R-C=O fragment, which is a hard acid.

### 2.2. In Vitro α-Glucosidase Inhibition Assay

To check the known literature data on the antidiabetic activity of sulfur-containing derivatives, including thiadiazole heterocycles, we carried out screening studies for the presence of antidiabetic activity on compounds **5′**–**9′a**–**c**.

Antidiabetic activity was assessed by the degree of inhibition of α-glucosidase activity by the test substances.

The study of the degree of *α*-glucosidase activity inhibition by the test compounds was performed using a standard method with minor modifications [25].

The results of the study on the inhibitory activity of the test compounds against the *α*-glucosidase enzyme are shown in Table 1.

The analysis of the “structure–activity” relationship showed that elongating the acidic linker from propionic acid to butanoic acid (compound **7′a**–**c**) for the starting structures **5′a**–**c** [24] not only significantly increased the degree of inhibition (from 36% to 95%), but also decreased the IC_50_ value (down to 6.7 µg/mL for **7′b**). The introduction of an aromatic acid linker also led to an increase in the degree of inhibition (up to 95% for **9′b**) and a decrease in the IC_50_ value (to a minimum of 3.66 µg/mL for **9′b**). The introduction of an acryloyl acid linker practically did not increase the degree of inhibition compared to the starting molecules with a propionic acid linker (**5′a**–**c**). Furthermore, the presence of a phenyl substituent (and a 2-thiophene fragment) at the fourth position on the pyridone ring in the series of derivatives of 1,3,4-thiadiazole **5′**–**9′a**–**c** significantly increased the activity. The compound **9′b** exhibited the highest inhibitory activity, as well as the minimum IC_50_ value (3.7 µg/mL).

### 2.3. Molecular Docking

Molecular docking, also known as “Virtual Screening” (VS), is considered to be a powerful tool for predicting or confirming observed experimental results. Currently, molecular docking is widely used in structural molecular biology and computer-aided drug design to search for and predict possible dominant binding mechanisms between a docked compound (ligand) and a biological target protein. This method is also successfully used in studying the binding mechanisms of many α-glucosidase inhibitors [26,27,28].

In this study, three target receptors (i.e., lysosomal *α*-glucosidase, *α*-glucosidase, and α-amylase) were chosen because they play an important and critical role in maintaining glucose levels in the body [29]. To assess the assumed antidiabetic activity of the synthesized derivatives **5**–**9a**–**c**, we used the molecular docking method. The enzymes *α*-glucosidase (PDB identifier: 3W37) [27], lysosomal *α*-glucosidase (PDB: 5NN8) [30], and *α*-amylase (PDB: 2QV4) [31] were used.

The three-dimensional (3D) structures of these enzymes were obtained from the RCSB Protein Data Bank [32], and the ligand molecules were modeled using ChemBio3D Ultra 14.0 software. The protein structures were prepared for docking by removing water the molecules and native ligands, adding polar hydrogen atoms, and converting them to the pdbqt format using the AutoDock MGL v1.5.7 [33] software package. The docking process was performed using the AutoDock Vina program v1.5.7 [34]. For the enzyme *α*-glucosidase (PDB: 3W37), the coordinates of the active site grid were X = 2.0, Y = −3.0, and Z = −22.0 (size 22 × 22 × 22 Å); for the enzyme *α*-glucosidase (PDB: 5NN8), the coordinates of the active site grid were X = −11.00, Y = −38.95, and Z = 94.39 (25 × 25 × 25 Å); and for the enzyme *α*-amylase (PDB: 2QV4) the coordinates were X = 14.0, Y = 45.0, and Z = 24.0 (25 × 25 × 25 Å). The interactions between ligands in the binding sites were interpreted using the Discovery Studio Visualizer v21.1.0.20298 software package [35].

The docking results (Table 2) indicated that for the studied structures, the free energies (kcal/mol) of complexes with selected receptors (3W37, 2QV4, 5NN8) do not significantly exceed the free energies of complexes of these proteins with their respective native ligands.

The number of intermolecular hydrogen bonds, the binding energy of stable complexes, and the number of nearest amino acid residues were determined for compound **9b**, which exhibited a high binding affinity (Table 3). Other interactions of compounds **5**–**8a**–**c**, **9a**,**c** are presented in (Table S2. Supplementary Materials).

**Figure 2:**
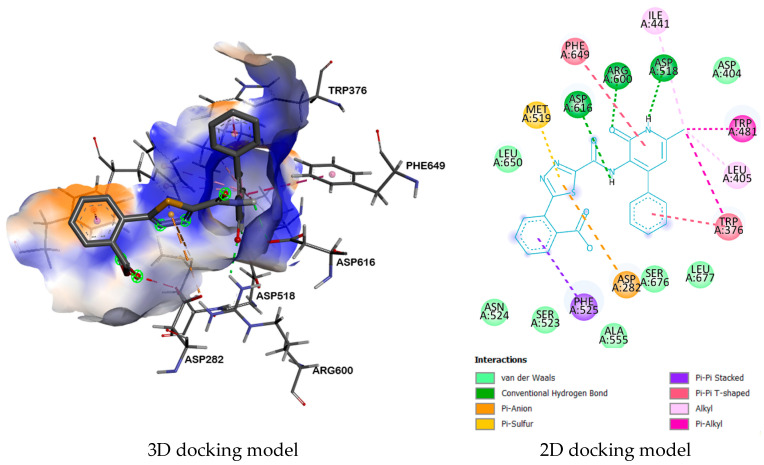
Complex between **9b** and enzyme *α*-glucosidase (5NN8).

It was found that the structure of **9b** has a high binding energy (−8.8 kcal/mol) with enzyme *α*-glucosidase (5NN8), due to the formation of two π-π T-shaped interactions between the amino acid residues TRP376 and PHE649 and the phenyl ring and the aminopyridone fragment. Additionally, three hydrogen bonds were formed: one bond between the carbonyl oxygen atom of the pyridone ring and the amino acid residue ARG600, and one between the hydrogen atoms of the carboxyl, two amide groups, and the amino acids ASP616 and ASP518 (Figure 2).

**Figure 3:**
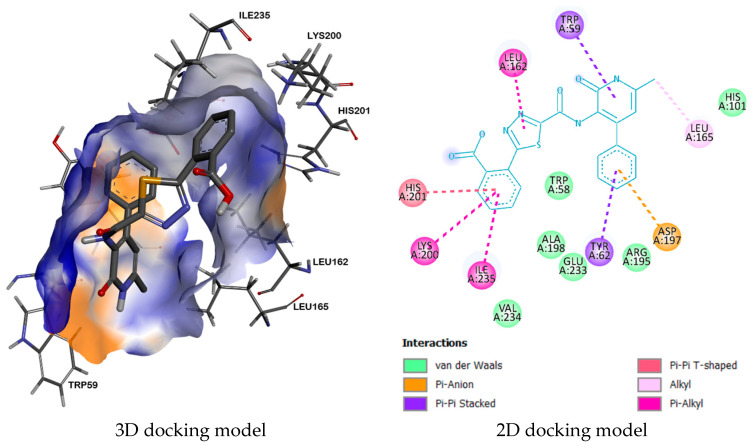
Complex between **9b** and enzyme *α*-amylase (2QV4).

Furthermore, the complex of compound **9b** with enzyme *α*-amylase (2QV4) has a high binding energy (−9.1 kcal/mol) due to the formation of two π-π-stacked interactions between the amino acid residues TYR62 and TRP59 and the phenyl ring and the aminopyridone fragment. In addition, π-alkyl interactions are observed between the amino acid residues LYS200, ILE235, and LEU162 and the benzoyl and 1,3,4-thiadiazole fragments. Moreover, six Van der Waals interactions are formed: HIS305, TRP58, ASP300, ALA198, GLU233, ARG195 (Figure 3).

**Figure 4:**
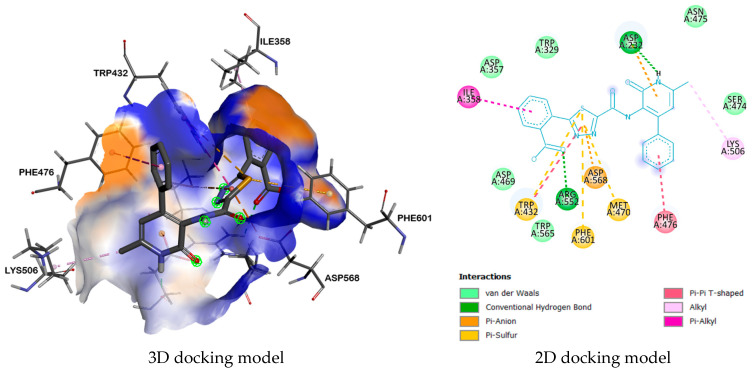
Complex between **9b** and enzyme *α*-glucosidase (3W37).

Analysis of the interaction of compound **9b** with *α*-glucosidase enzyme (PDB identifier: 3W37) showed that the resulting complex has a higher binding energy (−9.0 kcal/mol) due to the formation of a parallel π-π T-shaped interaction between the amino acid residue PHE476 and the phenyl ring. Additionally, a π-alkyl interaction is observed between the amino acid residue ILE358 and the benzoyl fragment. The sulfur atom of the 1,3,4-thiadiazole ring also forms a π-sulfur interaction with the residues PHE601 and TRP432. Furthermore, the amino acid residues ASP232 and ASP568 form π-anion interactions with the pyridone and 1,3,4-thiadiazole rings, respectively. In addition, two hydrogen bonds are formed between the nitrogen atom of the pyridone ring amino group and the residue ASP232, and there is also the formation of a hydrogen bond between the oxygen atom of the carbonyl group of the benzoyl fragment and ARG552 (Figure 4).

**Figure 5:**
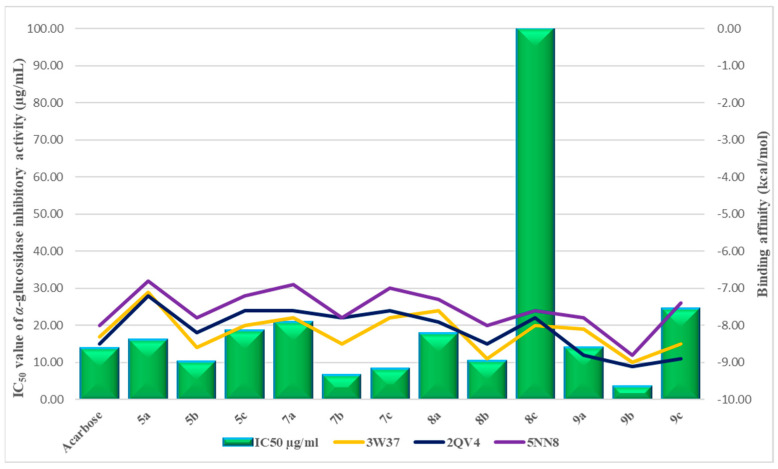
Half maximal inhibitory concentration (IC_50_) in assay with α- glucosidase (green) and binding energies for compound (**5**–**9a**–**c**).

A comparative analysis of the molecular docking data on receptor proteins (3W37, 2QV4, and 5NN8) with the results of α-glucosidase enzyme inhibition, including IC_50_ (Table 1), demonstrated a sufficiently good correlation for three leading compounds—**7b**, **7c**, and **9b** (Figure 5).

Thus, molecular docking studies revealed that the presence of a butanoic acid linker and a benzoyl fragment in the 1,3,4-thiadiazole cycle of compounds **7b**, **7c**, **9b** contributes to stronger interactions of these compounds in the binding pocket of *α*-glucosidase and *α*-amylase enzymes, stabilizes the ligand-receptor complex, and consequently increases their affinity for the selected receptors compared to other derivatives, such as **3**(**a**–**c**), **4**(**a**–**c**), **5**(**a**–**c**), **6**(**a**–**c**), previously obtained and investigated in our work [24]. Additionally, the presence of a phenyl substituent at the fourth position on the pyridone ring in compounds **7b** and **9b** increases their binding affinity.

Computational studies were also conducted using the ADMETlab [36] tool to evaluate the bioavailability and physicochemical properties of synthesized compounds **7b**, **7c**, **9b**. The results of these calculations allow for an assessment of the potential applications of the newly synthesized compounds as orally active drugs.

Orally active drugs should not violate more than one of the following criteria according to Lipinski’s rule: no more than five hydrogen bond donors, no more than ten hydrogen bond acceptors, a molecular weight not exceeding 500 Da, and an octanol–water distribution coefficient (log P) that does not exceed five [37,38,39]. The obtained results are presented in Table 4.

According to Lipinski’s rule, the synthesized compounds **7b**, **7c**, **9b** satisfy the Ro5 (≤1 violations) and meet all criteria for good permeability and acceptable oral bioavailability, which together may indicate their potential use as antidiabetic drugs.

## 3. Materials and Methods

The description of this section (figures of spectrums) is included as Supplementary Material.

^1^H and ^13^C NMR spectra were recorded on Bruker DRX400 (Rheinstetten, Germany) (400 and 100 MHz, respectively), Bruker AVANCE 500 (Burladingen, Germany) (500 and 125 MHz, respectively), and Magritek spinsolve 80 carbon ultra (Aachen, Germany) (81 and 20 MHz, respectively) instruments; using DMSO-*d*_6_, the internal standard was TMS or residual solvent signals (2.49 and 39.9 ppm ^1^H and for ^13^C nuclei in DMSO-*d*_6_).

Samples were analyzed by HPLC-MS on an Agilent 1260 Infinity II chromatograph coupled to an Agilent 6545 LC/Q-TOF high-resolution mass spectrometer with a Dual AJS ESI ionization source operating in positive-ion mode using the following parameters: capillary voltage: 4000 V; spray pressure: 20 (psi); drying gas: 10 L/min; gas temperature: 325 °C; sheathed gas flow: 12 L/min; shielding gas temperature: 400 °C; nozzle voltage: 0 V, fragmentation voltage: 180 V; skimmer voltage: 45 V; octopole RF: 750 V. Mass spectra with LC/MS accuracy were recorded in the range 100–1000 *m*/*z*, with a scan rate of 1.5 spectrum/s.

Chromatographic separation was carried out on columns: ZORBAX RRHD Eclipse Plus C18 (2.1 × 50 mm, particle size 1.8 µm). The column temperature during the analysis was maintained at 35 °C. The mobile phase was formed by eluents A and B. In the positive-ionization mode, 0.1% formic acid solution in deionized water was used as eluent A, and 0.1% formic acid solution in acetonitrile was used as eluent B. Chromatographic separation was performed with elution according to the following scheme: 0–10 min 95% A, 10–13 min 100% B, 13–15 min 95% A. The flow of the mobile phase was maintained at 400 μL/min throughout the analysis. In all experiments, the sample injection volume was 1 μL. The sample was prepared by dissolving the entire sample (in 1000 μL) in methanol (for HPLC). Sample dilution was carried out immediately before analysis.

The recorded data were processed using Agilent MassHunter 10.0 software.

Melting points were determined using a Stuart SMP10 hot bench. Monitoring of the reaction course and the purity of the products was carried out by TLC on Sorbfil plates and visualized using iodine vapor or UV light.

### 3.1. Experimental Procedures

Synthesis of compounds N-(4,6-dimethyl-2-oxo-1,2-dihydropyridin-3-yl)-2-hydrazinyl-2-thioxoacetamide (**4a**), 2-hydrazinyl-N-(6-methyl-2-oxo-4-phenyl-1,2-dihydropyridin-3-yl)-2-thioxoacetamide (**4b**), 2-hydrazinyl-N-(6-methyl-2-oxo-4-(thiophen-2-yl)-1,2-dihydropyridin-3-yl)-2-thioxoacetamide (**4c**), 3-(5-((4,6-dimethyl-2-oxo-1,2-dihydropyridin-3-yl)carbamoyl)-1,3,4-thiadiazol-2-yl)propanoic (**5a**), 3-(5-((6-methyl-2-oxo-4-phenyl-1,2-dihydropyridin-3-yl)carbamoyl)-1,3,4-thiadiazol-2-yl)propanoic acid (**5b**), and 3-(5-((6-methyl-2-oxo-4-(thiophen-2-yl)-1,2-dihydropyridin-3-yl)carbamoyl)-1,3,4-thiadiazol-2-yl)propanoic acid (**5c**) is given in the article [24].

*Synthesis of thiadiazoles derivatives* **7**–**9a**–**c**

To 1 mmol of oxamic acid thiohydrazide 4a–c in 3 mL of acetic acid we added 3.0 mmol of the corresponding anhydride (glutaric acid, phthalic acid, or maleic acid). The reaction mixture was heated at reflux temperature with vigorous stirring over 5 h, cooled down, and poured into water (25 mL). The resulting precipitates were filtered off and dried in air to obtain compounds **7**–**9a**–**c** in the indicated yields.

4-(5-((4,6-Dimethyl-2-oxo-1,2-dihydropyridin-3-yl)carbamoyl)-1,3,4-thiadiazol-2-yl)butanoic acid (**7a**). Beige powder, yield 192 mg, 80%. M.p. 248–250 °C. ^1^H NMR (400 MHz, DMSO-*d*_6_) δ ppm 1.98 (p, *J* = 7.3 Hz, 2H, 3-CH_2_); 2.02 (s, 3H, CH_3_); 2.14 (s, 3H, CH_3_); 2.35 (t, *J* = 7.1 Hz, 2H, 2-CH_2_); 3.19 (t, *J* = 7.1 Hz, 2H, 4-CH_2_); 5.93 (s, 1H, H-5); 9.92 (s, 1H, NHCO’); 11.79 (br.s, 2H, NHCO, COOH). ^13^C NMR (100 MHz, DMSO-*d*_6_) δ ppm 18.1 (CH_3′_); 18.2 (CH_3_); 24.7 (3-CH_2_); 28.8 (4-CH_2_); 32.6 (2-CH_2_); 106.6 (C-5); 121.3; 142.6; 147.3; 156.4; 159.8; 165.23; 173.7; 174.1. HRMS *m*/*z*: calcd for C_14_H_17_N_4_O_4_S^+^[M + H]^+^: 337.0965; found: 337.0998.

4-(5-((6-Methyl-2-oxo-4-phenyl-1,2-dihydropyridin-3-yl)carbamoyl)-1,3,4-thiadiazol-2-yl)butanoic acid (**7b**). Beige powder, yield 218 mg, 72%. M.p. 243–246 °C. ^1^H NMR (400 MHz, DMSO-*d*_6_) δ ppm 1.96 (p, *J* = 7.6 Hz, 2H, 3-CH_2_); 2.24 (s, 3H, CH_3_); 2.34 (t, *J* = 7.1 Hz, 2H, 2-CH_2_); 3.16 (t, *J* = 7.6 Hz, 2H, 4-CH_2_); 6.08 (s, 1H, H-5); 7.34–7.39 (m, 3H, H-3,4,5 Ph); 7.46 (d, *J* = 7.6 Hz, 2H, H-2,6 Ph); 10.07 (s, 1H, NHCO’); 12.05 (br. s, 2H, NHCO, COOH). ^13^C NMR (100 MHz, DMSO-*d*_6_) δ ppm 18.3 (CH_3_); 24.5 (3-CH_2_); 28.6 (4-CH_2_); 32.5 (2-CH_2_); 105.5 (C-5); 119.9; 127.6 (2C Ph); 128.1 (2C Ph); 128.3 (1C Ph); 137.2; 143.9; 149.6; 156.9; 160.3; 164.9; 173.7; 173.9. HRMS *m*/*z*: calcd for C_19_H_19_N_4_O_4_S^+^[M + H]^+^: 399.1122; found: 399.1136.

4-(5-((6-Methyl-2-oxo-4-(thiophen-2-yl)-1,2-dihydropyridin-3-yl)carbamoyl)-1,3,4-thiadiazol-2-yl)butanoic acid (**7c**). Beige powder, yield 228 mg, 74%. M.p. 197–200 °C. ^1^H NMR (400 MHz, DMSO-*d*_6_) δ ppm 2.0 (p, *J* = 6.6 Hz, 2H, 3-CH_2_); 2.23 (s, 3H, CH_3_); 2.36 (t, *J* = 6.6 Hz, 2H, 2-CH_2_); 3.20 (t, *J* = 6.6 Hz, 2H, 4-CH_2_); 6.48 (s, 1H, H-5); 7.13 (br. d, *J* = 4.9 Hz, 1H, H-4 thiophene); 7.69 (br. s, 2H, H-3,5 thiophene); 10.27 (s, 1H, NHCO’); 11.84 (br. s, 2H, NHCO, COOH). ^13^C NMR (100 MHz, DMSO-*d*_6_) δ ppm 18.5 (CH_3_); 24.7 (3-CH_2_); 28.8 (4-CH_2_); 32.6 (2-CH_2_); 102.7 (C-5); 118.4; 127.2; 129.2; 130.2; 137.1; 141.5; 143.7; 157.8; 160.4; 165.2; 173.9; 174.2. HRMS *m*/*z*: calcd for C_17_H_17_N_4_O_4_S_2_^+^[M + H]^+^: 405.0686; found: 405.0701.

(*E*)-3-(5-((4,6-Dimethyl-2-oxo-1,2-dihydropyridin-3-yl)carbamoyl)-1,3,4-thiadiazol-2-yl)acrylic acid (**8a**). Beige crystals, yield 149 mg, 62%. M.p. 261–263 °C. ^1^H NMR (80 MHz, DMSO-*d*_6_) δ ppm 1.97 (s, 3H, CH_3_); 2.12 (s, 3H, CH_3_); 5.90 (s, 1H, H-5); 6.88 (d, *J* = 15.6, 1H, =CHCOH); 7.80 (d, *J* = 15.6, 1H, 3-CH=); 9.13 (s, 1H, 1-NHCO’); 11.91 (s, 1H, 1-NH); 12.17 (bs, 1H, OH). ^13^C NMR (21 MHz, DMSO-*d*_6_) δ ppm 18.2 (CH_3′_); 18.3 (CH_3_); 106.7 (C-5); 121.3; 126.6 (=CHCOH); 134.7 (3-CH=); 139.8; 142.1; 146.2; 159.3; 158.8; 160.9; 170.8. HRMS *m*/*z*: calcd for C_13_H_13_N_4_O_4_S^+^[M + H]^+^: 321.0652; found: 321.0645.

(*E*)-3-(5-((6-Methyl-2-oxo-4-phenyl-1,2-dihydropyridin-3-yl)carbamoyl)-1,3,4-thiadiazol-2-yl)acrylic acid (**8b**). White crystals, yield 151 mg, 50%. M.p. 199–202 °C. ^1^H NMR (500 MHz, DMSO-*d*_6_) δ ppm 2.21 (s, 3H, CH_3_); 6.03 (s, 1H, H-5); 6.90 (d, 1H, *J* = 16.0, =CHCOH); 7.33–7.38 (m, 5H, H-2,3,4,5 Ph); 7.78 (d, 1H, *J* = 16.0, 1H, 3-CH=); 9.28 (s, 1H, 1-NHCO’); 11.85 (s, 1H, 1-NH); 11.93 (bs, 1H, OH). ^13^C NMR (125 MHz, DMSO-*d*_6_) δ ppm 18.4 (CH_3_); 105.6 (C-5); 120.2; 126.2 (=CHCOH); 127.6 (2C Ph); 128.2 (2C Ph); 128.3 (1C Ph); 135.6 (3-CH=); 137.4; 139.7; 143.5; 148.9; 159.9; 160.5; 160.8; 170.7. HRMS *m*/*z*: calcd for C_18_H_15_N_4_O_4_S^+^[M + H]^+^: 383.0809; found: 383.0831.

(*E*)-3-(5-((6-Methyl-2-oxo-4-(thiophen-2-yl)-1,2-dihydropyridin-3-yl)carbamoyl)-1,3,4-thiadiazol-2-yl)acrylic acid (**8c**). Grey crystals, yield 216 mg, 70%. M.p. 247–250 °C. ^1^H NMR (500 MHz, DMSO-*d*_6_) δ ppm 2.21 (s, 3H, CH_3_); 6.45 (s, 1H, H-5); 6.95 (d, 1H, *J* = 16.0, =CHCOH); 7.13–7.15 (m, 1H, H-4′ Th); 7.65–7.69 (m, 2H, H-3′,5′ Th); 7.79 (d, 1H, *J* = 16.0, 3-CH=); 9.50 (s, 1H, 1-NHCO’); 11.75 (bs, 1H, OH); 11.97 (bs, 1H, 1-NH); ^13^C NMR (125 MHz, DMSO-*d*_6_) δ ppm 18.5 (CH_3_); 102.7 (C-5); 118.6; 126.6 (=CHCOH); 127.1 (1C thiophene); 128.9 (1C thiophene); 129.9 (1C thiophene); 135.4 (3-CH=); 137.2; 139.9; 141.2; 143.3; 160.5; 160.9; 170.7. HRMS *m*/*z*: calcd for C_16_H_13_N_4_O_4_S_2_^+^[M + H]^+^: 389.0373; found: 389.0396.

2-(5-((4,6-Dimethyl-2-oxo-1,2-dihydropyridin-3-yl)carbamoyl)-1,3,4-thiadiazol-2-yl)benzoic acid (**9a**). Gray powder, yield 266 mg, 72%. M.p. 315–318 °C. ^1^H NMR (400 MHz, DMSO-*d*_6_) δ ppm 2.06 (s, 3H, CH_3′_); 2.16 (s, 3H, CH_3_); 5.95 (s, 1H, H-5); 9.64 (s, 1H, NHCO’); 10.08 (s,1H, NHCO); 11.79 (bs., 1H, OH). ^13^C NMR (100 MHz, DMSO-*d*_6_) δ ppm 18.1 (CH_3′_); 18.2 (CH_3_); 106.6 (C-5); 121.2; 124.1 (C Ph); 129.3 (C Ph); 129.9 (C Ph); 131.7 (C Ph); 132.5 (C Ph); 135.7 (C Ph); 142.9; 147.4; 156.3; 159.7; 166.1; 167.7; 170.3. HRMS *m*/*z*: calcd for C_17_H_15_N_4_O_4_S^+^[M + H]^+^: 371.0809; found: 371.0825.

2-(5-((6-Methyl-2-oxo-4-phenyl-1,2-dihydropyridin-3-yl)carbamoyl)-1,3,4-thiadiazol-2-yl)benzoic acid (**9b**). Gray powder, yield 368 mg, 85%. M.p. 176–180 °C. ^1^H NMR (80 MHz, DMSO-*d*_6_) δ ppm 2.41 (s, 3H, CH_3_); 6.08 (s, 1H, H-5); 7.42–7.92 (m, 9H, H-2,3,4,5,6 Ph; H-3,4,5,6 Ph’); 10.18 (s, 1H, NHCO’); 11.99 (br. s, 2H, NHCO, OH). ^13^C NMR (21 MHz, DMSO-*d*_6_) δ ppm 18.4 (CH_3_); 105.6 (C-5); 120.1; 127.7 (2C Ph); 128.3 (3C Ph); 128.6 (2C Ph); 129.9; 131.3; 131.5; 132.8; 137.2; 144.1; 149.8; 157.0; 160.3; 165.9; 167.8; 170.2. HRMS *m*/*z*: calcd for C_22_H_17_N_4_O_4_S^+^[M + H]^+^: 433.0965; found: 433.0970.

2-(5-((6-Methyl-2-oxo-4-(thiophen-2-yl)-1,2-dihydropyridin-3-yl)carbamoyl)-1,3,4-thiadiazol-2-yl)benzoic acid (**9c**). Gray powder, yield 411 mg, 94%. M.p. 275–277 °C. ^1^H NMR (80 MHz, DMSO-*d*_6_) δ ppm 2.24 (s, 3H, CH_3_); 6.50 (s, 1H, H-5); 7.11–7.21 (m, 1H, H-4 thiophene); 7.70–7.89 (m, 6H, H-3,5 thiophene, H-3,4,5,6 Ph); 10.42 (s, 1H, NHCO’); 11.30–12.20 (m, 2H, NHCO, OH). ^13^C NMR (21 MHz, DMSO-*d*_6_) δ ppm 18.5 (CH_3_); 102.7; 118.4; 127.3; 128.6; 129.2; 129.9; 130.1; 131.4; 131.7; 132.5; 137.0; 141.5; 143.8; 157.7; 160.1; 160.4; 166.0; 167.7; 170.3. HRMS *m*/*z*: calcd for C_20_H_15_N_4_O_4_S_2_^+^[M + H]^+^: 439.0529; found: 439.0535.

For subsequent biological tests to obtain water-soluble derivatives, the resulting acids were converted to potassium salts by dissolving in a 2-fold excess of potassium hydroxide.

### 3.2. Biological Tests

*Study of the degree of inhibition of α-glucosidase activity by test compounds [25].*

The principle of the method is as follows: the α-glucosidase enzyme hydrolyzes 4-Nitrophenyl α-D-glucopyranoside to form a colorimetric product (405 nm), the concentration of which is proportional to the *α*-glucosidase activity. To 500 μL of phosphate buffer (0.1 M, pH 6.8), 100 μL of *α*-glucosidase (1 U/mL) and 200 μL of a solution of the test sample (150 μM) were added. The resulting mixture was incubated for 15 min at +37 °C, then 200 μL of a solution of 4-Nitrophenyl α-D-glucopyranoside (p-Nitrophenyl *α*-D-glucopyranoside, P-NPG) (5 mM) was added, and then the mixture was incubated at +37 °C for 20 min. Then, the reaction was stopped by adding 500 μL of sodium carbonate (0.1 M). A solution of α-glucosidase (1 U/mL) was used as a blank. We used 200 μL of DMSO in triplicate as a negative control. Acarbose at a concentration of 150 μM/mL (positive control) was used as a reference drug. At the same time, a negative control was set without addition of test compounds. All samples were studied in triplicate. Inhibitory activity was expressed as a percentage (%) according to the degree of inhibition of α-glucosidase in comparison with the negative control. It was calculated by the following formula:

Inhibitory activity (%) = (1 − As/Ac)∗100%, where As is the optical density of the test compound, and Ac is the optical density of control.

The IC_50_ was determined from the graph of enzyme activity changes depending on the sample concentration.

Statistical processing of the results was carried out using the “Excel 365 v2401” program. The obtained results are presented as “mean ± standard error of the mean”; diabetic activity was assessed by the degree of inhibition of *α*-amylase and *α*-glucosidase activity by the test substances. The *α*-amylase and α-glucosidase enzymes determine the extent to which glucose enters the bloodstream from the gastrointestinal tract. Inhibition of these enzymes can be useful for lowering postprandial glucose levels [25].

## 4. Conclusions

Thus, based on thiogidrazides **4a**–**c** and anhydrides of glutaric, maleic, and phthalic acids, nine new derivatives of 1,3,4-thiadiazole containing various acid linkers were synthesized. Bioassays of the newly synthesized derivatives of 1,3,4-thiadiazole **5′**–**9′a**–**c** with acid linkers showed that they possess pronounced inhibitory activity against α-glucosidase (up to 95.0%), which is 1.9 times higher than the values of the reference drug acarbose (49.5%). One of the compounds (**9′b**) has an IC_50_ value of 3.66 mM, which is almost 3.7 times lower than the reference drug acarbose (IC_50_ = 13.88 mM). The structure–activity relationship of the compounds was also established.

The results of molecular docking correlate well with the screening data. For example, the presence of a butanoic acid linker and a benzoyl fragment in the 1,3,4-thiadiazole cycle of compounds **7b**, **7c**, **9b** increases their binding affinity with the target proteins compared to other derivatives **3**(**a**–**c**), **4**(**a**–**c**), **5**(**a**–**c**), **6**(**a**–**c**). The presence of a phenyl substituent at the fourth position on the pyridone ring in compounds **7b**, **9b** also leads to an increase in binding affinity compared to the methyl and partially thiophenyl substituents. Lipinski’s rule calculations also showed that the synthesized compounds **7b**, **7c**, **9b** satisfy Ro5 and meet all criteria for good permeability and the acceptable oral bioavailability of potential drugs. Furthermore, a comparative analysis of the molecular docking data of synthesized compounds on receptor proteins (3W37, 2QV4, and 5NN8) showed a sufficiently good correlation of α-glucosidase enzyme inhibition with the obtained results (IC_50_).

Based on these findings, the newly synthesized derivatives of 1,3,4-thiadiazole based on 3-aminopyridine-2(1*H*)-ones are promising objects for further investigation of their potential antidiabetic activity.

## Figures and Tables

**Table 1:** Inhibitory activity of tested compounds at a concentration of 15 mM against *α*-glucosidase enzyme and the concentration at which 50% inhibition of *α*-glucosidase activity occurs (IC_50_).

№	Compounds	The Degree of Inhibition of *α*-Glucosidase Activity, %	IC_50_ Value of *α*-Glucosidase Inhibitory Activity (µg/mL)
1.	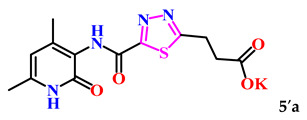	36.3 ± 4.8	16.22 ± 0.76
2.	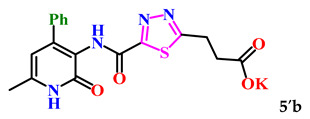	72.4 ± 2.3	10.22 ± 0.82
3.	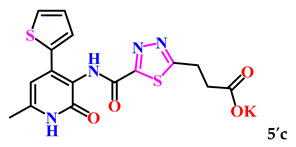	38.3 ± 2.4	18.58 ± 1.20
4.	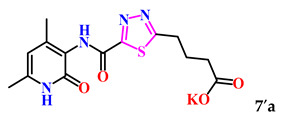	34.8 ± 4.2	20.83 ± 1.06
5.	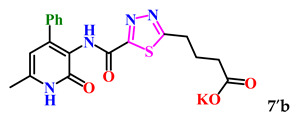	95.0 ± 0.2	6.70 ± 0.78
6.	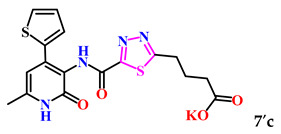	93.9 ± 0.4	8.42 ± 0.29
7.	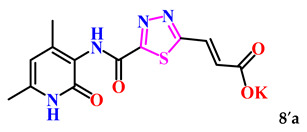	14.0 ± 3.0	17.93 ± 0.28
8.	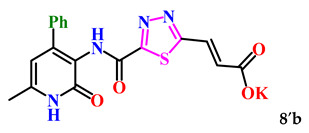	68.2 ± 4.7	10.36 ± 0.60
9.	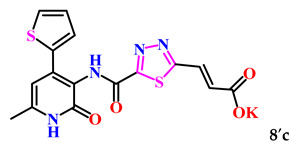	8.0 ± 3.0	48.00±1.67
10.	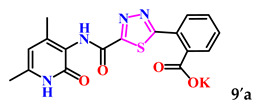	33.6 ± 6.1	14.02 ± 0.49
11.	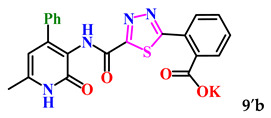	95.2 ± 0.1	3.66 ± 0.05
12.	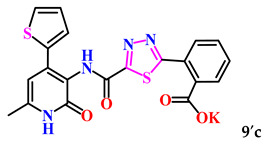	21.0 ± 2.2	24.51 ± 1.54
13.	Acarbose	49.5 ± 1.8	13.88 ± 0.81

**Table 2:** Binding affinity (kcal/mol) for complexes of proteins 3W37, 2QV4, and 5NN8 with native ligands and compounds **7b**, **7c**, **9b**.

	Receptor	3W37	2QV4	5NN8
Ligand				
Acarbose		−8.3	−8.5	−8.0
**5a**		−7.1	−7.2	−6.8
**5b**		−8.6	−8.2	−7.8
**5c**		−8.0	−7.6	−7.2
**7a**		−7.8	−7.6	−6.9
**7b**		−8.5	−7.8	−7.8
**7c**		−7.8	−7.6	−7.0
**8a**		−7.6	−7.9	−7.3
**8b**		−8.9	−8.5	−8.0
**8c**		−8.0	−7.8	−7.6
**9a**		−8.1	−8.8	−7.8
**9b**		−9.0	−9.1	−8.8
**9c**		−8.5	−8.9	−7.4

**Table 3:** Basic amino acid interactions and H-bonds.

Compound	Receptor	H-Bond	Residual Amino Acid Interactions	
			Pi-Sulfur/Pi-Anion/Pi-Pi Stacked/Pi-Pi T-Shaped/Pi-Alkyl/Amide-Pi Stacked/Pis Interactions	Van-der Walls Interactions
**9b**	5NN8	ARG600, ASP518, ASP616	ASP282, PHE525, TRP376, LEU405, TRP481, ILE441, MET519, PHE649,	LEU677, SER676, ASP404, ALA555, SER523, ASN524, LEU650
	2QV4	-	LEU162, TYR62, LEU165, TRP59, HIS201, LYS200, ILE235, ASP197	VAL234, ALA198, TRP58, GLU233, ARG195, HIS101
	3W37	ASP232, ARG552	TRP432, PHE601, PHE476, LYS506, MET470, ASP568, ASP232, ILE358	ASP469, TRP565, SER474, ASN475, TRP329, ASP357.

**Table 4:** Calculated values of compounds **7b**, **7c**, **9b** according to Lipinski’s five rule.

Ligand	Molecular Weight	miLogP	nHBA	nHBD	nViolation
**7b**	398.44	0.130	6	3	0
**7c**	404.46	1.646	8	3	0
**9b**	432.45	3.150	6	3	0

## Data Availability

Data is contained within the article.

## Supplementary Materials

The following supporting information can be downloaded at: https://www.mdpi.com/article/10.3390/ph17030377/s1, Table S1. Complexes between synthesized derivatives 5(**a**–**c**), 7–9(**a**–**c**) and active sites of proteins (PDB: 5NN8, 3W37, 2QV4); Table S2. Basic amino acid interactions and H-bonds.
