# Supplementary material for: Synthesis, Computational Study, and In Vitro α-Glucosidase Inhibitory Action of 1,3,4-Thiadiazole Derivatives of 3-Aminopyridin-2(1H)-ones
Source: Pharmaceuticals (Basel). 2024 Mar 15;17(3):377. doi: 10.3390/ph17030377 (PMC10974052; doi:10.3390/ph17030377)
Supplement: Supplementary file 1 [file pharmaceuticals-17-00377-s001.zip › pharmaceuticals-2816382-supplementary.pdf]

Supporting Information

Synthesis, Computational Study and in vitro α-glucosidase inhibitory action of 1,3,4-thiadiazole derivatives from 3-aminopyridin-2(1*H*)-ones

Zarina Shulgau[1,2*], Irina Palamarchuk[3], Shynggys Sergazy[1,2], Assel Urazbayeva[1,2], Yerlan Ramankulov[4], Ivan Kulakov[2,3]**

[1] National Laboratory Astana, Nazarbayev University, Kabanbai Batyr ave. 53, Z05H0P9 Astana, Kazakhstan
[2] National Center for Biotechnology, 13/5 Kurgalzhynskoe Road, Z05K8D5 Astana, Kazakhstan
[3] Institute of Chemistry, Tyumen State University, 15a Perekopskaya St., Tyumen 625003, Russia; i.v.kulakov@utmn.ru
[4] Nazarbayev University, Kabanbai Batyr ave. 53, Z05H0P9 Astana, Kazakhstan
* Correspondence: zarina.shulgau@icloud.com

Table of Contents

Experimental Procedures 1
1. Materials and Methods........ 1
2.3 Synthesis of 1,3,4-thiadiazoles derivatives **7a-c**, **8a-c**, **9a-c** 2
3. Author Contributions........ 5
4. Copies of NMR Spectra of Products........ 6
5. Copies of MS Spectra of Products........ 15
6. Table S1. Complexes between synthesized derivatives **5(a-c), 7-9(a-c)** and active sites of proteins (PDB: 5NN8, 3W37, 2QV4)........ 18
7. Table S2. Basic amino acid interactions and H-bonds........ 29

Experimental Procedures

1. Materials and Methods

^{1}H and ^{13}C NMR spectra were recorded on a Bruker DRX400 (400 and 100 MHz, respectively) and Bruker AVANCE 500 (500 and 125 MHz, respectively) instruments using DMSO-d_6 the internal standard was TMS or residual solvent signals (2.49 and 39.9 ppm ^{1}H and for ^{13}C nuclei in DMSO-d_6).

Sample were analyzed by HPLC-MS on an Agilent 1260 Infinity II chromatograph coupled to an Agilent 6545 LC/Q-TOF high-resolution mass spectrometer with a Dual AJS ESI ionization source operating in positive ion mode using the following parameters: capillary voltage: 4000 V; spray pressure: 20 (psi); drying gas: 10 *l*/min; gas temperature: 325ºC; sheathed gas flow: 12 *l*/min; shielding gas temperature: 400°C; nozzle voltage: 0 V, fragmentation voltage: 180 V; skimmer voltage: 45 V; octopole RF: 750 V. Mass spectra with LC/MS accuracy were recorded in the range 100-1000 m/z, scan rate 1.5 spectrum/s.
Chromatographic separation was carried out on columns: ZORBAX RRHD Eclipse Plus C18 (2.1 x 50 mm, particle size 1.8 μm). The column temperature during the analysis was maintained at 35°C. The mobile phase was formed by eluents A and B. In the positive ionization mode, 0.1% formic acid solution in deionized water was used as eluent A, and 0.1% formic acid solution in acetonitrile was used as eluent B. Chromatographic separation was performed with elution according to the following scheme: 0-10 min 95% A, 10-13 min 100% B, 13-15 min 95% A. The flow of the mobile phase was maintained at 400 μL/min throughout the analysis. In all experiments, the sample injection volume was 1 μL. The sample was prepared by dissolving the entire sample (in 1000 μL) in methanol (for HPLC). Sample dilution was carried out immediately before analysis.
The recorded data were processed using Agilent MassHunter 10.0 software.
Melting points were determined using a Stuart SMP10 hot bench. Monitoring of the reaction course and the purity of the products was carried out by TLC on Sorbfil plates and visualized using iodine vapor or UV light.

Synthesis of compounds N-(4,6-dimethyl-2-oxo-1,2-dihydropyridin-3-yl)-2-hydrazinyl-2-thioxoacetamide (4a), 2-Hydrazinyl-N-(6-methyl-2- oxo-4-phenyl-1,2-dihydropyridin-3-yl)-2-thioxoacetamide (4b), 2-Hydrazinyl-N-(6-methyl-2-oxo-4-(thiophen-2-yl)-1 ,2-dihydropyridin-3-yl)-2-thioxoacetamide (4c), 3-(5-((4,6-dimethyl-2-oxo-1,2-dihydropyridin-3-yl)carbamoyl)-1,3,4-thiadiazol-2-yl)propanoic (5a), 3-(5-((6-methyl-2-oxo-4-phenyl-1,2-dihydropyridin-3-yl)carbamoyl)-1,3,4-thiadiazol-2-yl)propanoic acid (5b) and 3-(5-((6-methyl-2-oxo-4-(thiophen-2-yl)-1,2-dihydropyridin-3-yl)carbamoyl)-1,3,4-thiadiazol-2-yl)propanoic acid (5c) acid is given in the article [24]

Synthesis of thiadiazoles derivatives 7-9a-c

To (1 mmol) of oxamic acid thiohydrazide **4a-c** in 3 ml of acetic acid was added (3.0 mmol) of the corresponding anhydride (glutaric acid, phthalic acid and maleic acid). The reaction mixture was heated at reflux temperature on vigorous stirring over 5 h, cooled down and poured into water (25 mL). The resulting precipitates were filtered off and dried in air to obtain compounds **7-9a-c** in the indicated yields.

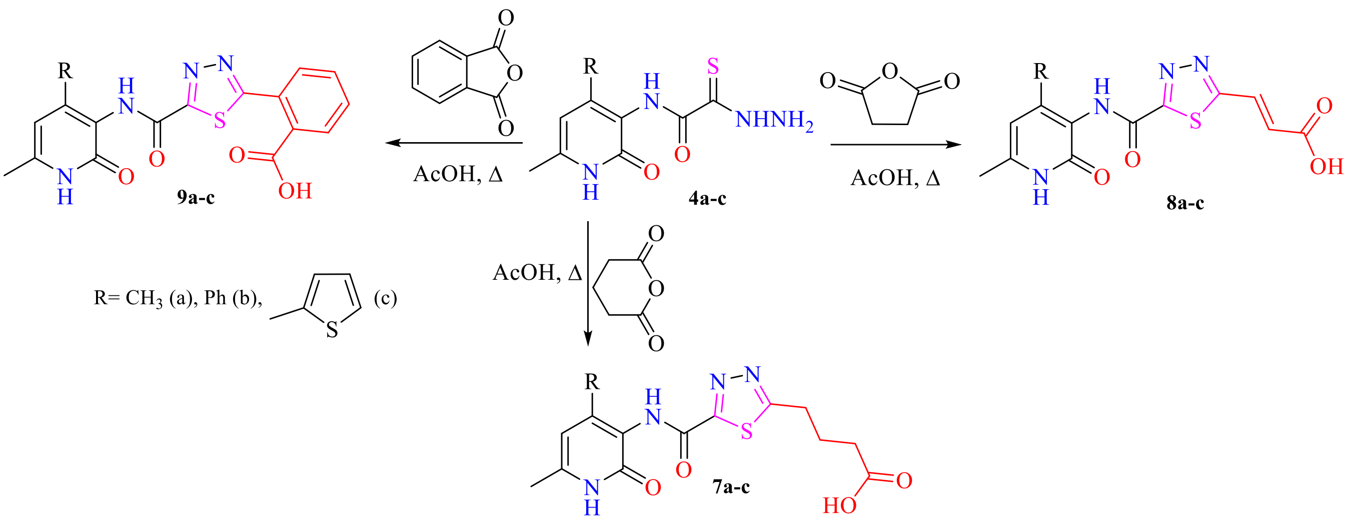

Characterization data of products 7-9a-c

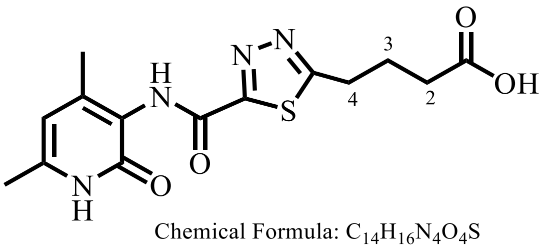 Chemical Formula: $C_{14}H_{16}N_4O_4S$<br>Molecular Weight: 336,37	**4-(5-((4,6-dimethyl-2-oxo-1,2-dihydropyridin-3-yl)carbamoyl)-1,3,4-thiadiazol-2-yl)butanoic acid (7a).**<br>Beige powder, yield 192 mg, 80%. M.p. 248-250°C.<br>^{1}H NMR (400 MHz, DMSO-*d*$_6$) δ ppm 1.98 (p, *J*=7.3 Hz, 2H, 3-CH$_2$); 2.02 (s, 3H, CH$_3$); 2.14 (s, 3H, CH$_3$); 2.35 (t, *J*=7.1 Hz, 2H, 2-CH$_2$); 3.19 (t, *J*=7.1 Hz, 2H, 4-CH$_2$); 5.93 (s, 1H, H-5); 9.92 (s, 1H, NHCO'); 11.79 (br. s, 2H, NHCO, COOH).<br>^{13}C NMR (100 MHz, DMSO-*d*$_6$) δ ppm 18.1 (CH$_3$'); 18.2 (CH$_3$); 24.7 (3-CH$_2$); 28.8 (4-CH$_2$); 32.6 (2-CH$_2$); 106.6 (C-5); 121.3; 142.6; 147.3; 156.4; 159.8; 165.23; 173.7; 174.1.<br>HRMS m/z: calcd for $C_{14}H_{17}N_4O_4S^+$[M+H]$^+$: 337.0965;

	found: 337.0998
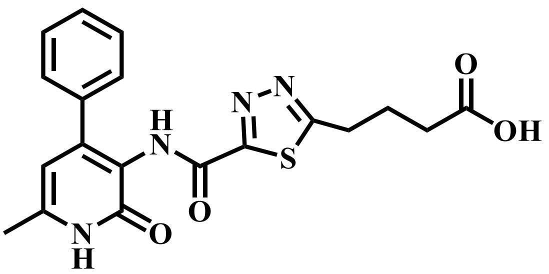<br>Chemical Formula: $C_{19}H_{18}N_4O_4S$<br>Molecular Weight: 398,4370	**4-(5-((6-Methyl-2-oxo-4-phenyl-1,2-dihydropyridin-3-yl)carbamoyl)-1,3,4-thiadiazol-2-yl)butanoic acid (7b).**<br>Beige powder, yield 218 mg, 72%. M.p. 243-246°C.<br>^{1}H NMR (400 MHz, DMSO-*d*$_6$) δ ppm 1.96 (p, *J*=7.6 Hz, 2H, 3-CH_2); 2.24 (s, 3H, CH_3); 2.34 (t, *J*=7.1 Hz, 2H, 2-CH_2); 3.16 (t, *J*=7.6 Hz, 2H, 4-CH_2); 6.08 (s, 1H, H-5); 7.34-7.39 (m, 3H, H-3,4,5 Ph); 7.46 (d, *J*=7.6 Hz, 2H, H-2,6 Ph); 10.07 (s, 1H, NHCO'); 12.05 (br. s, 2H, NHCO, COOH).<br>^{13}C NMR (100 MHz, DMSO-*d*$_6$) δ ppm 18.3 (CH_3); 24.5 (3-CH_2); 28.6 (4-CH_2); 32.5 (2-CH_2); 105.5 (C-5); 119.9; 127.6 (2C Ph); 128.1 (2C Ph); 128.3 (1C Ph); 137.2; 143.9; 149.6; 156.9; 160.3; 164.9; 173.7; 173.9.<br>HRMS m/z: calcd for $C_{19}H_{19}N_4O_4S^+$[M+H]$^+$: 399.1122; found: 399.1136
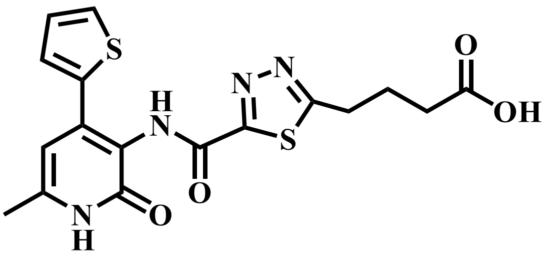<br>Chemical Formula: $C_{17}H_{16}N_4O_4S_2$<br>Molecular Weight: 404,46	**4-(5-((6-Methyl-2-oxo-4-(thiophen-2-yl)-1,2-dihydropyridin-3-yl)carbamoyl)-1,3,4-thiadiazol-2-yl)butanoic acid (7c)**<br>Beige powder, yield 228 mg, 74%. M.p. 197-200°C.<br>^{1}H NMR (400 MHz, DMSO-*d*$_6$) δ ppm 2.0 (p, *J*=6.6 Hz, 2H, 3-CH_2); 2.23 (s, 3H, CH_3); 2.36 (t, *J*=6.6 Hz, 2H, 2-CH_2); 3.20 (t, *J*=6.6 Hz, 2H, 4-CH_2); 6.48 (s, 1H, H-5); 7.13 (br. d, *J*=4.9 Hz, 1H, H-4 thiophene); 7.69 (br. s, 2H, H-3,5 thiophene); 10.27 (s, 1H, NHCO'); 11.84 (br. s, 2H, NHCO, COOH).<br>^{13}C NMR (100 MHz, DMSO-*d*$_6$) δ ppm 18.5 (CH_3); 24.7 (3-CH_2); 28.8 (4-CH_2); 32.6 (2-CH_2); 102.7 (C-5); 118.4; 127.2; 129.2; 130.2; 137.1; 141.5; 143.7; 157.8; 160.4; 165.2; 173.9; 174.2.<br>HRMS m/z: calcd for $C_{17}H_{17}N_4O_4S_2^+$[M+H]$^+$: 405.0686; found: 405.0701.
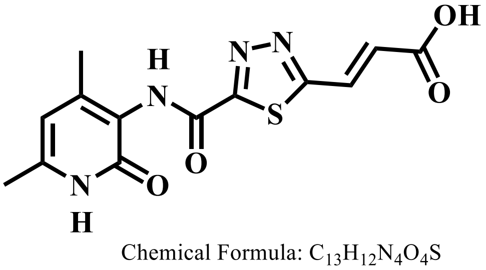<br>Chemical Formula: $C_{13}H_{12}N_4O_4S$<br>Molecular Weight: 320,3230	**(E)-3-(5-((4,6-dimethyl-2-oxo-1,2-dihydropyridin-3-yl)carbamoyl)-1,3,4-thiadiazol-2-yl)acrylic acid (8a).**<br>Beige crystals, yield 149 mg, 62%. M.p. 261-263°C<br>^{1}H NMR (80 MHz, DMSO-*d*$_6$) δ ppm 1.97 (s, 3H, CH_3); 2.12 (s, 3H, CH_3); 5.90 (s, 1H, H-5); 6.88 (d, *J*=15.6, 1H, =CHCOH); 7.80 (d, *J*=15.6, 1H, 3-CH=); 9.13 (s, 1H, 1-NHCO'); 11.91 (s, 1H, 1-NH); 12.17 (bs, 1H, OH). ^{13}C NMR (21 MHz, DMSO-*d*$_6$) δ ppm 18.2 (CH_3'); 18.3 (CH_3); 106.7 (C-5); 121.3; 126.6 (=CHCOH); 134.7 (3-CH=); 139.8; 142.1; 146.2; 159.3; 158.8; 160.9; 170.8.<br>HRMS m/z: calcd for $C_{13}H_{13}N_4O_4S^+$[M+H]$^+$: 321.0652; found: 321.0645

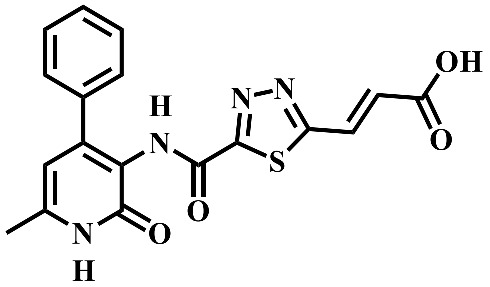<br>Chemical Formula: $C_{18}H_{14}N_4O_4S$<br>Molecular Weight: 382,3940	**(E)-3-(5-((6-methyl-2-oxo-4-phenyl-1,2-dihydropyridin-3-yl)carbamoyl)-1,3,4-thiadiazol-2-yl)acrylic acid (8b).**<br>White crystals, yield 151 mg, 50%. M.p. 199-202°C<br>^{1}H NMR (500 MHz, DMSO-*d*$_6$) δ ppm 2.21 (s, 3H, CH_3); 6.03 (s, 1H, H-5); 6.90 (d, 1H, *J*=16.0, =CHCOH); 7.33-7.38 (m, 5H, H-2,3,4,5 Ph); 7.78 (d, 1H, *J*=16.0, 1H, 3-CH=); 9.28 (s, 1H, 1-NHCO'); 11.85 (s, 1H, 1-NH); 11.93 (bs, 1H, OH). ^{13}C NMR (125 MHz, DMSO-*d*$_6$) δ ppm 18.4 (CH_3); 105.6 (C-5); 120.2; 126.2 (=CHCOH); 127.6 (2C Ph); 128.2 (2C Ph); 128.3 (1C Ph); 135.6 (3-CH=); 137.4; 139.7; 143.5; 148.9; 159.9; 160.5; 160.8; 170.7.<br>HRMS m/z: calcd for $C_{18}H_{15}N_4O_4S^+$[M+H]$^+$: 383.0809; found: 383.0831.
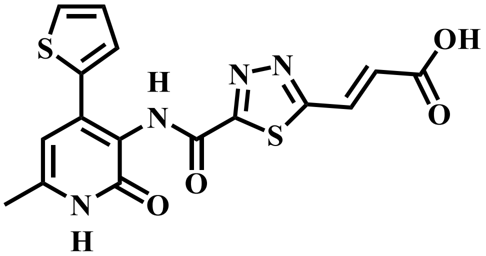<br>Chemical Formula: $C_{16}H_{12}N_4O_4S_2$<br>Molecular Weight: 388,4160	**(E)-3-(5-((6-methyl-2-oxo-4-(thiophen-2-yl)-1,2-dihydropyridin-3-yl)carbamoyl)-1,3,4-thiadiazol-2-yl)acrylic acid (8c).**<br>Grey crystals, yield 216 mg, 70%. M.p. 247-250ºC<br>^{1}H NMR (500 MHz, DMSO-*d*$_6$) δ ppm 2.21 (s, 3H, CH_3); 6.45 (s, 1H, H-5); 6.95 (d, 1H, *J*=16.0, =CHCOH); 7.13-7.15 (m, 1H, H-4' Th); 7.65-7.69 (m, 2H, H-3',5' Th); 7.79 (d, 1H, *J*=16.0, 3-CH=); 9.50 (s, 1H, 1-NHCO'); 11.75 (bs, 1H, OH); 11.97 (bs, 1H, 1-NH); ^{13}C NMR (125 MHz, DMSO-*d*$_6$) δ ppm 18.5 (CH_3); 102.7 (C-5); 118.6; 126.6 (=CHCOH); 127.1 (1C Th); 128.9 (1C Th); 129.9 (1C Th); 135.4 (3-CH=); 137.2; 139.9; 141.2; 143.3; 160.5; 160.9; 170.7.<br>HRMS m/z: calcd for $C_{16}H_{13}N_4O_4S_2^+$[M+H]$^+$: 389.0373; found: 389.0396.
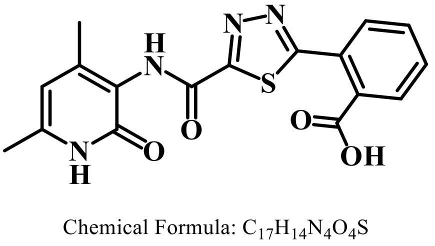<br>Chemical Formula: $C_{17}H_{14}N_4O_4S$<br>Molecular Weight: 370,38	**2-(5-((4,6-dimethyl-2-oxo-1,2-dihydropyridin-3-yl)carbamoyl)-1,3,4-thiadiazol-2-yl)benzoic acid (9a).**<br>Gray powder, yield 266 mg, 72%. M.p. 315-318ºC.<br>^{1}H NMR (400 MHz, DMSO-*d*$_6$) δ ppm 2.06 (s, 3H, CH_3'); 2.16 (s, 3H, CH_3); 5.95 (s, 1H, H-5); 9.64 (s, 1H, NHCO'); 10.08 (s, 1H, NHCO); 11.79 (bs, 1H, OH).<br>^{13}C NMR (100 MHz, DMSO-*d*$_6$) δ ppm 18.1 (CH_3'); 18.2 (CH_3); 106.6 (C-5); 121.2; 124.1 (C Ph); 129.3 (C Ph); 129.9 (C Ph); 131.7 (C Ph); 132.5 (C Ph); 135.7 (C Ph); 142.9; 147.4; 156.3; 159.7; 166.1; 167.7; 170.3.<br>HRMS m/z: calcd for $C_{17}H_{15}N_4O_4S^+$[M+H]$^+$: 371.0809; found: 371.0825

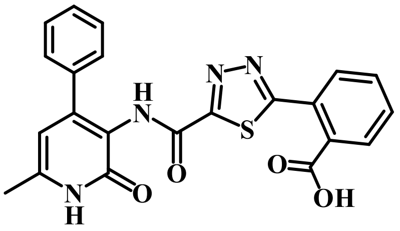<br>Chemical Formula: $C_{22}H_{16}N_4O_4S$<br>Molecular Weight: 432,45	**2-(5-((6-methyl-2-oxo-4-phenyl-1,2-dihydropyridin-3-yl)carbamoyl)-1,3,4-thiadiazol-2-yl)benzoic acid (9b)**<br>Gray powder, yield 368 mg, 85%. M.p. 176-180 °C.<br>^{1}H NMR (80 MHz, DMSO-*d*$_6$) δ ppm 2.41 (s, 3H, CH_3); 6.08 (s, 1H, H-5); 7.42-7.92 (m, 9H, H-2,3,4,5,6 Ph; H-3,4,5,6 Ph'); 10.18 (s, 1H, NHCO'); 11.99 (br. s, 2H, NHCO, OH).<br>^{13}C NMR (21 MHz, DMSO- *d*$_6$) δ ppm 18.4 (CH_3); 105.6 (C-5); 120.1; 127.7 (2C Ph); 128.3 (3C Ph); 128.6 (2C Ph); 129.9; 131.3; 131.5; 132.8; 137.2; 144.1; 149.8; 157.0; 160.3; 165.9; 167.8; 170.2.<br>HRMS m/z: calcd for $C_{22}H_{17}N_4O_4S^+$[M+H]$^+$: 433.0965; found: 433.0970.
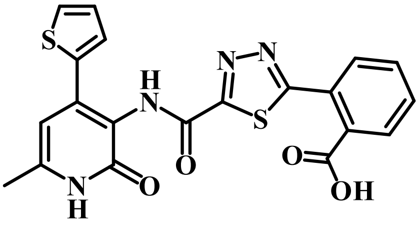<br>Chemical Formula: $C_{20}H_{14}N_4O_4S_2$<br>Molecular Weight: 438,48	**2-(5-((6-methyl-2-oxo-4-(thiophen-2-yl)-1,2-dihydropyridin-3-yl)carbamoyl)-1,3,4-thiadiazol-2-yl)benzoic acid (9c)**<br>Gray powder, yield 411 mg, 94%. M.p. 275-277°C.<br>^{1}H NMR (80 MHz, DMSO- *d*$_6$) δ ppm 2.24 (s, 3H, CH_3); 6.50 (s, 1H, H-5); 7.11-7.21 (m, 1H, H-4 thiophene); 7.70-7.89 (m, 6H, H-3,5 thiophene, H-3,4,5,6 Ph); 10.42 (s, 1H, NHCO'); 11.30-12.20 (m, 2H, NHCO, OH).<br>^{13}C NMR (21 MHz, DMSO-*d*$_6$) δ ppm 18.5 (CH_3); 102.7; 118.4; 127.3; 128.6; 129.2; 129.9; 130.1; 131.4; 131.7; 132.5; 137.0; 141.5; 143.8; 157.7; 160.1; 160.4; 166.0; 167.7; 170.3.<br>HRMS m/z: calcd for $C_{20}H_{15}N_4O_4S_2^+$[M+H]$^+$: 439.0529; found: 439.0535.

Author Contributions

Spectrophotometric studies were performed on the basis of the Research Resource Center "Natural Resource Management and Physico-Chemical Research" Institute of Chemistry, Tyumen State University.

Copies of NMR Spectra of Products

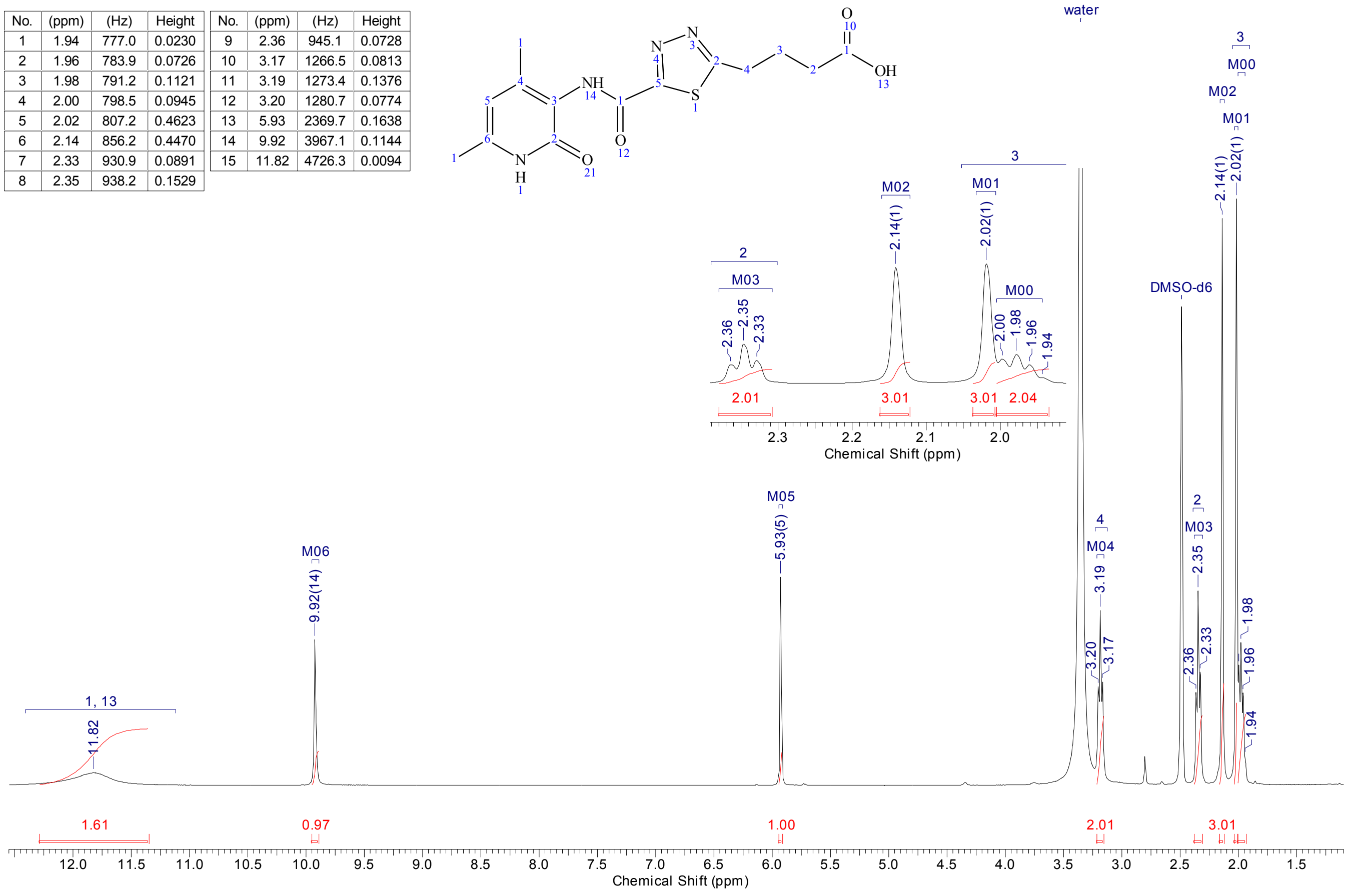

No.	(ppm)	(Hz)	Height	No.	(ppm)	(Hz)	Height
1	1.94	777.0	0.0230	9	2.36	945.1	0.0728
2	1.96	783.9	0.0726	10	3.17	1266.5	0.0813
3	1.98	791.2	0.1121	11	3.19	1273.4	0.1376
4	2.00	798.5	0.0945	12	3.20	1280.7	0.0774
5	2.02	807.2	0.4623	13	5.93	2369.7	0.1638
6	2.14	856.2	0.4470	14	9.92	3967.1	0.1144
7	2.33	930.9	0.0891	15	11.82	4726.3	0.0094
8	2.35	938.2	0.1529				

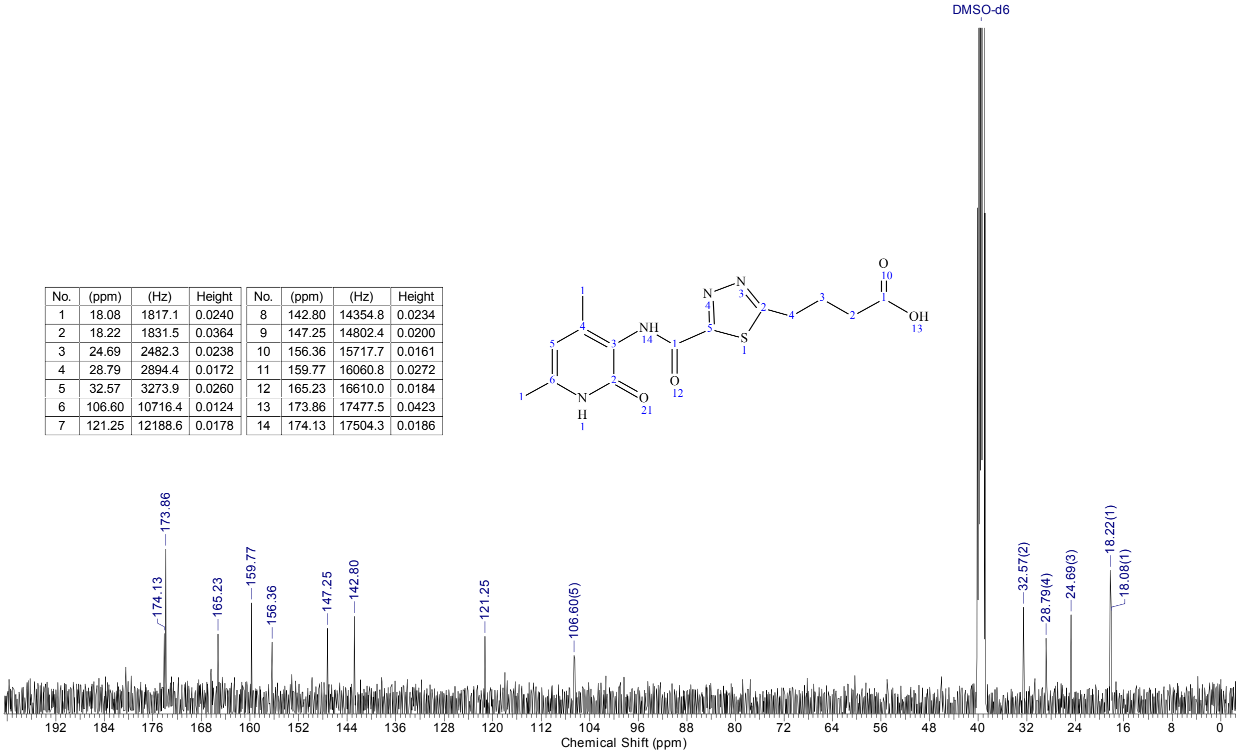

No.	(ppm)	(Hz)	Height	No.	(ppm)	(Hz)	Height
1	18.08	1817.1	0.0240	8	142.80	14354.8	0.0234
2	18.22	1831.5	0.0364	9	147.25	14802.4	0.0200
3	24.69	2482.3	0.0238	10	156.36	15717.7	0.0161
4	28.79	2894.4	0.0172	11	159.77	16060.8	0.0272
5	32.57	3273.9	0.0260	12	165.23	16610.0	0.0184
6	106.60	10716.4	0.0124	13	173.86	17477.5	0.0423
7	121.25	12188.6	0.0178	14	174.13	17504.3	0.0186

^{1}H (500 MHz, DMSO-d_6) and ^{13}C (125 MHz, DMSO-d_6) NMR Spectra of **7a**

No.	(ppm)	(Hz)	Height	No.	(ppm)	(Hz)	Height	No.	(ppm)	(Hz)	Height
1	1.93	965.5	0.0331	9	2.35	1177.0	0.1210	17	7.38	3691.1	0.2105
2	1.95	972.8	0.1080	10	3.14	1570.6	0.1135	18	7.39	3697.9	0.0961
3	1.96	980.1	0.1574	11	3.16	1578.4	0.1877	19	7.45	3727.7	0.2402
4	1.98	987.9	0.1134	12	3.17	1585.8	0.1069	20	7.47	3735.0	0.1687
5	1.99	995.2	0.0361	13	6.08	3039.2	0.2550	21	10.07	5036.0	0.1445
6	2.24	1122.0	0.6559	14	7.34	3669.1	0.0946	22	12.05	6024.4	0.0176
7	2.32	1162.8	0.1419	15	7.35	3676.4	0.1110				
8	2.34	1169.6	0.2547	16	7.37	3683.7	0.1471				

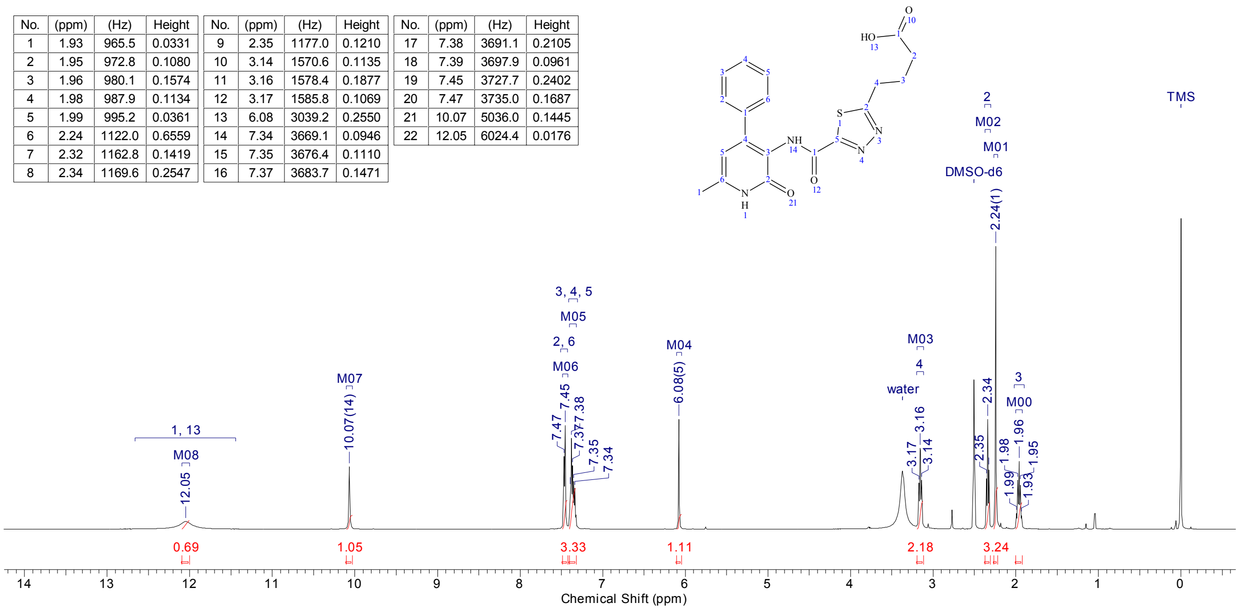

No.	(ppm)	(Hz)	Height	No.	(ppm)	(Hz)	Height
1	18.33	2305.2	0.0790	10	137.18	17251.1	0.0509
2	24.51	3081.8	0.0932	11	143.96	18104.0	0.0301
3	28.64	3602.3	0.0668	12	149.62	18816.1	0.0287
4	32.48	4084.6	0.1007	13	156.96	19738.9	0.0321
5	105.48	13264.7	0.0494	14	160.28	20156.7	0.0426
6	119.97	15086.7	0.0301	15	164.91	20739.0	0.0348
7	127.61	16047.6	0.1278	16	173.71	21845.3	0.0735
8	128.13	16114.0	0.1609	17	173.91	21870.7	0.0316
9	128.31	16135.8	0.0658				

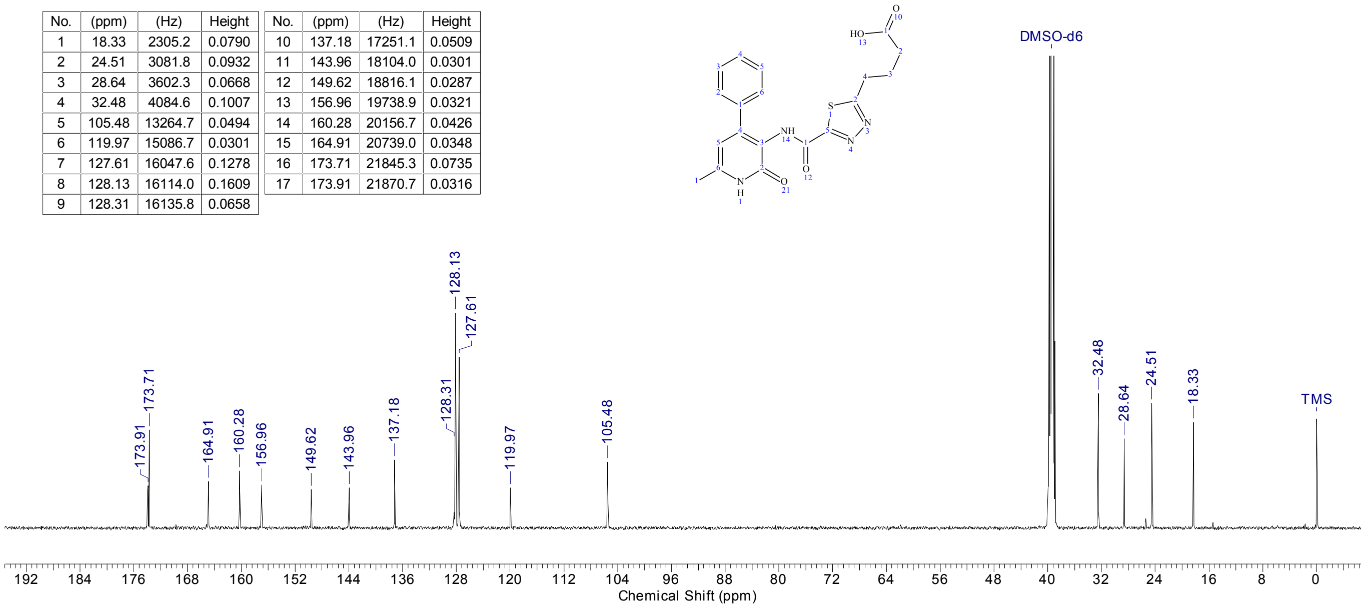

^{1}H (500 MHz, DMSO-d_6) and ^{13}C (125 MHz, DMSO-d_6) NMR Spectra of **7b**

No.	(ppm)	(Hz)	Height
1	1.96	782.5	0.0154
2	1.98	791.7	0.0494
3	2.00	798.1	0.0701
4	2.01	804.9	0.0525
5	2.04	813.6	0.0168
6	2.23	890.6	0.3179
7	2.35	938.7	0.0626
8	2.36	945.1	0.0987
9	2.38	951.9	0.0534
10	3.19	1273.4	0.0628
11	3.20	1279.8	0.0952
12	3.22	1286.7	0.0596
13	6.48	2591.4	0.1169
14	7.13	2850.6	0.0699
15	7.14	2854.3	0.0738
16	7.69	3074.1	0.1614
17	10.27	4105.8	0.0751
18	11.84	4732.8	0.0098

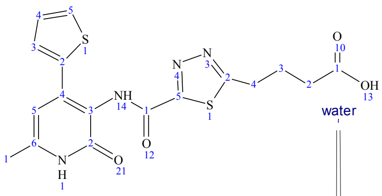
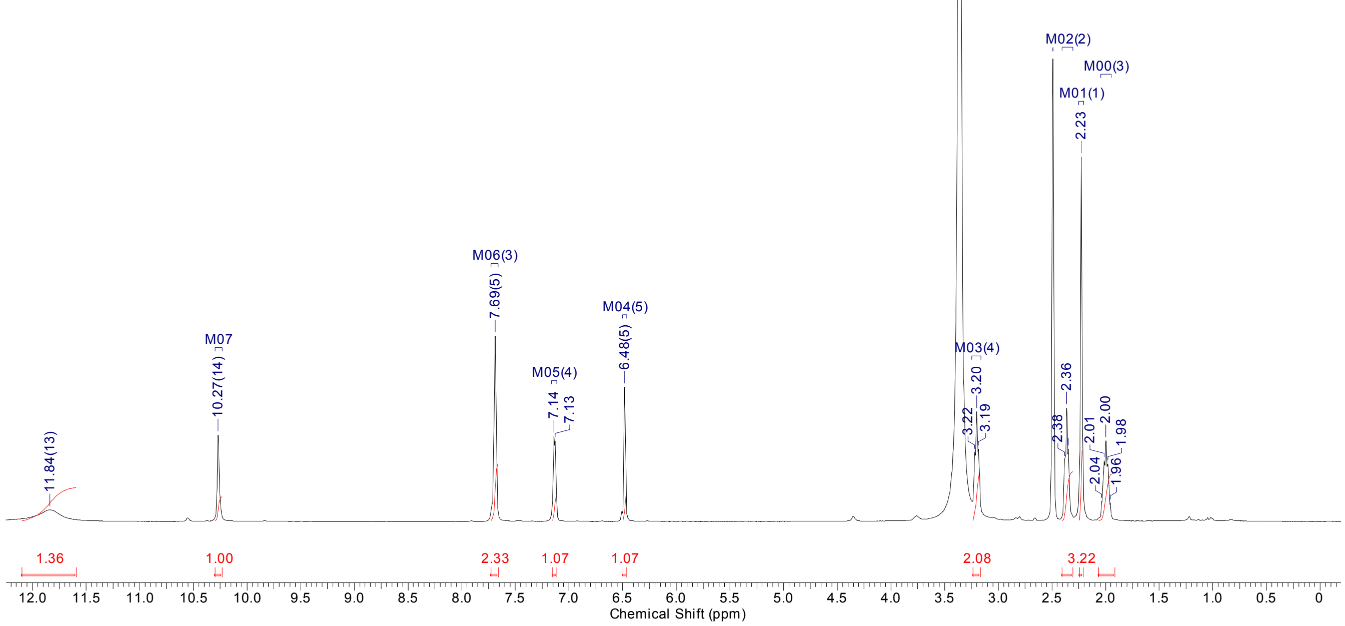

No.	(ppm)	(Hz)	Height
1	18.51	1861.2	0.0218
2	24.65	2478.4	0.0185
3	28.82	2897.3	0.0111
4	32.64	3280.7	0.0160
5	102.70	10323.5	0.0115
6	118.41	11903.0	0.0117
7	127.23	12789.6	0.0098
8	129.16	12984.2	0.0160
9	130.19	13087.7	0.0144
10	137.05	13776.8	0.0183
11	141.47	14221.6	0.0145
12	143.74	14449.7	0.0139
13	157.82	15865.3	0.0142
14	160.41	16125.1	0.0136
15	165.18	16604.3	0.0123
16	173.87	17478.4	0.0217
17	174.19	17510.0	0.0160

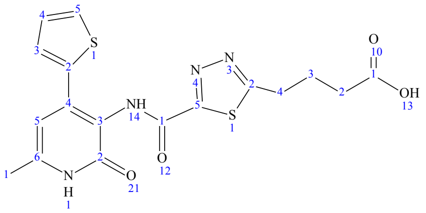
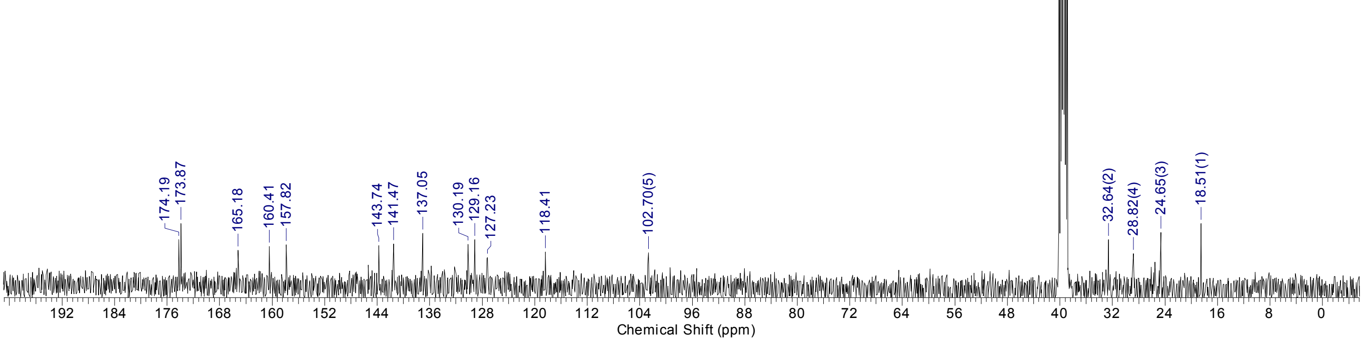

^{1}H (500 MHz, DMSO-d_6) and ^{13}C (125 MHz, DMSO-d_6) NMR Spectra of **7c**

No.	(ppm)	(Hz)	Height	No.	(ppm)	(Hz)	Height
1	1.97	158.5	1.0000	6	7.70	620.1	0.3626
2	2.12	171.0	0.8684	7	7.90	635.7	0.2782
3	5.90	475.0	0.3375	8	9.13	734.6	0.3009
4	6.79	546.4	0.2824	9	11.91	958.6	0.3475
5	6.98	561.8	0.3661	10	12.17	979.6	0.0722

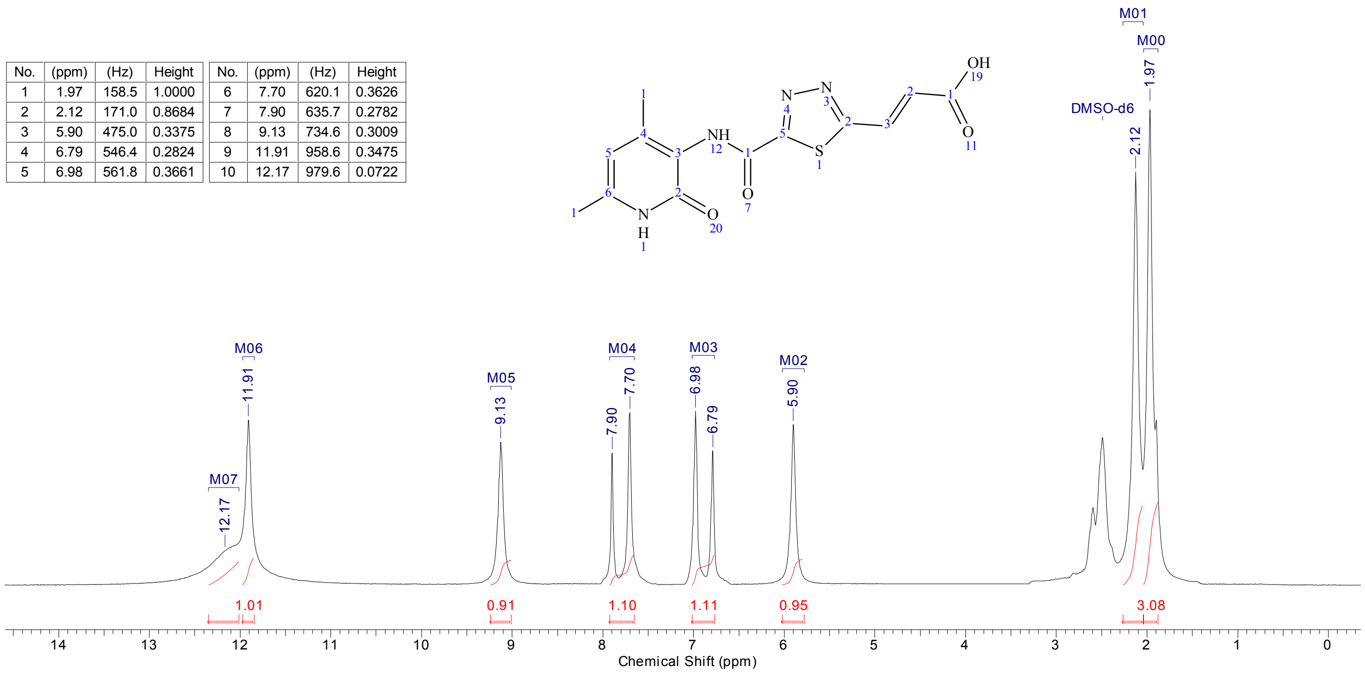

No.	(ppm)	(Hz)	Height	No.	(ppm)	(Hz)	Height
1	18.16	367.6	-0.3735	8	142.06	2875.9	0.4425
2	18.33	371.0	-0.3894	9	146.20	2959.8	0.2839
3	106.71	2160.3	-0.2545	10	159.28	3224.5	0.2049
4	121.27	2455.1	0.2555	11	159.83	3235.7	0.3781
5	126.58	2562.5	-0.2258	12	160.97	3258.7	0.4057
6	134.66	2726.0	-0.2240	13	170.75	3456.7	0.3965
7	139.85	2831.2	0.2605				

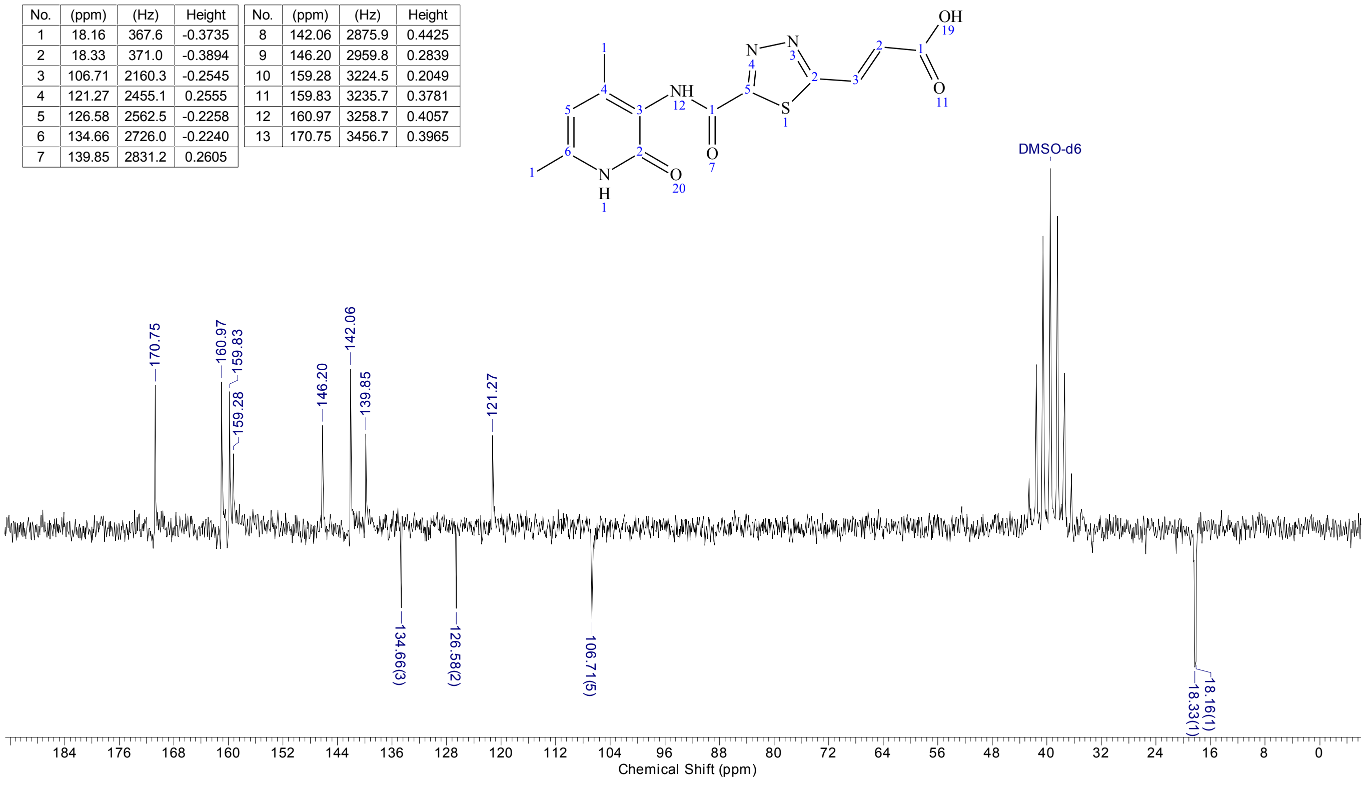

^{1}H (80 MHz, DMSO-d_6) and ^{13}C (21 MHz, DMSO-d_6) NMR Spectra of **8a**

No.	(ppm)	(Hz)	Height	No.	(ppm)	(Hz)	Height
1	2.21	1103.9	0.6998	9	7.38	3691.2	0.3732
2	6.03	3016.5	0.2421	10	7.39	3698.1	0.0700
3	6.88	3443.1	0.1332	11	7.76	3879.4	0.1702
4	6.92	3459.1	0.1703	12	7.79	3895.4	0.1292
5	7.34	3670.6	0.0803	13	9.28	4639.7	0.1725
6	7.34	3673.4	0.1121	14	11.85	5924.7	0.1414
7	7.35	3676.6	0.1234	15	11.93	5965.5	0.0167
8	7.37	3684.8	0.2619				

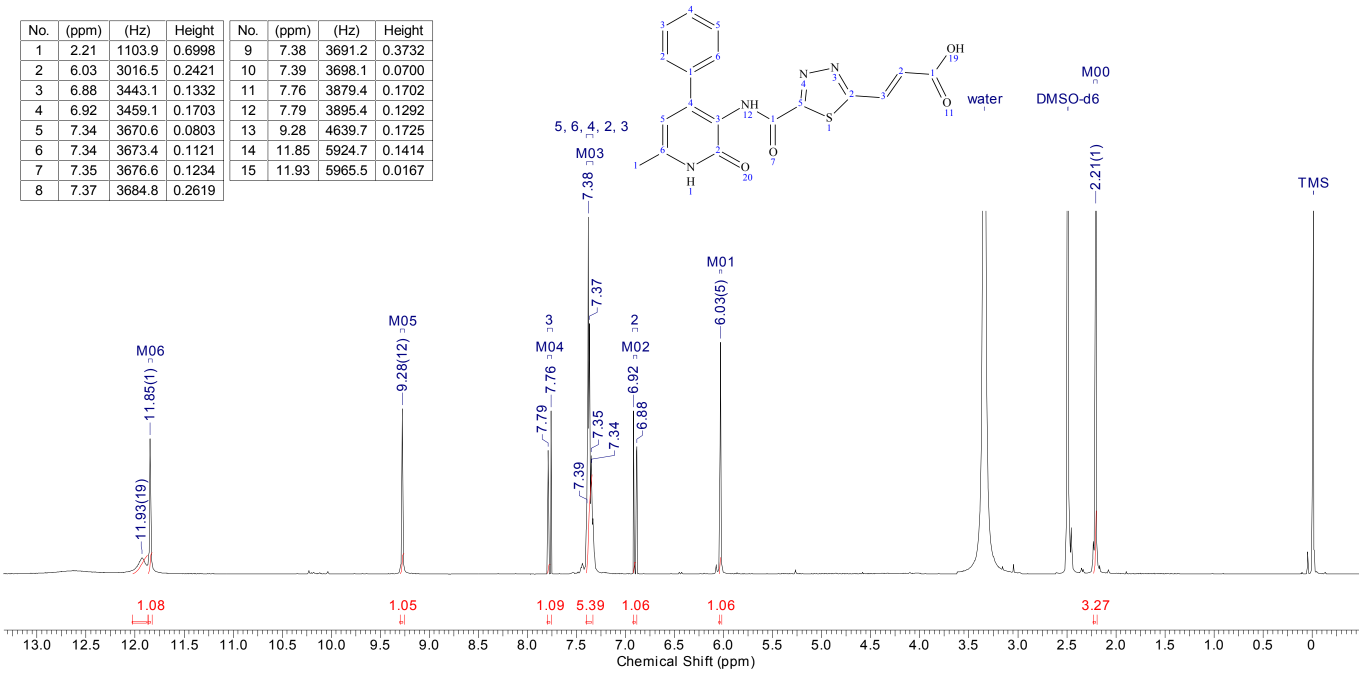

No.	(ppm)	(Hz)	Height	No.	(ppm)	(Hz)	Height
1	18.37	2310.7	0.0327	9	137.44	17284.7	0.0223
2	105.64	13284.6	0.0178	10	139.73	17571.8	0.0132
3	120.23	15119.4	0.0092	11	143.48	18043.2	0.0086
4	126.15	15864.2	0.0287	12	148.99	18736.2	0.0096
5	127.59	16045.8	0.0524	13	159.99	20119.5	0.0112
6	128.17	16118.5	0.0749	14	160.50	20184.0	0.0235
7	128.32	16136.7	0.0299	15	160.83	20225.8	0.0175
8	135.58	17050.4	0.0191	16	170.68	21463.8	0.0278

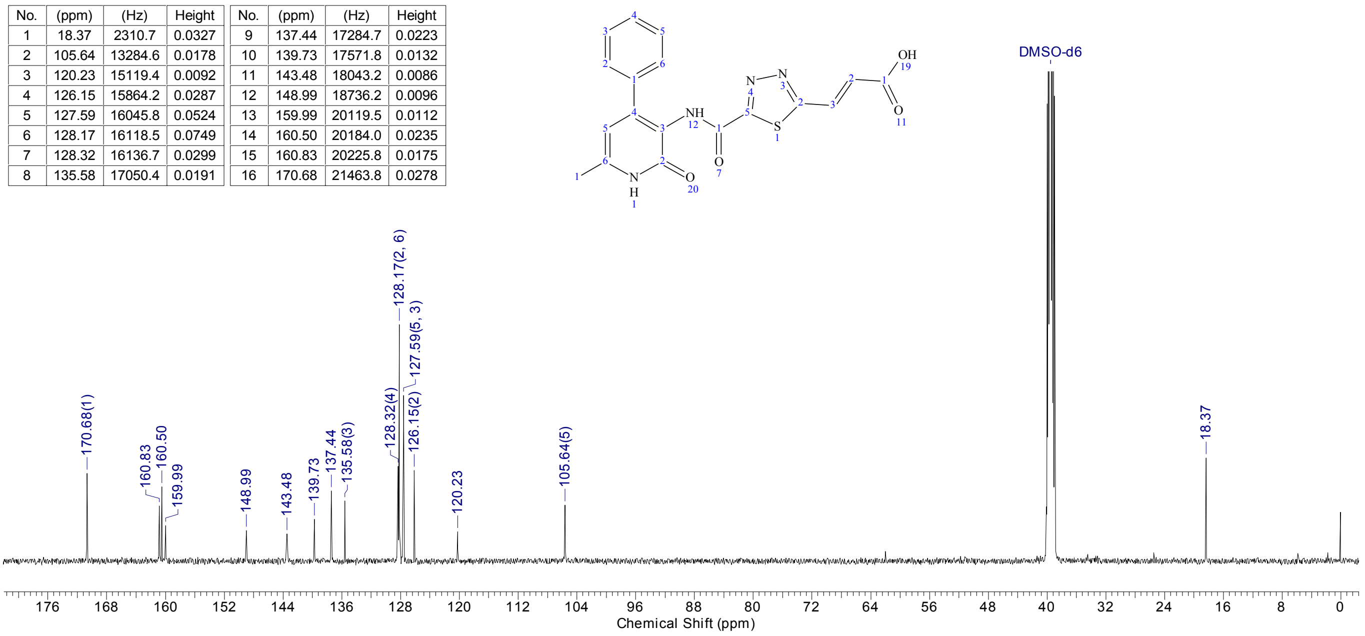

^{1}H (400 MHz, DMSO-d_6) and ^{13}C (100 MHz, DMSO-d_6) NMR Spectra of **8b**

No.	(ppm)	(Hz)	Height	No.	(ppm)	(Hz)	Height
1	2.21	1102.9	0.1491	9	7.66	3829.9	0.0288
2	6.45	3227.9	0.0451	10	7.68	3841.4	0.0275
3	6.93	3464.2	0.0231	11	7.69	3846.4	0.0279
4	6.96	3480.2	0.0277	12	7.77	3885.3	0.0279
5	7.13	3565.3	0.0209	13	7.80	3901.3	0.0235
6	7.14	3570.4	0.0285	14	9.50	4751.0	0.0327
7	7.15	3574.5	0.0219	15	11.75	5877.6	0.0100
8	7.65	3826.7	0.0290	16	11.97	5986.5	0.0327

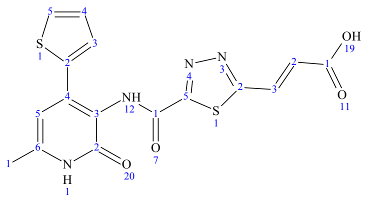

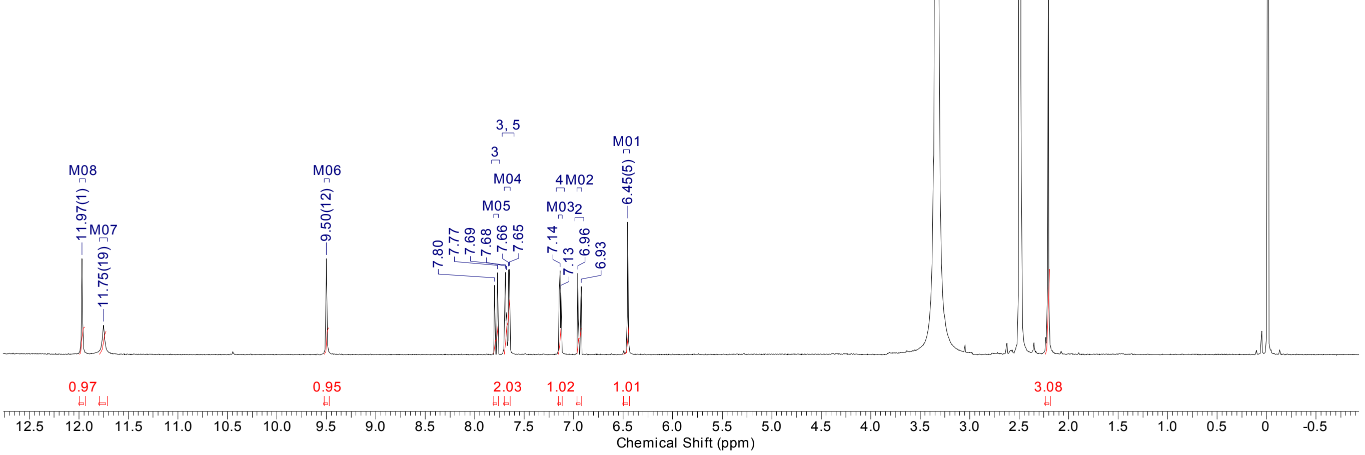

No.	(ppm)	(Hz)	Height	No.	(ppm)	(Hz)	Height
1	18.45	2319.8	0.0078	9	137.19	17253.0	0.0046
2	102.67	12911.3	0.0037	10	139.89	17592.7	0.0046
3	118.59	14914.1	0.0033	11	141.19	17755.2	0.0036
4	126.55	15915.0	0.0079	12	143.27	18016.8	0.0021
5	127.14	15988.6	0.0095	13	160.55	20190.4	0.0062
6	128.93	16213.9	0.0092	14	160.83	20225.8	0.0037
7	129.97	16344.7	0.0091	15	160.93	20238.5	0.0049
8	135.44	17033.1	0.0084	16	170.70	21467.4	0.0054

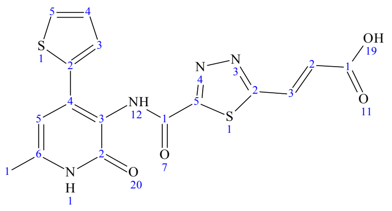

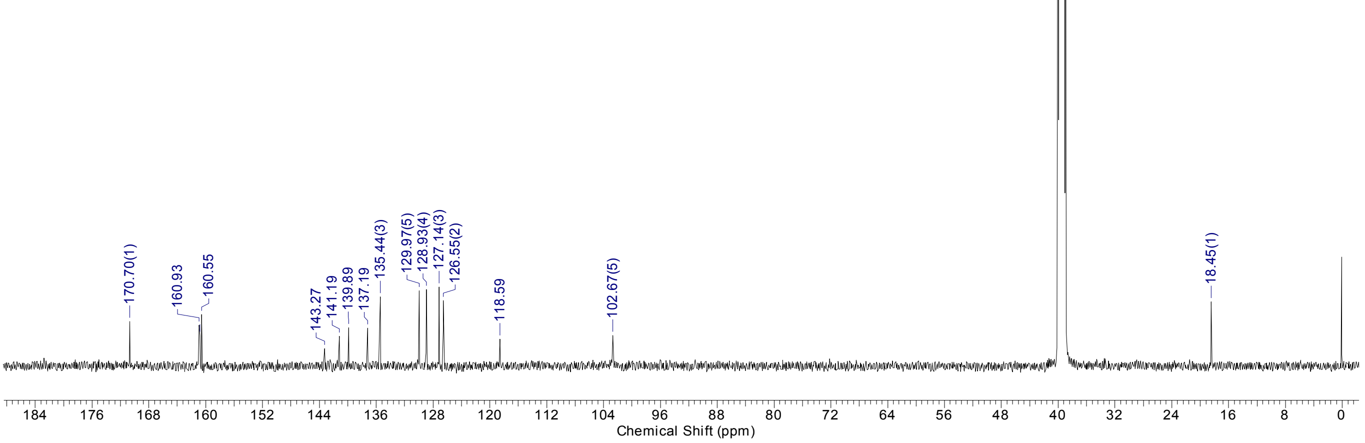

^{1}H (400 MHz, DMSO-d_6) and ^{13}C (100 MHz, DMSO-d_6) NMR Spectra of **8c**

No.	(ppm)	(Hz)	Height	No.	(ppm)	(Hz)	Height	No.	(ppm)	(Hz)	Height
1	2.04	815.0	0.5203	8	7.74	3096.7	0.2903	15	8.01	3203.2	0.1599
2	2.06	824.4	0.8983	9	7.93	3172.1	0.1127	16	8.01	3206.2	0.1273
3	2.16	862.4	1.0000	10	7.93	3175.0	0.1215	17	8.02	3208.4	0.1190
4	5.95	2380.9	0.4521	11	7.95	3181.0	0.1725	18	8.03	3211.7	0.0897
5	7.71	3086.7	0.1555	12	7.96	3184.4	0.1348	19	9.64	3857.3	0.1295
6	7.72	3089.0	0.1541	13	7.96	3186.8	0.1361	20	10.08	4035.0	0.1976
7	7.73	3094.7	0.3319	14	7.97	3189.7	0.1682	21	11.74	4698.6	0.0197

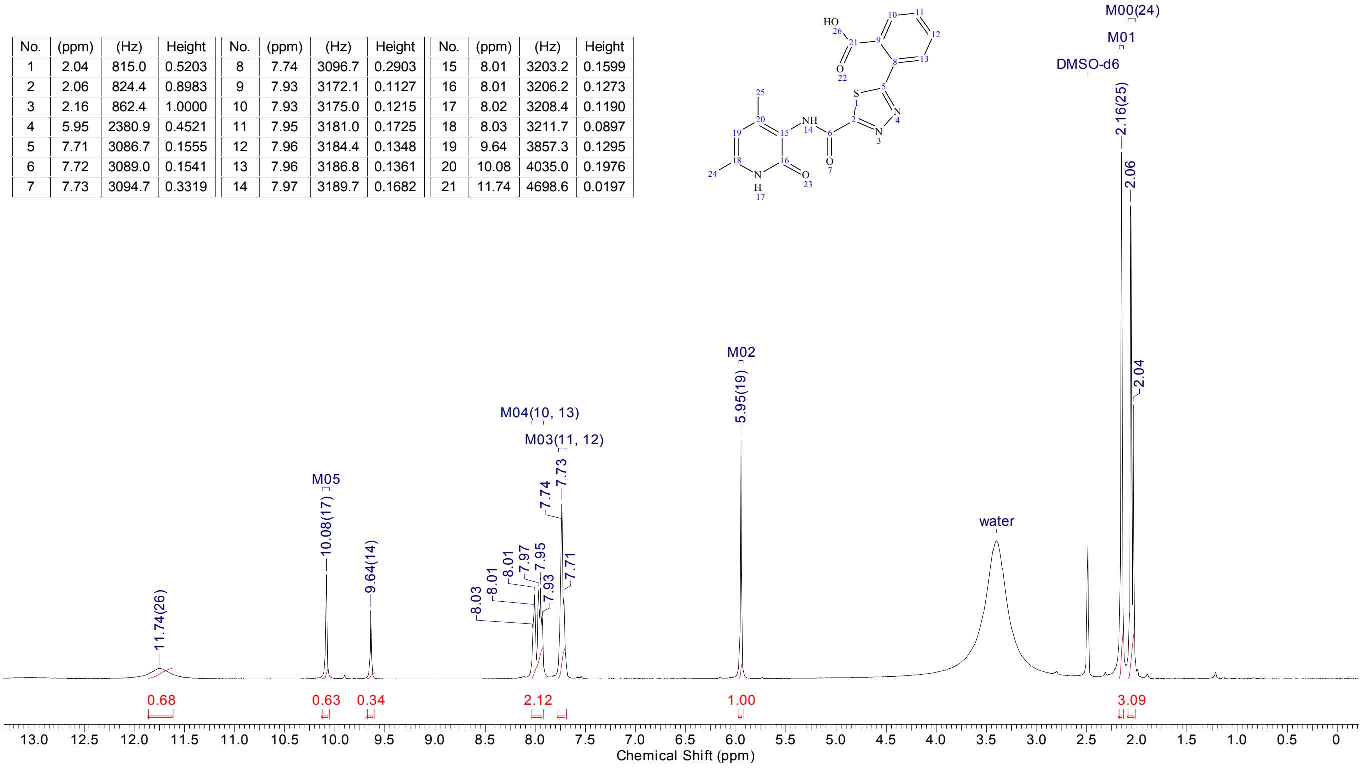

No.	(ppm)	(Hz)	Height	No.	(ppm)	(Hz)	Height
1	18.06	1817.3	-0.2181	10	135.65	13647.8	-0.1590
2	18.23	1834.1	-0.2140	11	142.93	14380.3	0.1068
3	106.55	10720.3	-0.1990	12	147.39	14828.9	0.0867
4	121.24	12198.2	0.0825	13	156.28	15723.8	0.0760
5	124.11	12486.6	-0.1903	14	159.75	16073.3	0.1215
6	129.25	13004.6	0.1070	15	166.09	16710.3	0.1225
7	129.97	13077.1	-0.1723	16	167.71	16873.6	0.0969
8	131.73	13254.1	-0.1881	17	170.35	17139.1	0.0602
9	132.52	13333.5	0.0985				

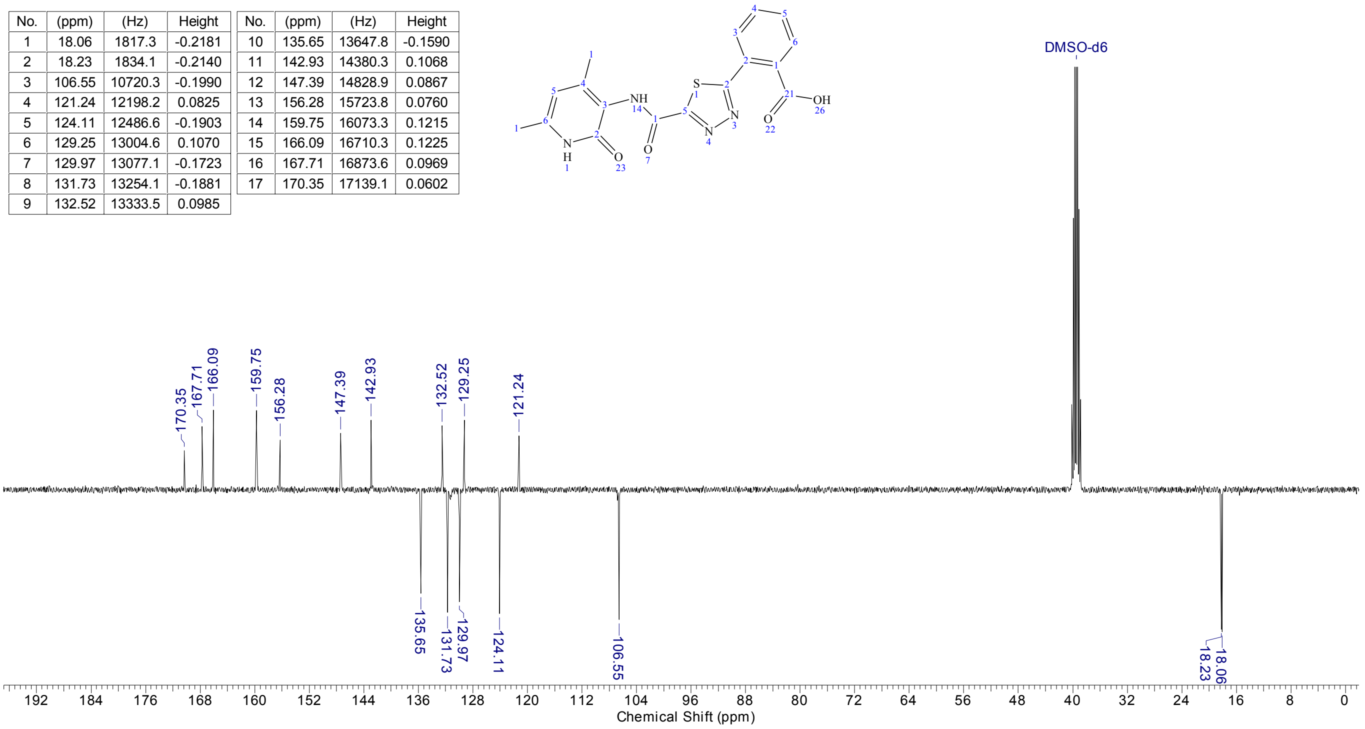

^{1}H (400 MHz, DMSO-d_6) and ^{13}C (100 MHz, DMSO-d_6) NMR Spectra of **9a**

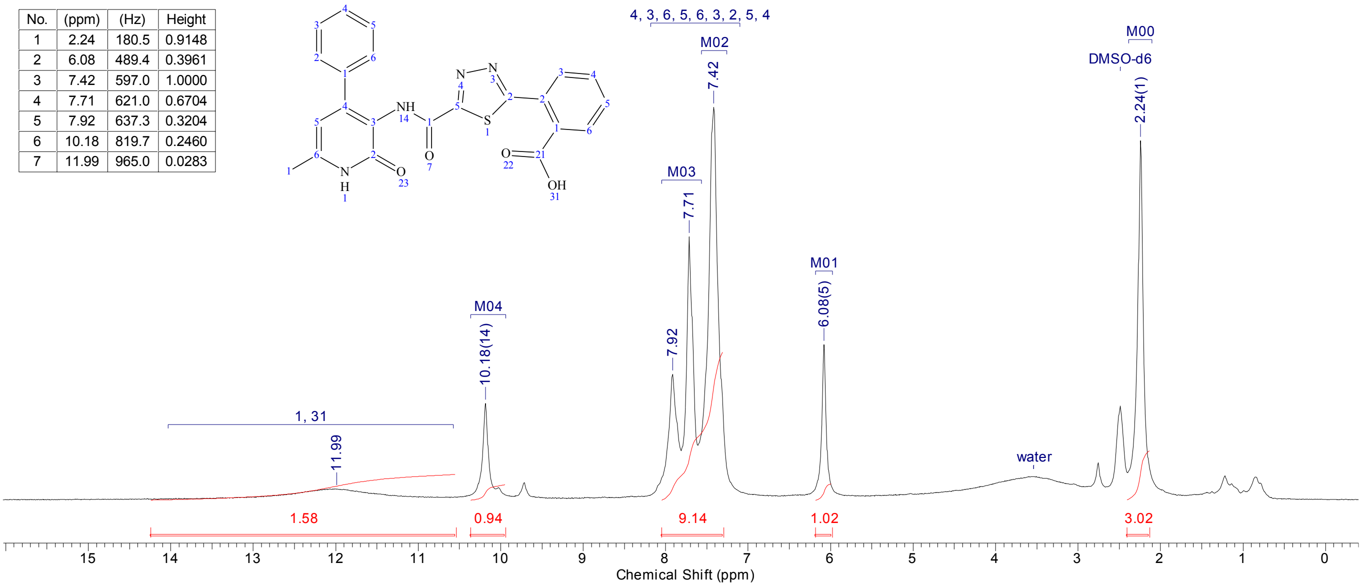

No.	(ppm)	(Hz)	Height
1	2.24	180.5	0.9148
2	6.08	489.4	0.3961
3	7.42	597.0	1.0000
4	7.71	621.0	0.6704
5	7.92	637.3	0.3204
6	10.18	819.7	0.2460
7	11.99	965.0	0.0283

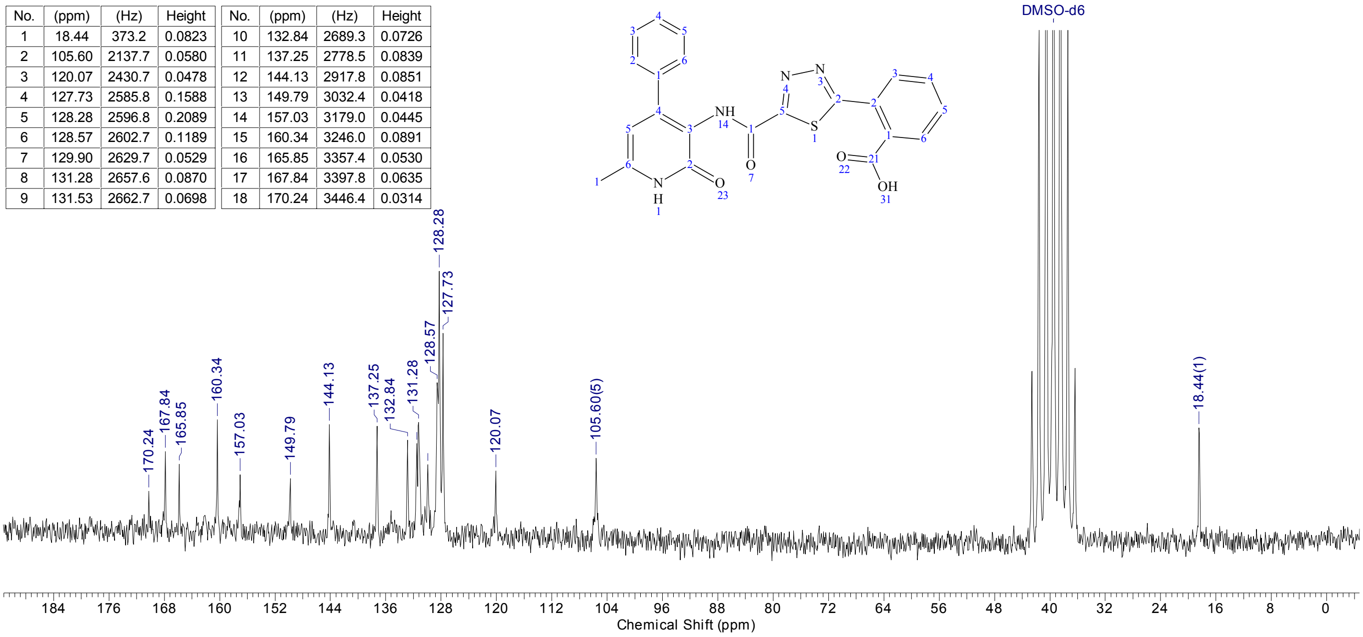

No.	(ppm)	(Hz)	Height	No.	(ppm)	(Hz)	Height
1	18.44	373.2	0.0823	10	132.84	2689.3	0.0726
2	105.60	2137.7	0.0580	11	137.25	2778.5	0.0839
3	120.07	2430.7	0.0478	12	144.13	2917.8	0.0851
4	127.73	2585.8	0.1588	13	149.79	3032.4	0.0418
5	128.28	2596.8	0.2089	14	157.03	3179.0	0.0445
6	128.57	2602.7	0.1189	15	160.34	3246.0	0.0891
7	129.90	2629.7	0.0529	16	165.85	3357.4	0.0530
8	131.28	2657.6	0.0870	17	167.84	3397.8	0.0635
9	131.53	2662.7	0.0698	18	170.24	3446.4	0.0314

^{1}H (80 MHz, DMSO-d_6) and ^{13}C (21 MHz, DMSO-d_6) NMR Spectra of **9b**

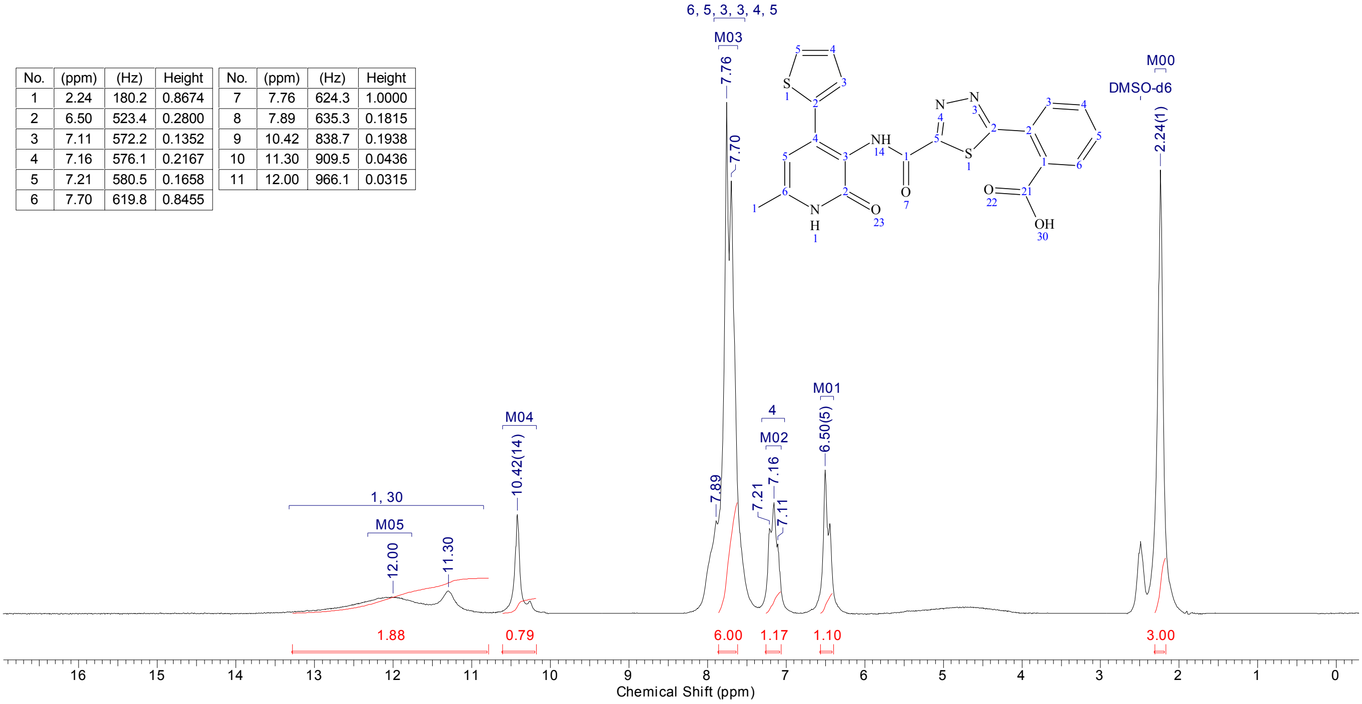

No.	(ppm)	(Hz)	Height
1	2.24	180.2	0.8674
2	6.50	523.4	0.2800
3	7.11	572.2	0.1352
4	7.16	576.1	0.2167
5	7.21	580.5	0.1658
6	7.70	619.8	0.8455

No.	(ppm)	(Hz)	Height
7	7.76	624.3	1.0000
8	7.89	635.3	0.1815
9	10.42	838.7	0.1938
10	11.30	909.5	0.0436
11	12.00	966.1	0.0315

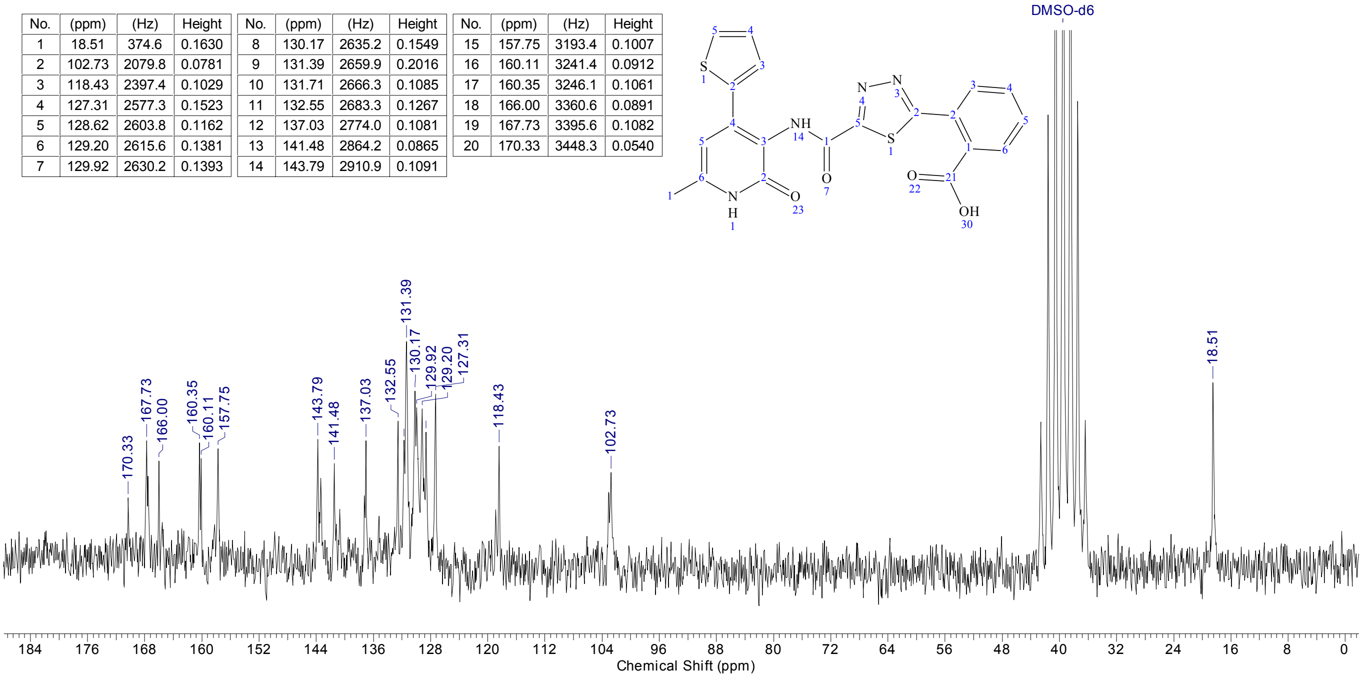

No.	(ppm)	(Hz)	Height
1	18.51	374.6	0.1630
2	102.73	2079.8	0.0781
3	118.43	2397.4	0.1029
4	127.31	2577.3	0.1523
5	128.62	2603.8	0.1162
6	129.20	2615.6	0.1381
7	129.92	2630.2	0.1393

No.	(ppm)	(Hz)	Height
8	130.17	2635.2	0.1549
9	131.39	2659.9	0.2016
10	131.71	2666.3	0.1085
11	132.55	2683.3	0.1267
12	137.03	2774.0	0.1081
13	141.48	2864.2	0.0865
14	143.79	2910.9	0.1091

No.	(ppm)	(Hz)	Height
15	157.75	3193.4	0.1007
16	160.11	3241.4	0.0912
17	160.35	3246.1	0.1061
18	166.00	3360.6	0.0891
19	167.73	3395.6	0.1082
20	170.33	3448.3	0.0540

^{1}H (80 MHz, DMSO-d_6) and ^{13}C (21 MHz, DMSO-d_6) NMR Spectra of **9c**

Copies of MS Spectra of Products

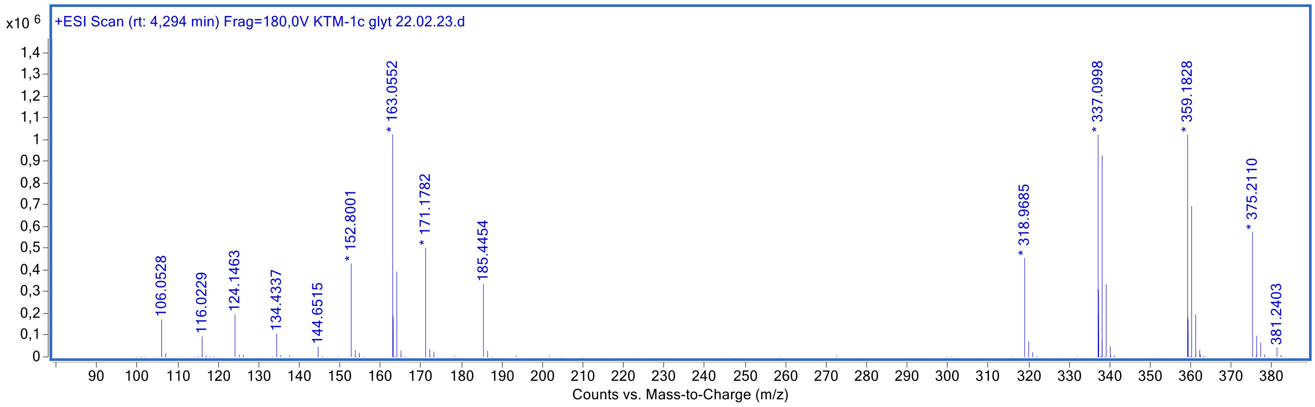

Mass spectrum (LC/Q-TOF) of (**7a**)

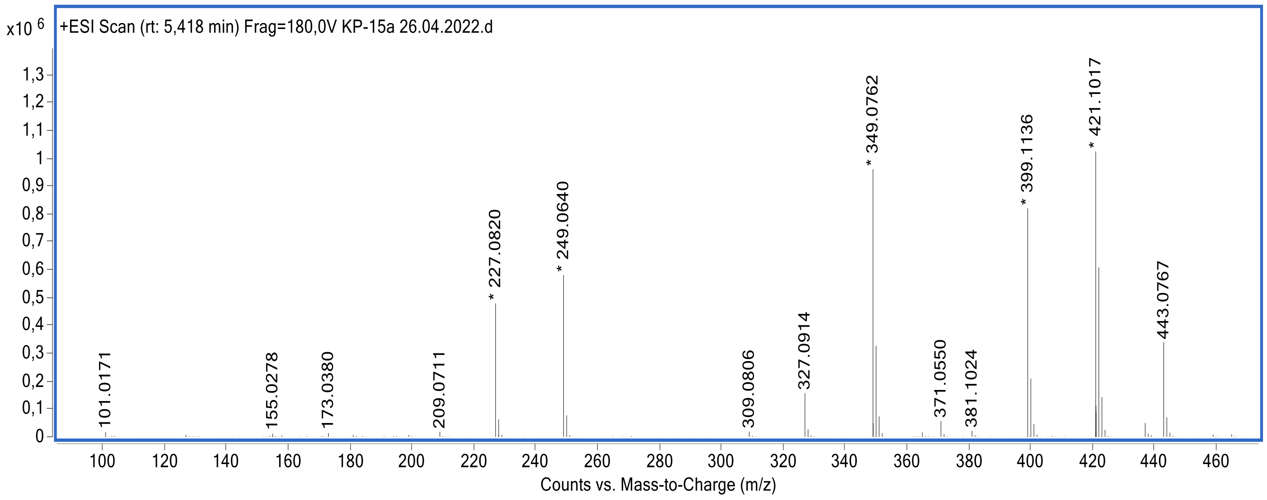

Mass spectrum (LC/Q-TOF) of (**7b**)

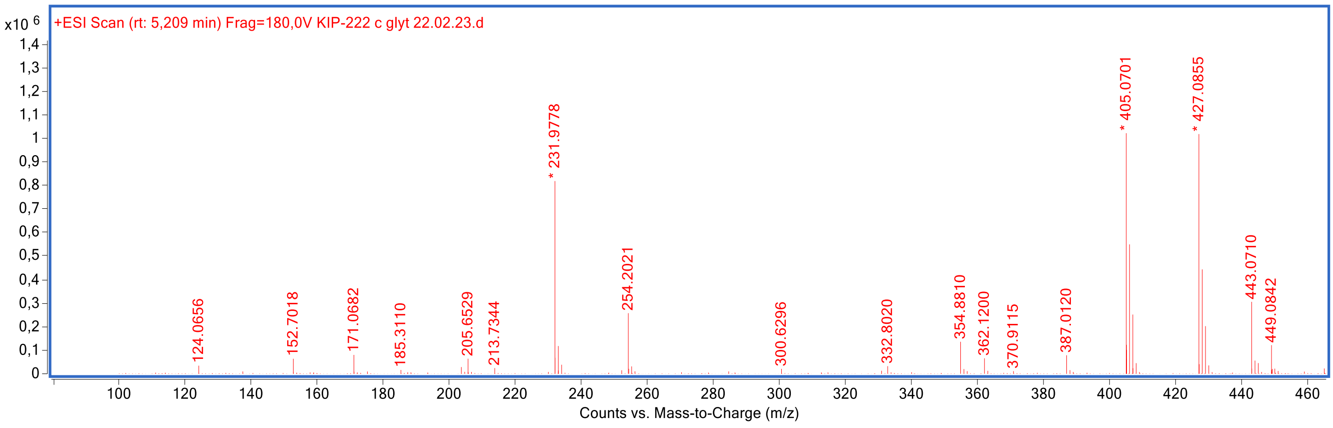

Mass spectrum (LC/Q-TOF) of (**7c**)

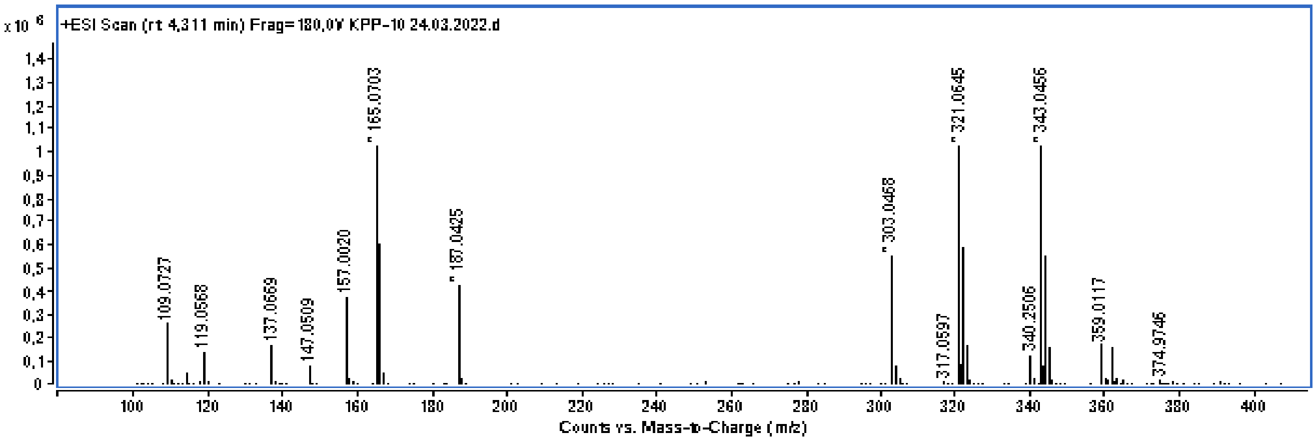

Mass spectrum (LC/Q-TOF) of (**8a**)

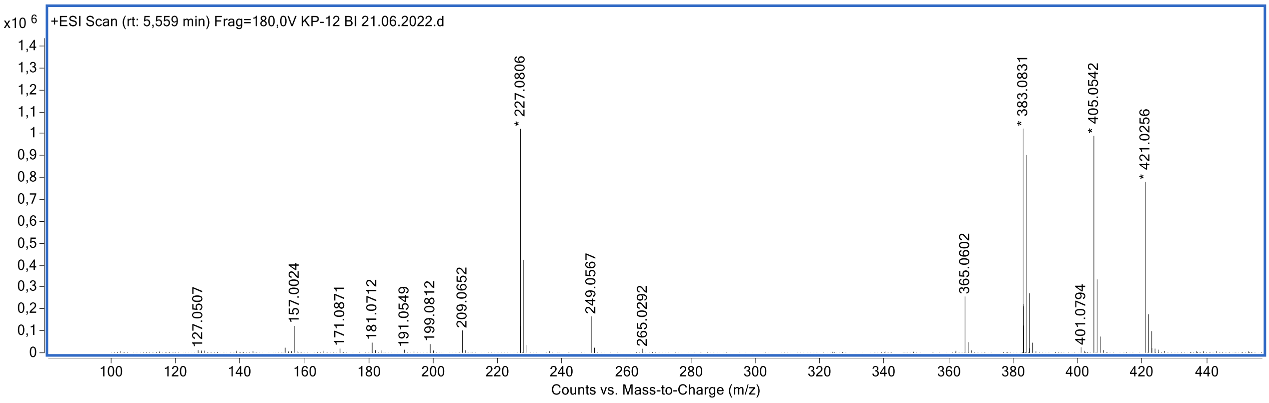

Mass spectrum (LC/Q-TOF) of (**8b**)

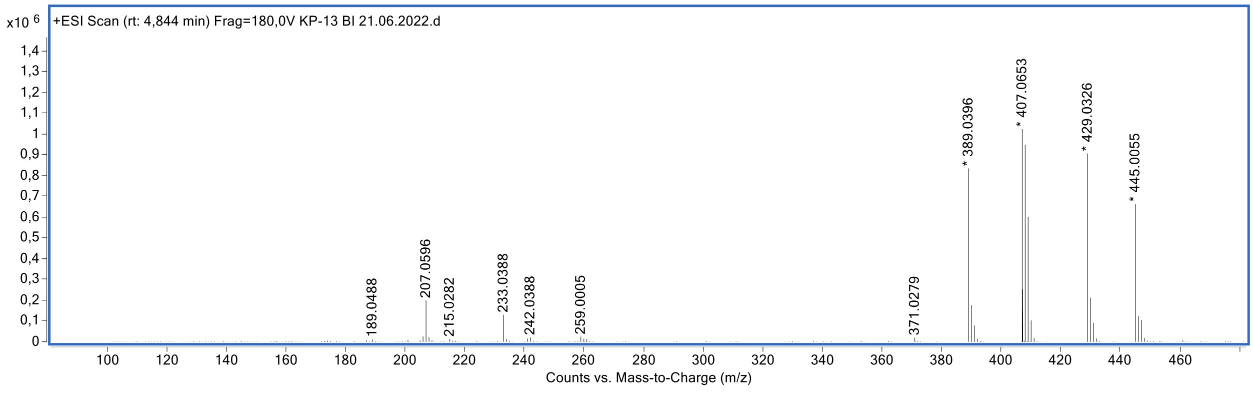

Mass spectrum (LC/Q-TOF) of (**8c**)

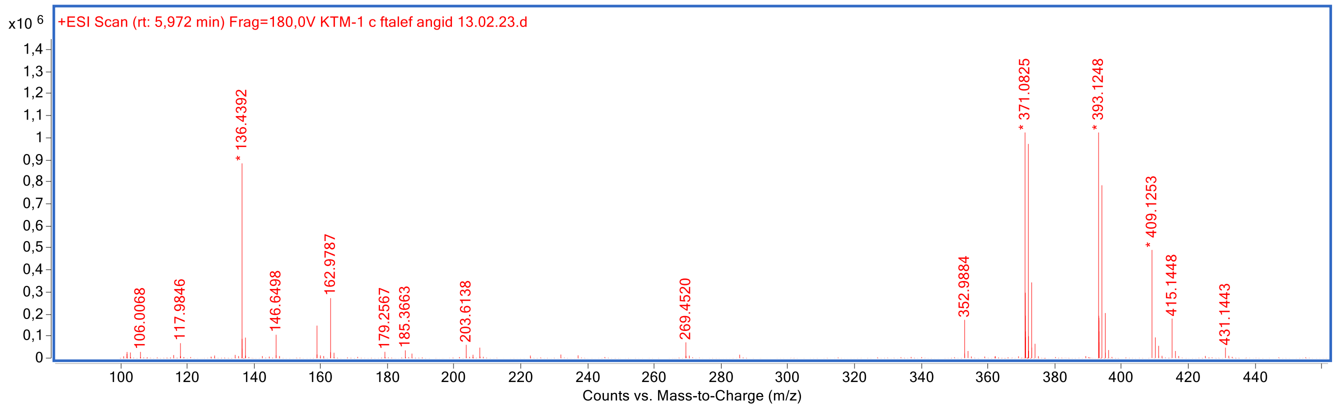

Mass spectrum (LC/Q-TOF) of (**9a**)

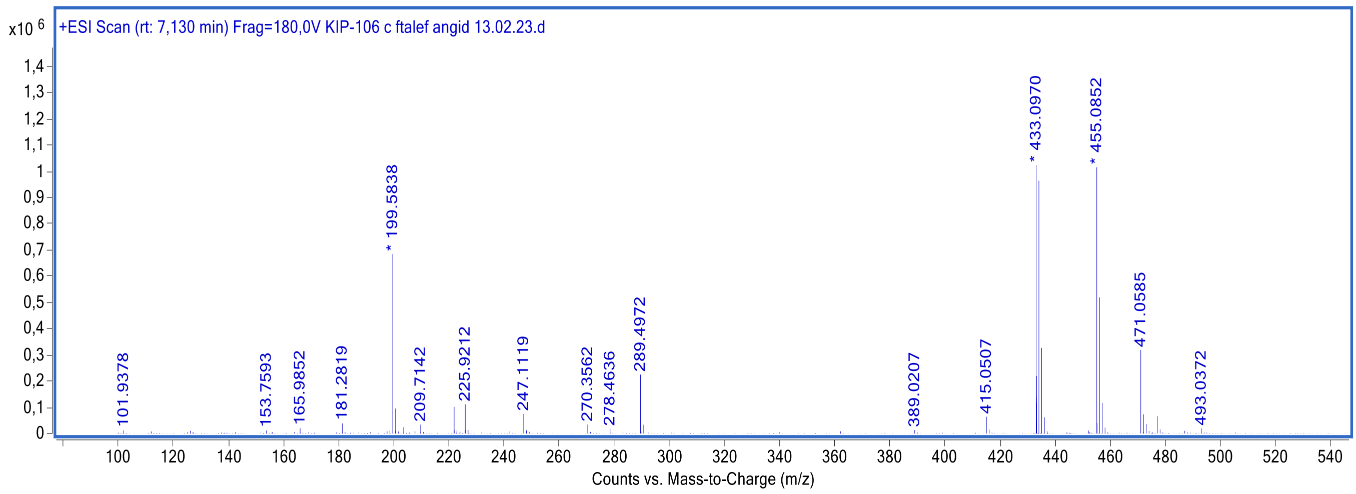

Mass spectrum (LC/Q-TOF) of (**9b**)

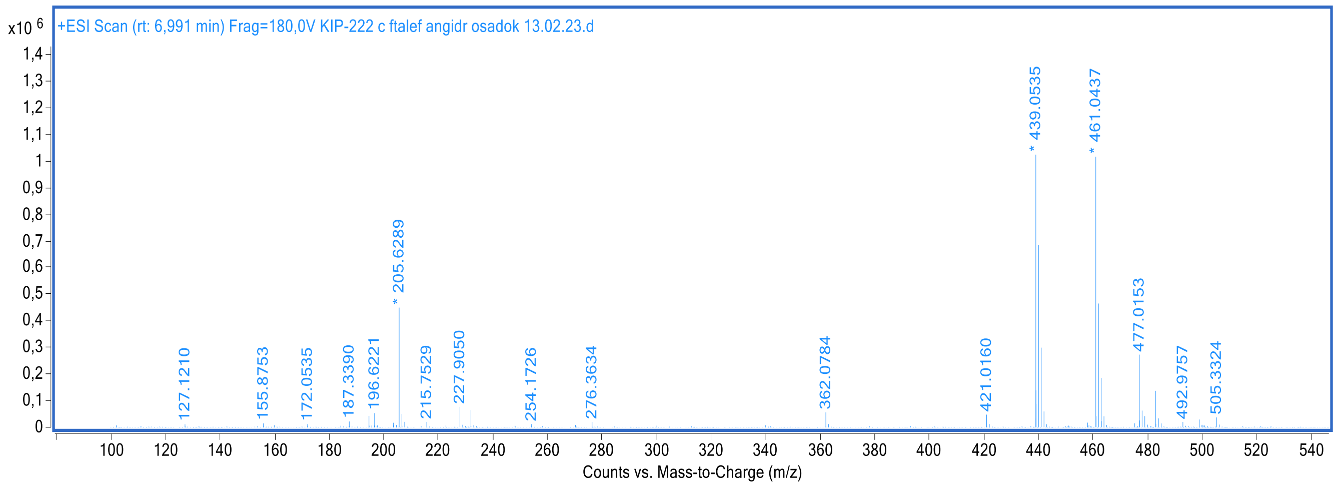

Mass spectrum (LC/Q-TOF) of (**9c**)

Table S1. Complexes between synthesized derivatives **5(a-c), 7-9(a-c)** and active sites of proteins (PDB: 5NN8, 3W37, 2QV4)

<table>
<tr><th>5NN8</th><th>3W37</th><th>2QV4</th></tr>
<tr>
<td>
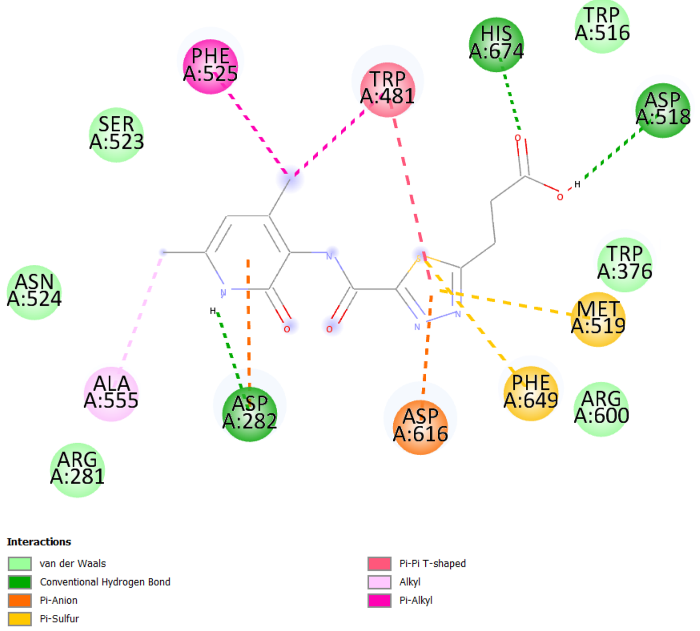

5a
</td>
<td>
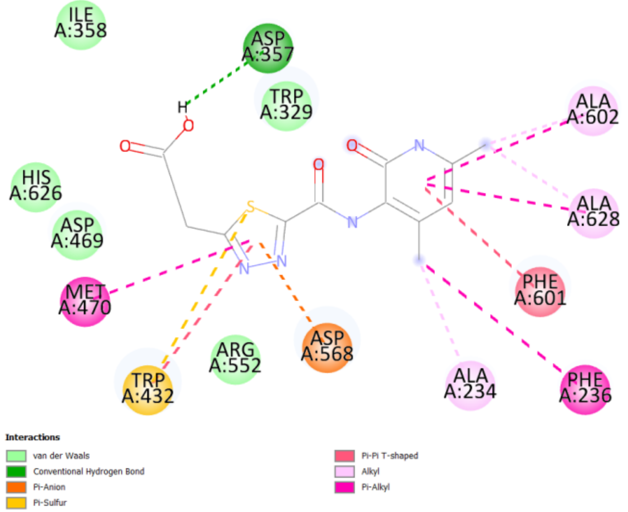

5a
</td>
<td>
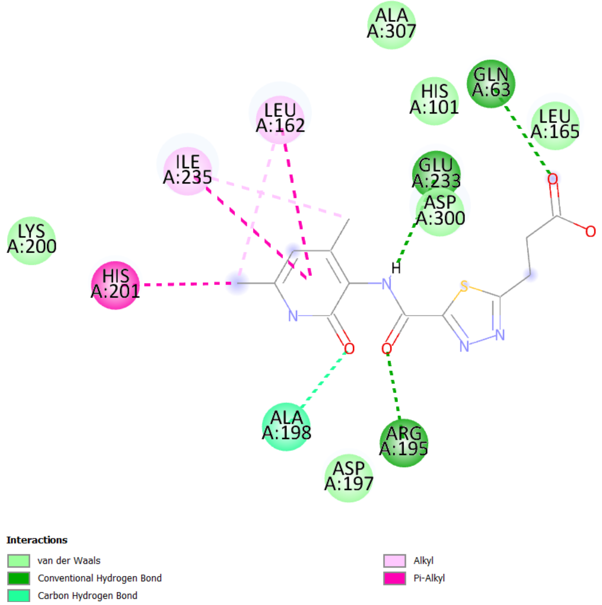

5a
</td>
</tr>
</table>

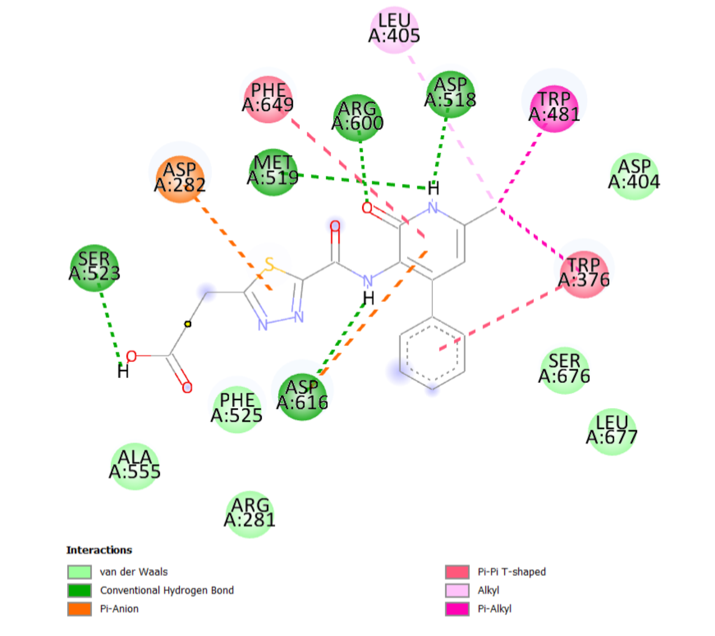

5b

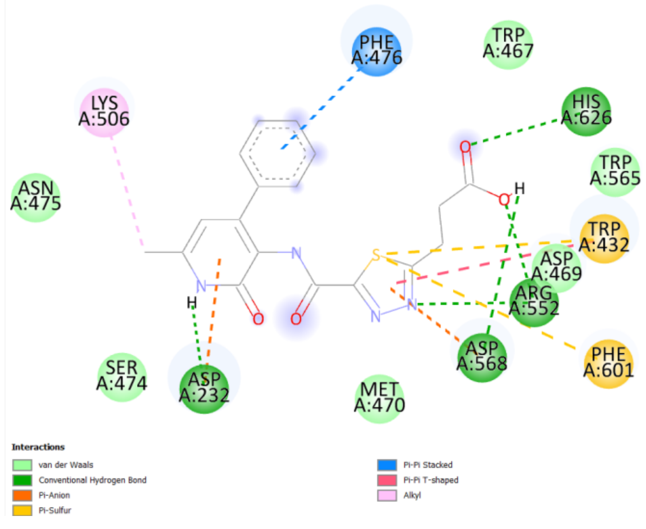

5b

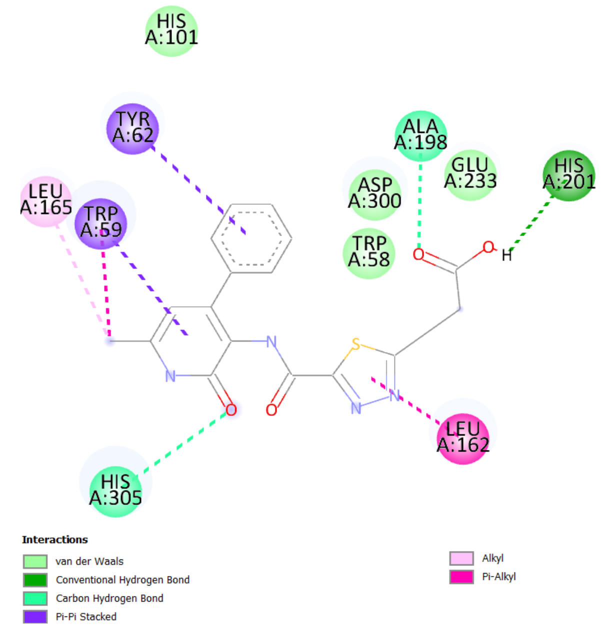

5b

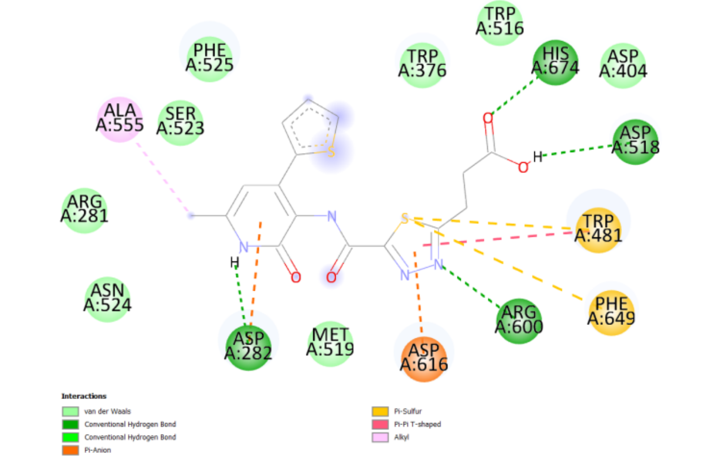

5c

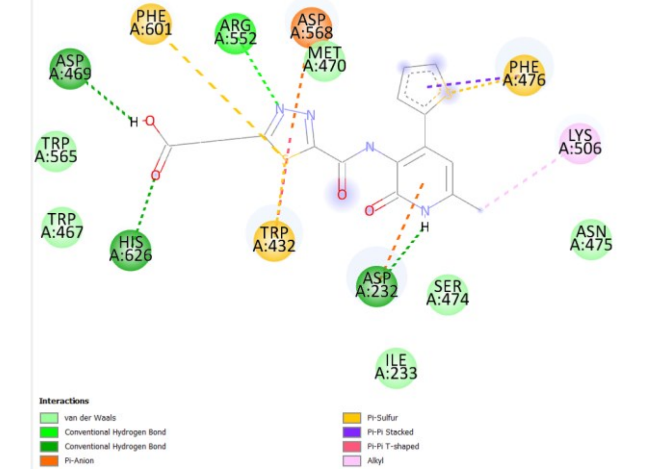

5c

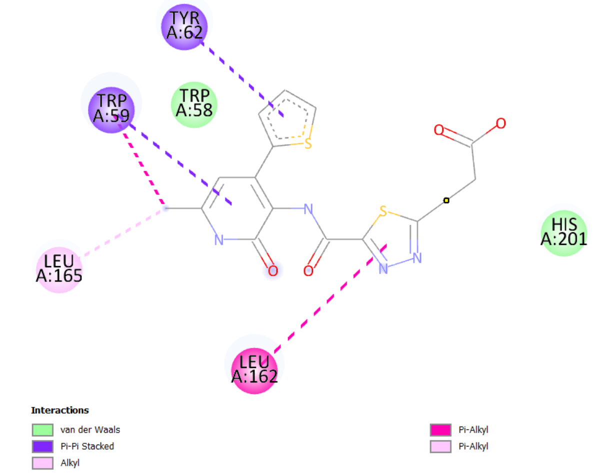

5c

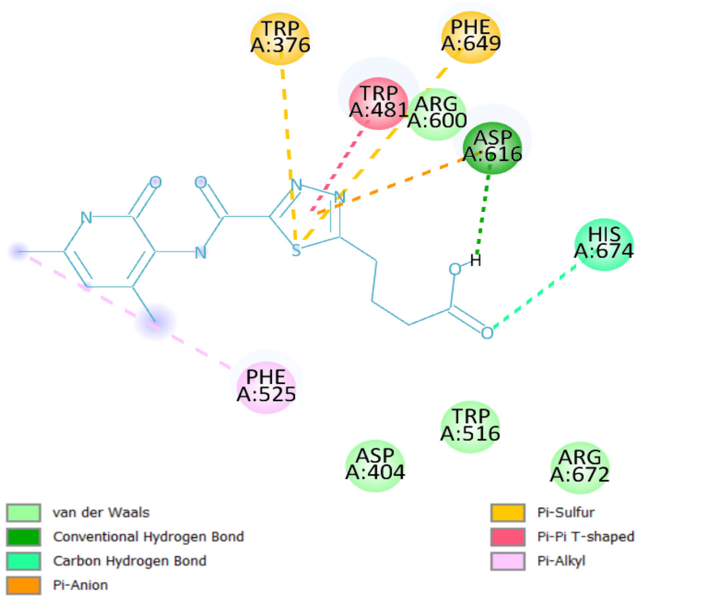
TRP A:376
PHE A:649
TRP A:481
ARG A:600
ASP A:616
HIS A:674
PHE A:525
TRP A:516
ASP A:404
ARG A:672
van der Waals
Conventional Hydrogen Bond
Carbon Hydrogen Bond
Pi-Anion
Pi-Sulfur
Pi-Pi T-shaped
Pi-Alkyl

7a

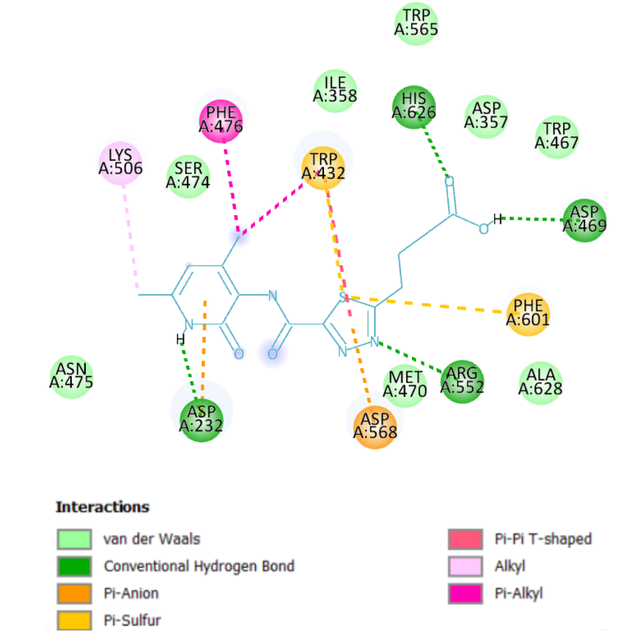
TRP A:565
ILE A:358
HIS A:626
ASP A:357
TRP A:467
PHE A:476
LYS A:506
SER A:474
TRP A:432
ASP A:469
PHE A:601
ASN A:475
ASP A:232
MET A:470
ARG A:552
ALA A:628
ASP A:568
Interactions
van der Waals
Conventional Hydrogen Bond
Pi-Anion
Pi-Sulfur
Pi-Pi T-shaped
Alkyl
Pi-Alkyl

7a

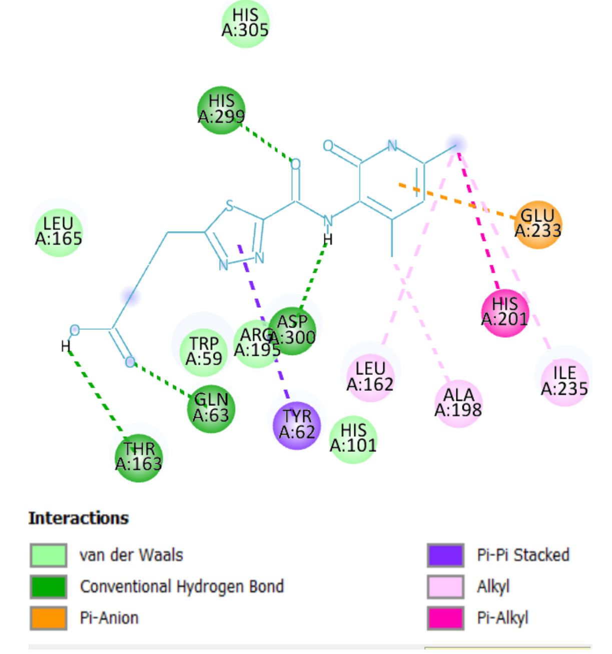
HIS A:305
HIS A:299
LEU A:165
GLU A:233
HIS A:201
TRP A:59
ARG A:195
ASP A:300
LEU A:162
ILE A:235
ALA A:198
GLN A:63
TYR A:62
HIS A:101
THR A:163
Interactions
van der Waals
Conventional Hydrogen Bond
Pi-Anion
Pi-Pi Stacked
Alkyl
Pi-Alkyl

7a

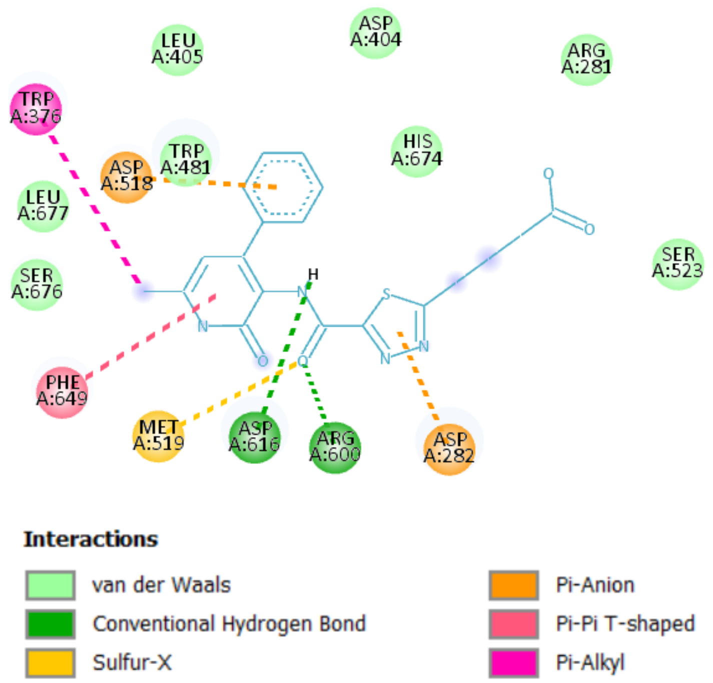
LEU A:405
ASP A:404
ARG A:281
TRP A:376
ASP A:518
TRP A:481
HIS A:674
LEU A:677
SER A:523
SER A:676
PHE A:649
MET A:519
ASP A:616
ARG A:600
ASP A:282
Interactions
van der Waals
Conventional Hydrogen Bond
Sulfur-X
Pi-Anion
Pi-Pi T-shaped
Pi-Alkyl

7b

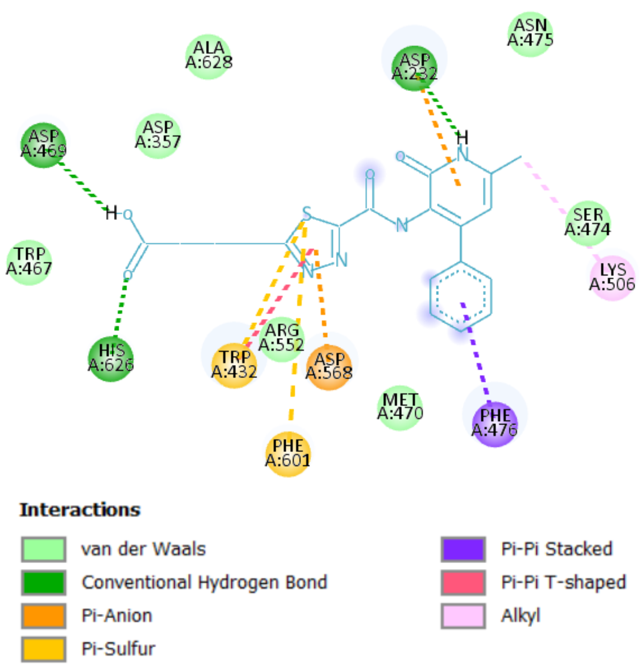
ALA A:628
ASP A:232
ASN A:475
ASP A:469
ASP A:357
SER A:474
TRP A:467
LYS A:506
HIS A:626
ARG A:552
TRP A:432
ASP A:568
MET A:470
PHE A:476
PHE A:601
Interactions
van der Waals
Conventional Hydrogen Bond
Pi-Anion
Pi-Sulfur
Pi-Pi Stacked
Pi-Pi T-shaped
Alkyl

7b

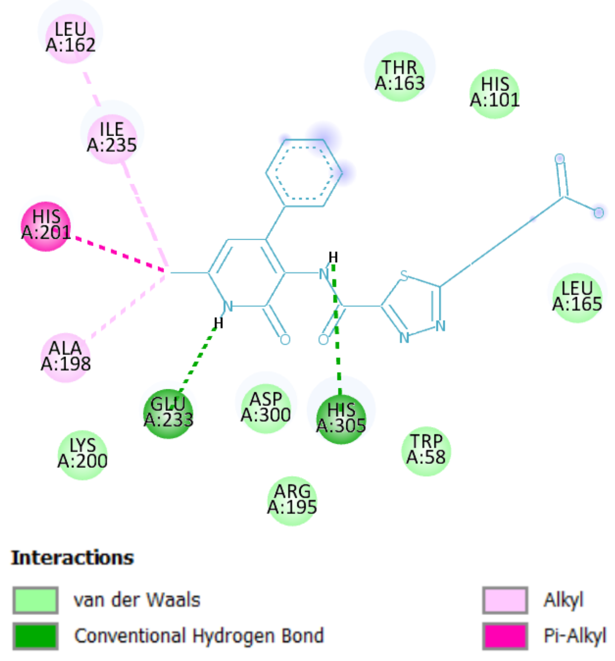
LEU A:162
THR A:163
HIS A:101
ILE A:235
HIS A:201
LEU A:165
ALA A:198
GLU A:233
ASP A:300
HIS A:305
LYS A:200
TRP A:58
ARG A:195
Interactions
van der Waals
Conventional Hydrogen Bond
Alkyl
Pi-Alkyl

7b

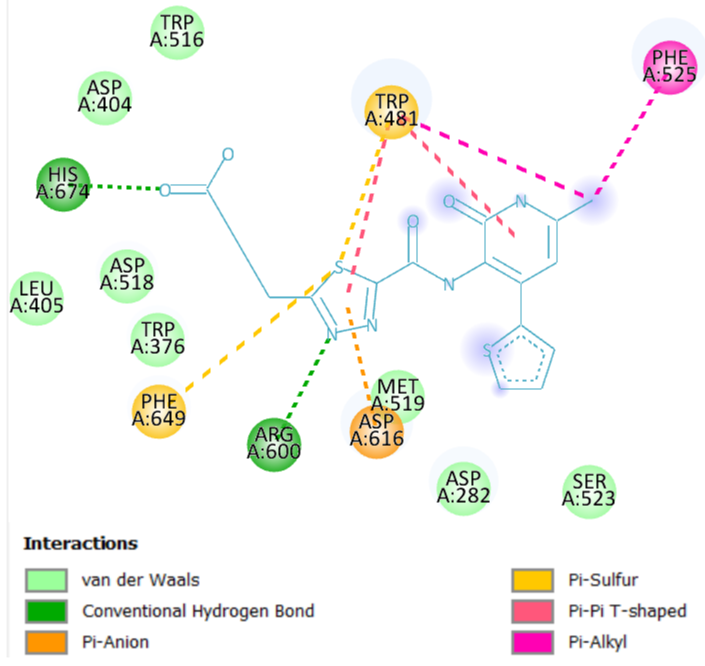
TRP A:516
ASP A:404
TRP A:481
PHE A:525
HIS A:674
LEU A:405
ASP A:518
TRP A:376
PHE A:649
ARG A:600
MET A:519
ASP A:616
ASP A:282
SER A:523
Interactions
van der Waals
Conventional Hydrogen Bond
Pi-Anion
Pi-Sulfur
Pi-Pi T-shaped
Pi-Alkyl

7c

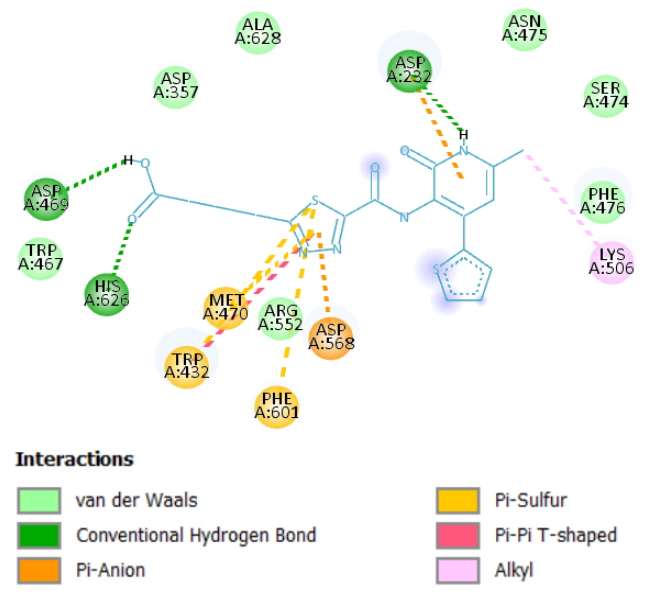
ALA A:628
ASN A:475
ASP A:357
ASP A:232
SER A:474
ASP A:469
PHE A:476
TRP A:467
LYS A:506
HIS A:626
MET A:470
ARG A:552
ASP A:568
TRP A:432
PHE A:601
Interactions
van der Waals
Conventional Hydrogen Bond
Pi-Anion
Pi-Sulfur
Pi-Pi T-shaped
Alkyl

7c

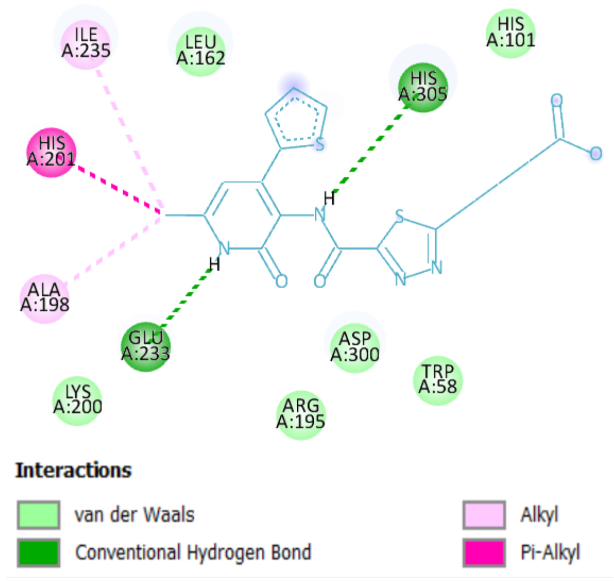
ILE A:235
LEU A:162
HIS A:101
HIS A:305
HIS A:201
ALA A:198
GLU A:233
ASP A:300
TRP A:58
LYS A:200
ARG A:195
Interactions
van der Waals
Conventional Hydrogen Bond
Alkyl
Pi-Alkyl

7c

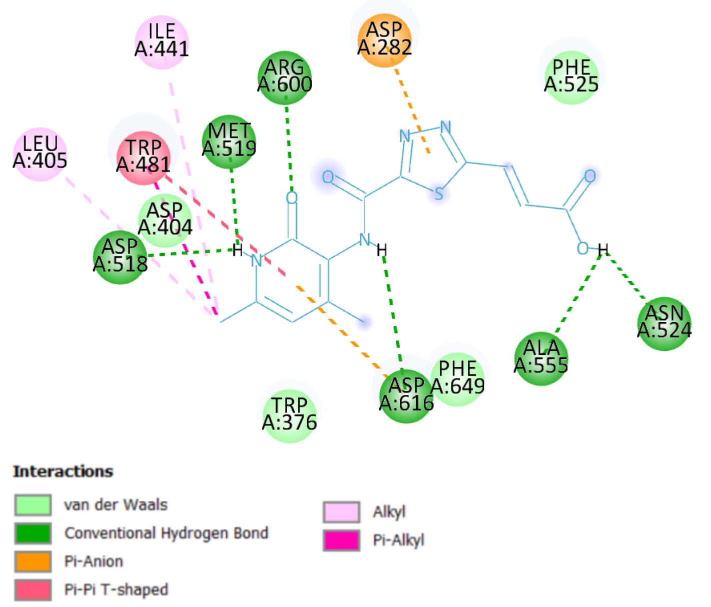
ILE A:441
ASP A:282
ARG A:600
PHE A:525
LEU A:405
TRP A:481
MET A:519
ASP A:404
ASP A:518
ASN A:524
ALA A:555
PHE A:649
ASP A:616
TRP A:376
Interactions
van der Waals
Conventional Hydrogen Bond
Pi-Anion
Pi-Pi T-shaped
Alkyl
Pi-Alkyl

8a

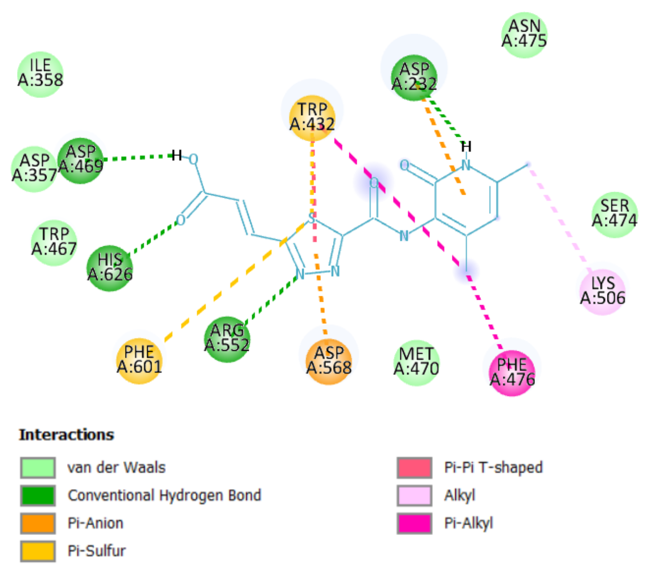
ILE A:358
ASN A:475
ASP A:232
TRP A:432
ASP A:357
ASP A:469
SER A:474
TRP A:467
HIS A:626
LYS A:506
PHE A:601
ARG A:552
ASP A:568
MET A:470
PHE A:476
Interactions
van der Waals
Conventional Hydrogen Bond
Pi-Anion
Pi-Sulfur
Pi-Pi T-shaped
Alkyl
Pi-Alkyl

8a

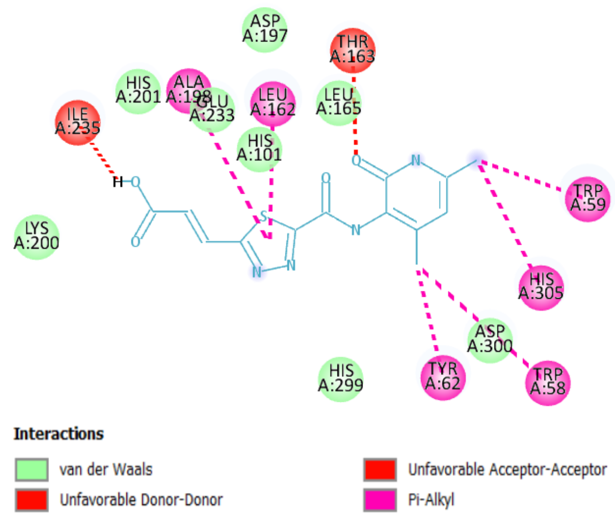
ASP A:197
THR A:163
HIS A:201
ALA A:198
GLU A:233
LEU A:162
LEU A:165
ILE A:235
HIS A:101
TRP A:59
LYS A:200
HIS A:305
ASP A:300
HIS A:299
TYR A:62
TRP A:58
Interactions
van der Waals
Unfavorable Donor-Donor
Unfavorable Acceptor-Acceptor
Pi-Alkyl

8a

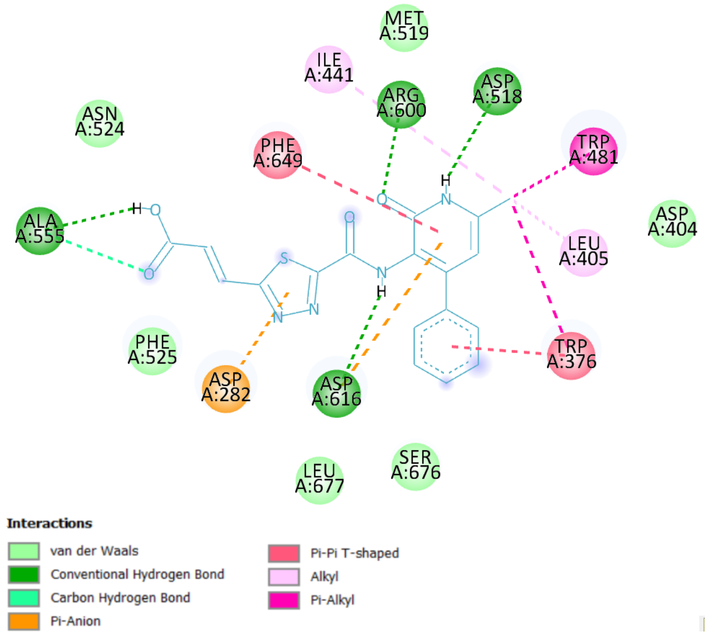
MET A:519
ILE A:441
ARG A:600
ASP A:518
ASN A:524
PHE A:649
TRP A:481
ALA A:555
LEU A:405
ASP A:404
PHE A:525
ASP A:282
ASP A:616
TRP A:376
LEU A:677
SER A:676
Interactions
van der Waals
Conventional Hydrogen Bond
Carbon Hydrogen Bond
Pi-Anion
Pi-Pi T-shaped
Alkyl
Pi-Alkyl

8b

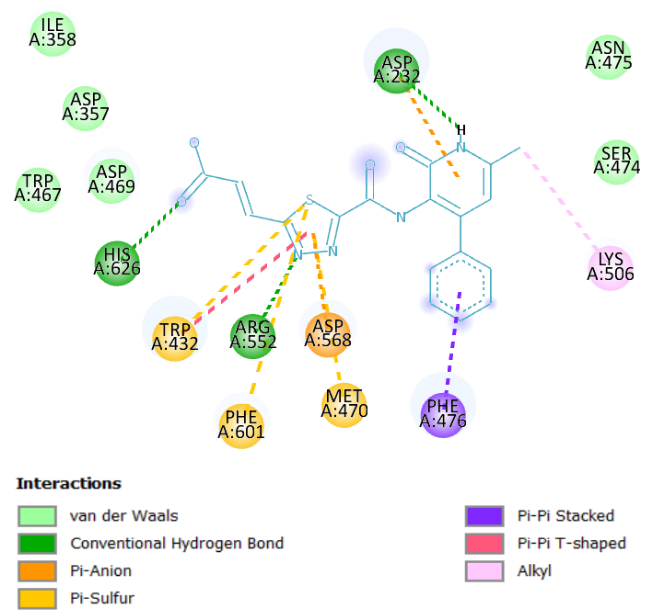
ILE A:358
ASP A:232
ASN A:475
ASP A:357
SER A:474
TRP A:467
ASP A:469
LYS A:506
HIS A:626
TRP A:432
ARG A:552
ASP A:568
PHE A:601
MET A:470
PHE A:476
Interactions
van der Waals
Conventional Hydrogen Bond
Pi-Anion
Pi-Sulfur
Pi-Pi Stacked
Pi-Pi T-shaped
Alkyl

8b

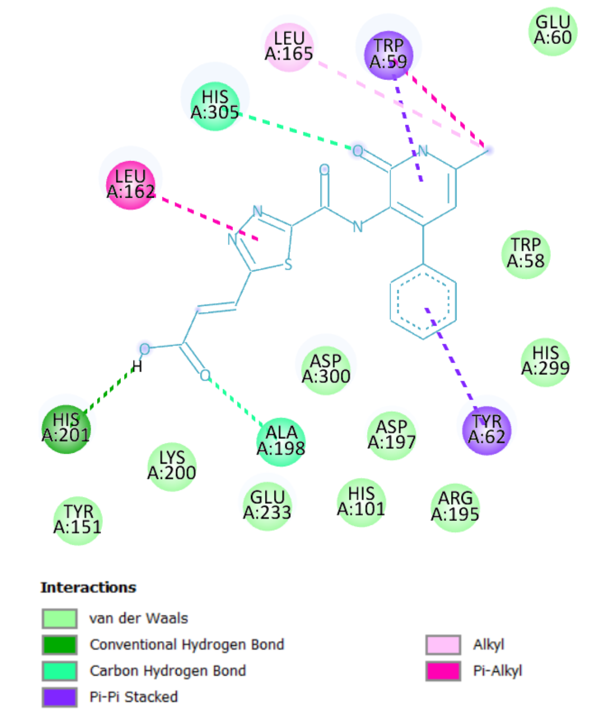
LEU A:165
TRP A:59
GLU A:60
HIS A:305
LEU A:162
TRP A:58
ASP A:300
HIS A:299
HIS A:201
ALA A:198
ASP A:197
TYR A:62
LYS A:200
TYR A:151
GLU A:233
HIS A:101
ARG A:195
Interactions
van der Waals
Conventional Hydrogen Bond
Carbon Hydrogen Bond
Pi-Pi Stacked
Alkyl
Pi-Alkyl

8b

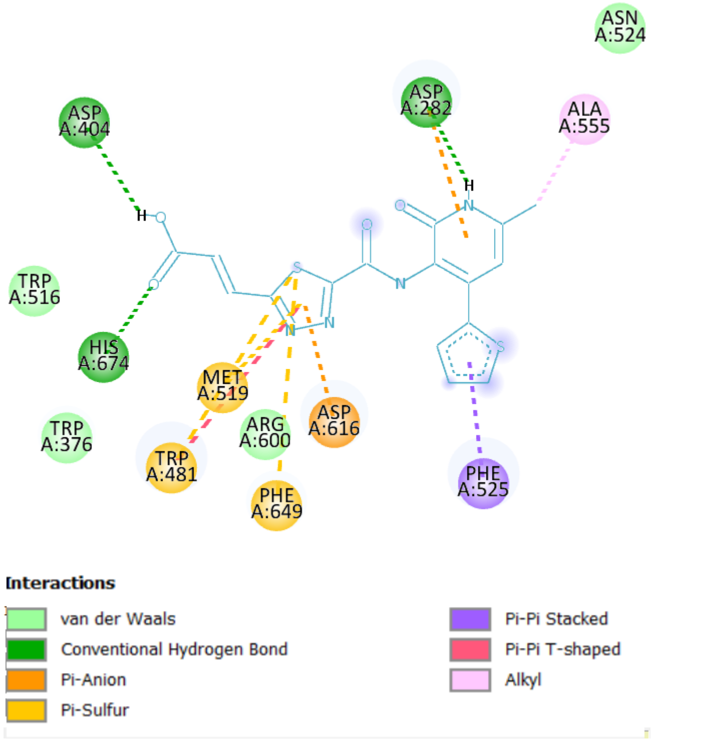
ASN A:524
ASP A:282
ALA A:555
ASP A:404
TRP A:516
HIS A:674
MET A:519
TRP A:376
TRP A:481
ARG A:600
ASP A:616
PHE A:649
PHE A:525
Interactions
van der Waals
Conventional Hydrogen Bond
Pi-Anion
Pi-Sulfur
Pi-Pi Stacked
Pi-Pi T-shaped
Alkyl

8c

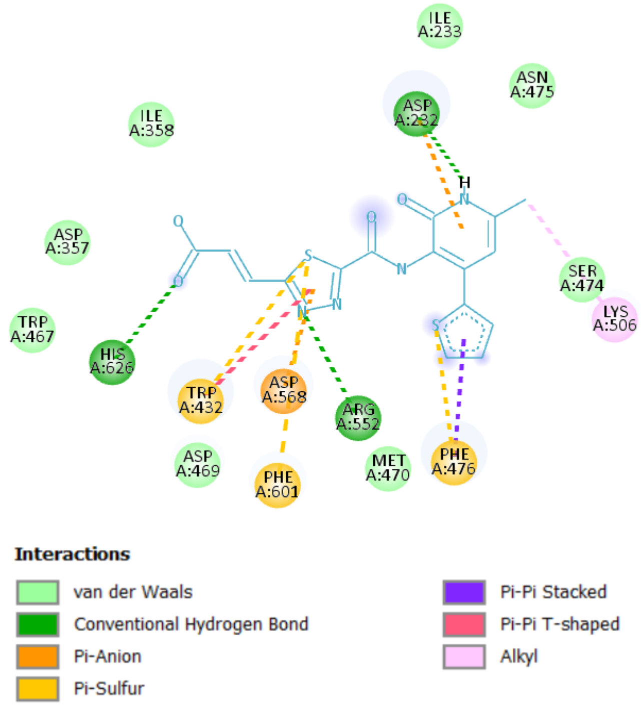
ILE A:233
ASN A:475
ASP A:282
ILE A:358
ASP A:357
TRP A:467
HIS A:626
SER A:474
LYS A:506
TRP A:432
ASP A:568
ARG A:552
ASP A:469
PHE A:601
MET A:470
PHE A:476
Interactions
van der Waals
Conventional Hydrogen Bond
Pi-Anion
Pi-Sulfur
Pi-Pi Stacked
Pi-Pi T-shaped
Alkyl

8c

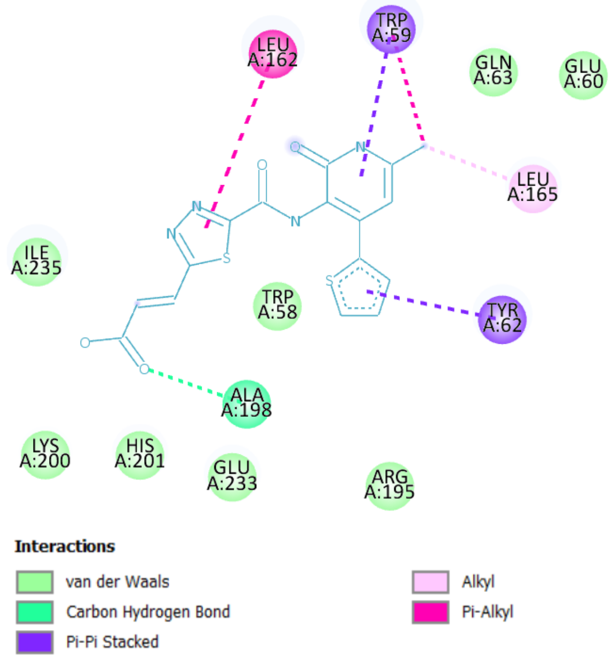
LEU A:162
TRP A:59
GLN A:63
GLU A:60
LEU A:165
ILE A:235
TRP A:58
TYR A:62
ALA A:198
LYS A:200
HIS A:201
GLU A:233
ARG A:195
Interactions
van der Waals
Carbon Hydrogen Bond
Pi-Pi Stacked
Alkyl
Pi-Alkyl

8c

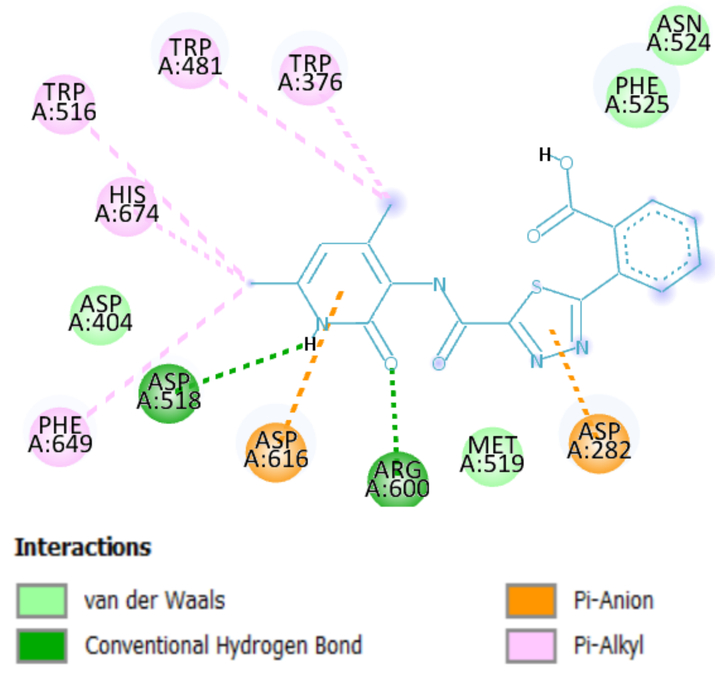
TRP A:516
TRP A:481
TRP A:376
ASN A:524
PHE A:525
HIS A:674
ASP A:404
ASP A:518
PHE A:649
ASP A:616
ARG A:600
MET A:519
ASP A:282
Interactions
van der Waals
Conventional Hydrogen Bond
Pi-Anion
Pi-Alkyl

9a

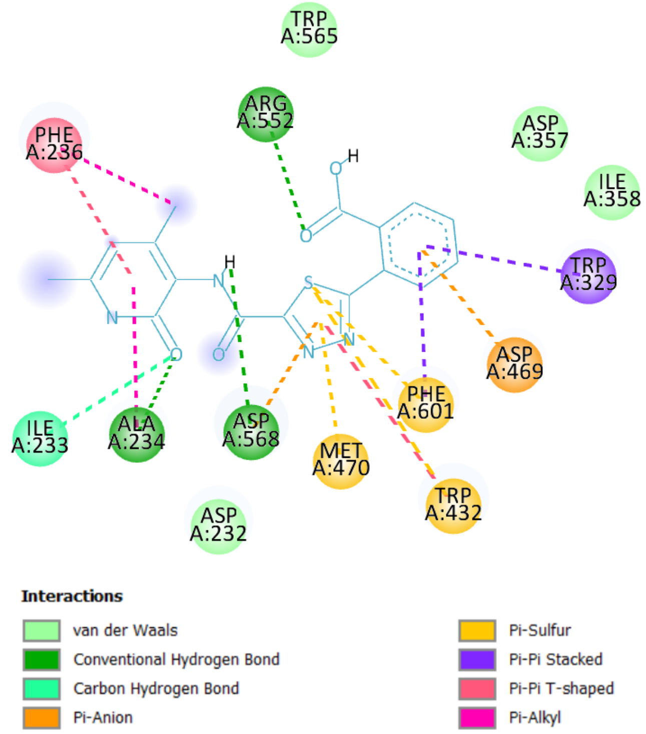
TRP A:565
ARG A:552
PHE A:236
ASP A:357
ILE A:358
TRP A:329
ASP A:469
PHE A:601
ILE A:233
ALA A:234
ASP A:568
MET A:470
TRP A:432
ASP A:232
Interactions
van der Waals
Conventional Hydrogen Bond
Carbon Hydrogen Bond
Pi-Anion
Pi-Sulfur
Pi-Pi Stacked
Pi-Pi T-shaped
Pi-Alkyl

9a

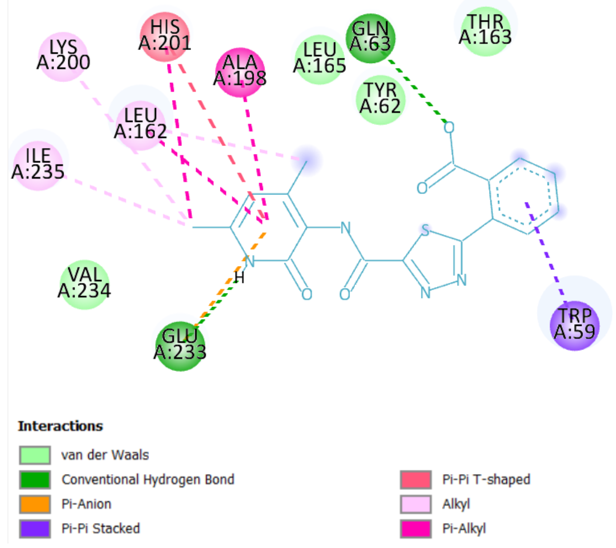
LYS A:200
HIS A:201
ALA A:198
LEU A:165
GLN A:63
THR A:163
TYR A:62
LEU A:162
ILE A:235
VAL A:234
GLU A:233
TRP A:59
Interactions
van der Waals
Conventional Hydrogen Bond
Pi-Anion
Pi-Pi Stacked
Pi-Pi T-shaped
Alkyl
Pi-Alkyl

9a

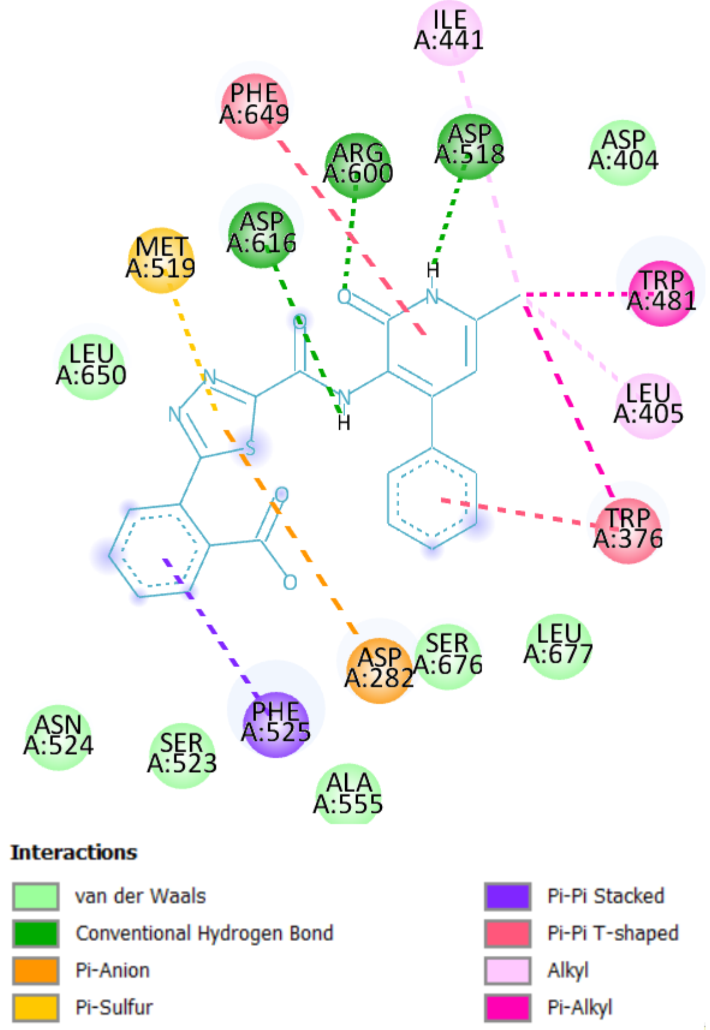
ILE A:441
PHE A:649
ARG A:600
ASP A:518
ASP A:404
MET A:519
ASP A:616
TRP A:481
LEU A:650
LEU A:405
TRP A:376
ASP A:282
SER A:676
LEU A:677
ASN A:524
SER A:523
PHE A:525
ALA A:555
Interactions
van der Waals
Conventional Hydrogen Bond
Pi-Anion
Pi-Sulfur
Pi-Pi Stacked
Pi-Pi T-shaped
Alkyl
Pi-Alkyl

9b

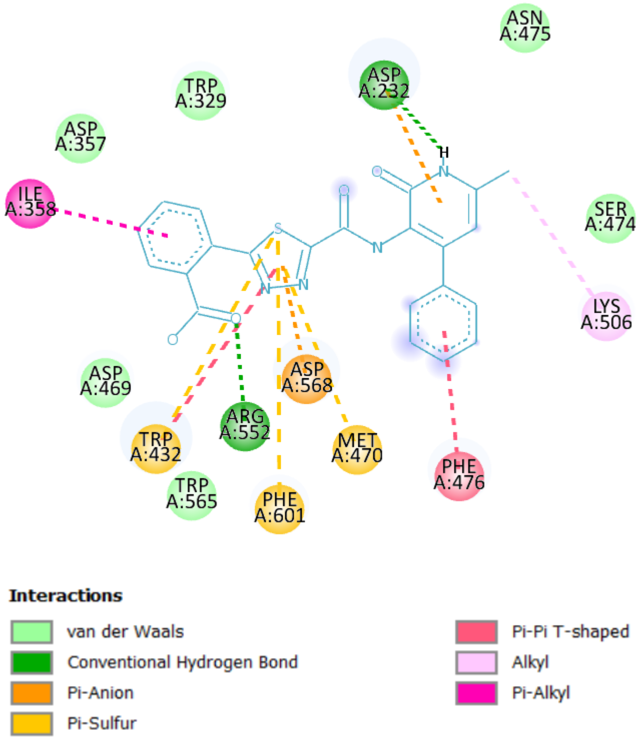
ASN A:475
TRP A:329
ASP A:232
ASP A:357
ILE A:358
SER A:474
LYS A:506
ASP A:469
ASP A:568
TRP A:432
ARG A:552
MET A:470
PHE A:476
TRP A:565
PHE A:601
Interactions
van der Waals
Conventional Hydrogen Bond
Pi-Anion
Pi-Sulfur
Pi-Pi T-shaped
Alkyl
Pi-Alkyl

9b

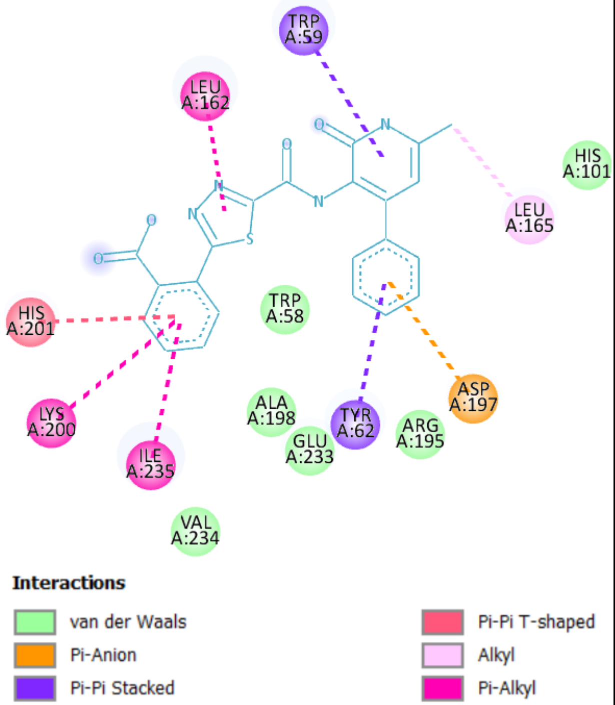
TRP A:59
LEU A:162
HIS A:101
LEU A:165
TRP A:58
HIS A:201
LYS A:200
ALA A:198
TYR A:62
ARG A:195
ASP A:197
GLU A:233
ILE A:235
VAL A:234
Interactions
van der Waals
Pi-Anion
Pi-Pi Stacked
Pi-Pi T-shaped
Alkyl
Pi-Alkyl

9b

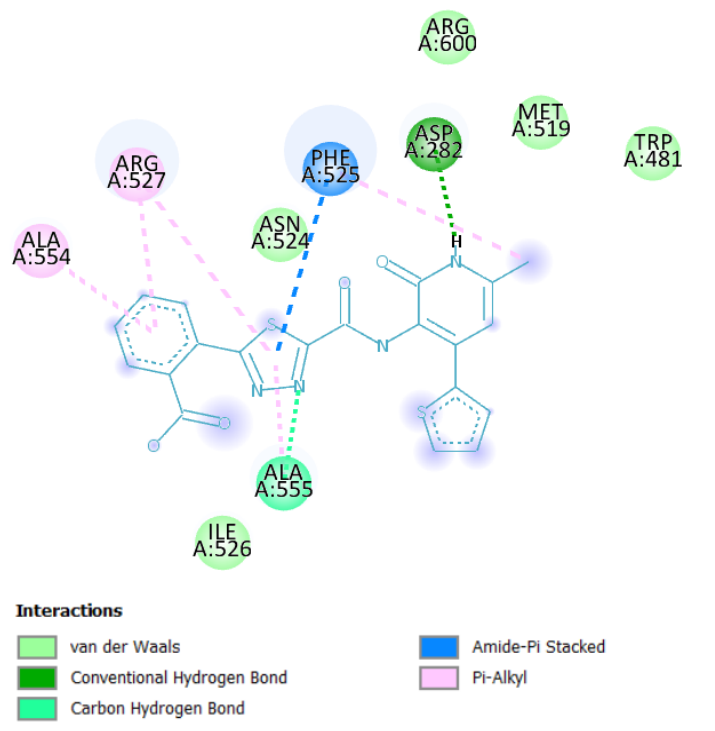
ARG A:600
MET A:519
TRP A:481
ARG A:527
PHE A:525
ASP A:282
ALA A:554
ASN A:524
ALA A:555
ILE A:526
Interactions
van der Waals
Conventional Hydrogen Bond
Carbon Hydrogen Bond
Amide-Pi Stacked
Pi-Alkyl

9c

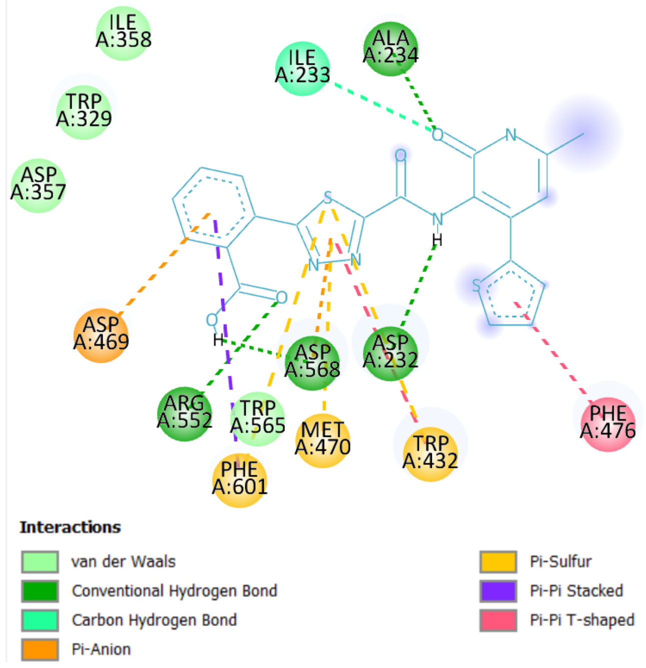
ILE A:358
ILE A:233
ALA A:234
TRP A:329
ASP A:357
ASP A:469
ASP A:568
ASP A:232
ARG A:552
TRP A:565
MET A:470
TRP A:432
PHE A:476
PHE A:601
Interactions
van der Waals
Conventional Hydrogen Bond
Carbon Hydrogen Bond
Pi-Anion
Pi-Sulfur
Pi-Pi Stacked
Pi-Pi T-shaped

9c

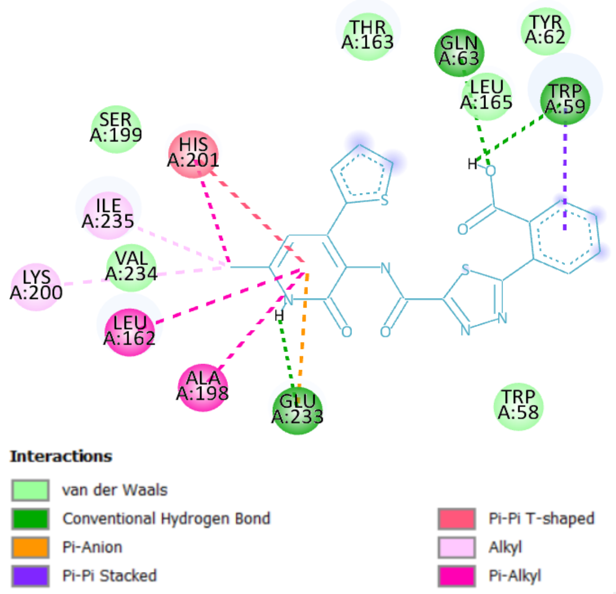
THR A:163
GLN A:63
TYR A:62
LEU A:165
TRP A:59
SER A:199
HIS A:201
ILE A:235
VAL A:234
LYS A:200
LEU A:162
ALA A:198
GLU A:233
TRP A:58
Interactions
van der Waals
Conventional Hydrogen Bond
Pi-Anion
Pi-Pi Stacked
Pi-Pi T-shaped
Alkyl
Pi-Alkyl

9c

Table S2. Basic amino acid interactions and H-bonds

Compound	Receptor	H-bond	Residual Amino acid Interactions	
			Pi-Sulfur/ Pi-Anion/Pi-Cation/Pi-Pi Stacked/ Pi-sigma/Pi-Pi T-shaped/Pi-Alkyl/ Alkyl/Amide-Pi Stacked/Pis interactions/Salt Bridge/ Attactive Charge/	Van-der Walls interactions
8a	3W37	ASP357	ALA234, ALA602, ALA628, PHE601, PHE236, ASP568, MET470, TRP432.	ARG552, HIS626, ASP469, ILE358, TRP329.
8b		ASP232, ASP568, ARG552, HIS626	TRP432, PHE601, PHE476, LYS506.	ASP469, TRP565, TRP467, MET470, SER474, ASN475.
8c		ASP469, ASP232, ARG552, HIS626	TRP432, PHE601, ASP568, PHE476, LYS506.	TRP565, TRP467, MET470, SER474.
8a	2QV4	HIS299, GLU233, ILE235, LYS200, VAL234	ALA198.	ARG195, ASP300, SER199, HIS201, HIS101.
8b		HIS201, HIS305, ALA198	LEU162, TYR62, LEU165, TRP59.	ASP300, TRP58, GLU233.
8c		HIS305	LEU162, TYR62, LEU165, TRP59, TRP58.	ASP300, HIS201.
8a	5NN8	ASP282; HIS674; ASP518	PHE525; ALA555; ASP282; ASP616; PHE646; MET519; TRP481.	SER523; ASN524; ARG281; ARG600; TRP376; TRP516.
8b		SER523; ASP616; MET519; ARG600; ASP518	ASP282; ASP616; TRP376; TRP481; PHE649; LEU405.	ALA555; PHE525; ARG281; SER676; LEU677.
8c		ASP282; ARG600; ASP518; HIS674	ALA555; ASP282; ASP616; PHE649; TRP481.	PHE525; SER523; ARG281; ASN524; MET519; ASP404; TRP516; TRP376.
7a	3W37	ASP232, ARG552, HIS626, ASP469	LYS506, PHE476, TRP432, PHE601, ASP568, ASP232.	ASN475, MET470, ALA628, TRP467, ASP357, TRP565, ILE358, SER474.
7b		ASP469, HIS626, ASP232.	TRP432, PHE601, ASP568, PHE476, ASP232, LYS506.	ALA628, ASP357, TRP467, ARG552, MET470, SER474, ASN475.
7c		ASP469, HIS626, ASP232.	MET470, TRP432, PHE601, ASP568, LYS506, ASP232.	ALA628, ASP357, TRP467, ARG552, PHE476, SER474, ASN475.
7a	2QV4	THR163, GLN63, ASP300, HIS299.	TYR62, LEU162, ALA198, ILE235, HIS201, GLU233.	TRP59, ARG195, HIS101, HIS305.
7b		GLU233, HIS305.	LEU162, ILE235, HIS201, ALA198.	LYS200, ARG195, ASP300, TRP58, LEU165, HIS101, THR163.
7c		GLU233, HIS305.	ILE235, HIS201, ALA198.	LYS200, ARG195, ASP300, TRP58, LEU162, HIS101.
7a	5NN8	ASP616, HIS674.	TRP376, PHE649, TRP481, ASP616, PHE525.	ARG600, ASP404, TRP516, ARG672.
7b		ASP616, ARG600.	ASP518, TRP376, PHE649, MET519, ASP282.	LEU677, SER676, TRP481, LEU405, ASP404, HIS674, ARG281.
7c		HIS674, ARG600.	PHE649, ASP616, TRP481, PHE525.	TRP516, ASP404, LEU405, ASP518, TRP376, MET519, ASP282, SER523.
8a	3W37	ASP469, HIS626, ARG552,	PHE601, ASP568, TRP432, ASP232, PHE476, LYS506.	ILE358, ASP357, TRP467, MET470, SER474, ASN475.

		ASP232		
8b		HIS626, ARG552, ASP232.	TRP432, PHE601, MET470, ASP568, PHE476, ASP232, LYS506.	ILE358, ASP357, TRP467, ASP469, SER474, ASN475.
8c		HIS626, ARG552, ASP282.	TRP432, ASP568, PHE601, PHE476, ASP282, LYS506.	ILE358, ASP357, TRP467, ASP469, SER474, ASN475, MET470, ILE233.
8a	2QV4	-	ILE235, ALA198, LEU162, THR163, TYR62, TRP58, HIS305, TRP59.	LYS200, HIS299, ASP300, LEU165, HIS101, GLU233, HIS201, ASP197.
8b		HIS201, ALA198, HIS305.	LEU162, TYR62, LEU165, TRP59.	TYR151, LYS200, GLU233, HIS101, ARG195, ASP197, ASP300, HIS299, TRP58, GLU60.
8c		ALA198.	LEU162, TYR62, LEU165, TRP59.	ILE235, LYS200, HIS201, GLU233, ARG195, GLU60, GLN63.
8a	5NN8	ASP518, MET519, ARG600, ASP616, ALA555, ASN524.	LEU405, ILE441, TRP481, ASP616, ASP282.	ASP404, TRP376, PHE649, PHE525.
8b		ALA555, ASP616, ASP518, ARG600.	ASP282, TRP376, ASP616, LEU405, TRP481, ILE441, PHE649.	ASN524, PHE525, LEU677, SER676, ASP404, MET519.
8c		ASP404, HIS674, ASP282.	MET519, TRP481, PHE649, ASP616, PHE525, ALA555, ASP282.	TRP516, TRP376, ARG600, ASN524.
9a	3W37	ALA234, ASP568, ARG552.	PHE286, ALA234, ASP568, MET470, TRP432, PHE601, ASP469, TRP329.	TRP565, ASP232, ASP357, ILE358.
9b		ASP232, ARG552.	TRP432, PHE601, PHE476, LYS506, MET470, ASP568, ASP232, ILE358.	ASP469, TRP565, SER474, ASN475, TRP329, ASP357.
9c		ARG552, ASP568, ASP232, ALA234, ILE233.	ASP469, PHE601, MET470, ASP568, TRP432, ASP232, PHE476	ILE358, TRP329, ASP357, TRP565.
9a	2QV4	GLU233, GLN63.	ALA198, HIS201, LEU162, LYS200, ILE235, GLU233, TRP59.	VAL234, LEU165, TYR62, THR163.
9b		-	LEU162, TYR62, LEU165, TRP59, HIS201, LYS200, ILE235, ASP197.	VAL234, ALA198, TRP58, GLU233, ARG195, HIS101.
9c		GLU233, TRP59, GLN63.	HIS201, ILE235, LYS200, LEU162, ALA198, GLU233, TRP59.	SER199, VAL234, TRP58, THR163, LEU165, TYR62.
9a	5NN8	ASP518, ARG600.	TRP376, TRP481, TRP516, HIS674, PHE649, ASP616, ASP282.	ASP404, MET519, PHE525, ASN524
9b		ARG600, ASP518, ASP616.	ASP282, PHE525, TRP376, LEU405, TRP481, ILE441, MET519, PHE649.	LEU677, SER676, ASP404, ALA555, SER523, ASN524, LEU650.
9c		ALA555, ASP282.	ARG527, ALA554, PHE525.	ASN524, ILE526, ARG600, MET519, TRP481.
